# Synthesis and in silico studies of hexahydroacridine derivatives as potential antitumor agents

**DOI:** 10.1038/s41598-026-68544-0

**Published:** 2026-09-09

**Authors:** Eman A. E. El-Helw, Sherif F. Hammad, Hassan A. Khatab, Youssef A. Said, Esmail M. El‐Fakharany, Ahmed I. Hashem

**Affiliations:** 1 Chemistry Department, Faculty of Science, Ain Shams University, Cairo, 11566 Egypt; 2 Pharmaceutical Chemistry Department, Faculty of Pharmacy, Capital University (Formerly Helwan University), Cairo, 11795 Egypt; 3 Medicinal Chemistry Department, Faculty of Pharmacy, Egypt-Japan University of Science and Technology (E-JUST), New Borg El Arab, 21934 Alexandria Egypt; 4 School of Biomedical Engineering, Harbin Institute of Technology (Shenzhen), Shenzhen, 518055 Guangdong China; 5 Biochemistry Department, Faculty of Science, Ain Shams University, Cairo, 11566 Egypt; 6 Protein Research Department, Genetic Engineering and Biotechnology Research Institute GEBRI, City of Scientific Research and Technological Applications, New Borg El Arab, 21934 Alexandria Egypt

**Keywords:** 1,8-Acridinediones, Antitumor activity, Telomerase inhibition activity, Molecular docking, Multicomponent reaction, Biochemistry, Cancer, Chemical biology, Chemistry, Computational biology and bioinformatics, Drug discovery

## Abstract

Despite advances in targeted cancer therapies, drug resistance and off-target toxicity remain major challenges, underscoring the need for new multi-targeted agents with improved selectivity. The present study evaluated the anticancer potential of newly synthesized hexahydro-1,8-acridinedione derivatives featuring strategic structural modifications at positions 9 and 10. The novelty of this work lies in the introduction of *N*-aryl substituents combined with C-9 biphenyl or 4-bromophenyl groups, a structural motif not previously reported for this chemotype. This dual modification was rationally designed to enhance π-π stacking interactions with kinase ATP-binding pockets while improving metabolic stability through fluorine substitution. Compounds were synthesized via a one-pot multicomponent reaction catalyzed by PTSA and characterized by IR, ^1^H/^13^C NMR, and LC–MS. Guided by structure-based design principles, five derivatives (**4a, 4d, 4f., 5a, 5b**) were evaluated for cytotoxicity against HSF, H460, A431, A549, and MDA-MB-231 cell lines. Compound **4d** emerged as the most potent, with an IC₅₀ of 17.91 ± 2.8 µg/mL against H460 lung cancer cells and a high selectivity index (SI = 20.7), in addition to exhibiting a considerable anti-telomerase activity. Molecular docking revealed strong binding affinities of **4d** with TOP2B (− 7.02 kcal/mol), p38 MAPK (− 7.05 kcal/mol), p53 (− 7.25 kcal/mol), and EGFR (− 6.55 kcal/mol), supported by MM-GBSA binding free energies (ΔG_bind_ ≈ − 42 kcal/mol for 4d–EGFR). Furthermore, 100-ns Molecular Dynamics simulations confirmed the stability of the **4d**-EGFR complex (RMSD ≈ 1.31 Å; persistent H-bonds with CYS773/GLN767). These findings establish compound **4d** as a promising multi-target lead scaffold, warranting further optimization toward selective anticancer therapeutics.

**Supplementary Information:**

The online version contains supplementary material available at https://doi.org/10.1038/s41598-026-68544-0.

## Introduction

Cancer remains a paramount global health challenge, characterized by uncontrolled cell proliferation and the ability to metastasize to distant organs^1^. According to the latest Global Cancer Observatory (GLOBOCAN) 2022 estimates, there were approximately 20 million new cancer cases and 9.7 million cancer deaths worldwide^1,2^. Despite advancements in chemotherapy and immunotherapy, the development of drug resistance and severe side effects necessitates the continuous discovery of novel therapeutic agents with improved efficacy and safety profiles^3^. Consequently, significant research efforts are currently directed toward identifying small molecules capable of targeting multiple signaling pathways involved in tumor progression^4^.

Recent computational and experimental studies have expanded the therapeutic paradigm of acridine scaffolds from single-target DNA intercalators to multi-targeted kinase inhibitors. Touqeer and Moutraji (2025) demonstrated that amide-functionalized acridines simultaneously bind EGFR, VEGFR-2, B-RAF, and PDGFR-α, offering a convergent strategy against heterogeneous tumor populations^5^. Complementarily, Gupta et al. (2025) established that strategic modifications at the N-position and C-9 position of acridine cores can fine-tune target selectivity without compromising DNA intercalation capacity^6^. Collectively, these studies reveal that the antitumor mechanism of modern acridines is target-dependent and modifiable by peripheral substitution patterns, a principle that directly informs our design strategy.

However, a critical gap persists: systematic exploration of *N*-aryl-9-aryl-substituted hexahydro-1,8-acridinediones, where the structure–activity landscape governing *multi-target profiles* within this scaffold, particularly the influence of bulky, electron-modulated aryl substituents at C-9 and N-10, warrants further systematic exploration. Recent reviews confirm that while 9-aryl and *N*-imide substitutions have been individually optimized^7,8^, the combined effect of *N*-aryl and bulky C-9 aryl groups on (i) target engagement, (ii) cancer cell selectivity, and (iii) multi-kinase inhibition profiles has not been systematically investigated. This gap is particularly significant because the simultaneous presence of *N*-aryl and C-9 biphenyl/4-bromophenyl groups may engender cooperative π-π stacking and lipophilic interactions that are unattainable with single-point modifications. Accordingly, the present study was designed to synthesize and evaluate a focused library of *N*-aryl-9-substituted-1,8-acridinediones, with the specific aim of identifying lead compounds with enhanced multi-target potency and improved selectivity indices against lung cancer cells.

Nitrogen-containing heterocycles constitute a privileged class of compounds in medicinal chemistry, serving as the backbone for numerous FDA-approved anticancer drugs^9^. Among these, acridine and acridinedione derivatives have garnered sustained interest due to their versatile biological activities, including antimalarial, antiviral, and potent antitumor properties^3,10,11^. Traditionally, the cytotoxicity of acridines was attributed primarily to DNA intercalation, which disrupts replication and transcription^12^. However, recent advancements (2023–2025) have expanded this understanding, demonstrating that modern acridine scaffolds can act as multi-targeted agents inhibiting key proteins such as Topoisomerase II beta (TOP2B), Epidermal Growth Factor Receptor (EGFR), p53, and p38 MAPK^6,13–18^. For example, acridine-mediated upregulation of p53 and Bax transcripts has been linked to mitochondrial apoptosis pathways, while downregulation of cyclin-dependent kinase mRNAs correlates with G2/M arrest. These mechanistic insights illustrate that acridine antitumor activity operates at multiple biological levels, from direct target binding to transcriptional reprogramming. Consequently, rational optimization of acridine scaffolds demands an integrated experimental-computational workflow that correlates structural modifications with both molecular docking profiles and phenotypic outcomes. This dual-validation approach, recently championed in heterocyclic anticancer lead optimization^19–22^, constitutes the central methodology of the present investigation.

In continuation to the multi-targeting nature of the modern acridine scaffolds through which they mediate their very selective antitumor activity, many of the rationally designed substituted acridine derivatives are known to interfere with telomerase-related mechanisms on various cancer cell lines^23–25^. In tumors, it is decisive to keep the limitless self-renewal capability of the neoplastic cells through maintenance of the chromosomal telomere length for the sake of overcoming the fate of cell senescence^26^. Hence, targeting telomerase activity is substantial in the search for potential anti-cancer agents^26^. The anti-hTERT (human telomerase reverse transcriptase) activity of such chemical species probably stems from their ability to stabilize the G-quadruplexes (G4s) structures formed from folding of the guanine-rich regions that are frequently encountered at the 3′ chromosomal ends (telomeres)^24,27,28^.

The 1,8-acridinedione ring, in particular, is recognized as a versatile scaffold for drug development^9,29,30^ with various biological functions, including anti-malarial activity^31,32^, potassium channel opening effects^10,33^, antitumor^34,35^, antitubercular, antiparasitic^11^, antimalarial, antiviral^36^, and analgesic effects^37^. While their activity is partly attributed to nucleic acid interactions^38,39^, structural optimization has proven pivotal in improving their pharmacological profiles. For instance, derivatives with modified substituents at the nitrogen atom have demonstrated enhanced DNA-binding affinity and cytotoxic properties^3,40^. It was reported that acridine derivative **I** exhibited remarkable potency against metastatic breast cancer cells^41^. However, the influence of alternative N-aryl and C-9 substitutions on target selectivity and mechanism remains underexplored, prompting the current investigation.

**Figure:**
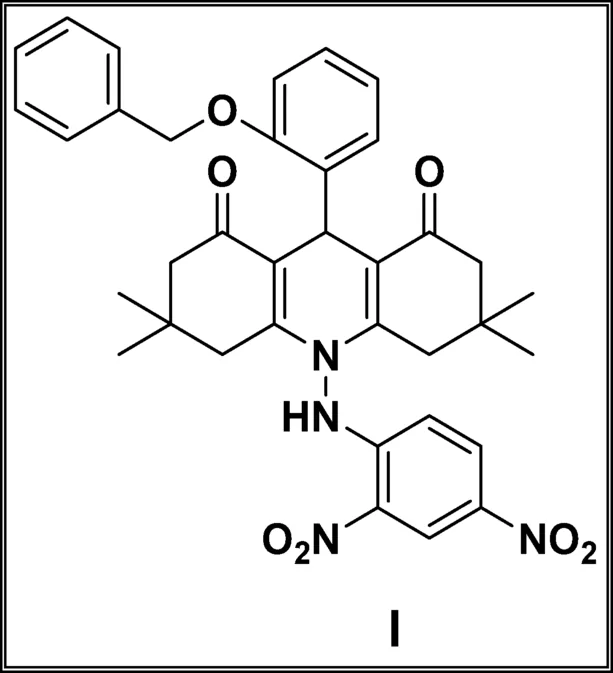

In a preceding investigation, we studied the synthesis of some acridine-1,8-dione derivatives having imide groups attached to the hetero-nitrogen atom. Evaluation of biological activity of these derivatives revealed that some of them have promising anticancer activity^9,16,17^. This encouraged us to continue our program aimed at identifying compounds of anticancer activities^9,42–55^.

Building on these advances, this study focuses on the design and synthesis of a new class of N-arylacridine-1,8-dione derivatives. We hypothesized that introducing specific aryl substituents at the nitrogen position would enhance target engagement and selectivity compared to our previously reported imide analogs. Herein, we report the synthesis and extensive antitumor evaluation of these new derivatives. Notably, preliminary evaluation identified compound **4d** as a potent lead, exhibiting significant cytotoxicity against H460 lung cancer cells with a high selectivity index (SI = 20.7). This profile distinguishes the scaffold from standard EGFR-targeted agents and supports its potential as a new multi-target anticancer candidate warranting further optimization.

## Rationale and design

In our pursuit of developing potent antitumor agents, we employed a structure-based drug design strategy inspired by Amsacrine (m-AMSA), a clinically approved anticancer drug whose pharmacophoric features are well-established for cancer chemotherapy. Our design preserved Amsacrine’s planar polyaromatic chromophore, which is critical for DNA intercalation and topoisomerase II inhibition, key mechanisms underlying its antitumor efficacy^56^.

The 1,8-acridinedione core possesses structural features associated with dual anticancer mechanisms, including kinase inhibition and oxidative stress induction^57–59^. Its inherent electronic character is defined by an enaminone system (N-10–C-9–C-8=O) that functions as a redox-active motif, enabling electron transfer processes that generate reactive oxygen species (ROS) and disrupt cancer cell redox homeostasis. Building on these mechanistic attributes, our design introduces N-aryl and C-9 aryl substituents to the acridinedione core to fine-tune target engagement, specifically, to enhance π–π stacking with kinase ATP-binding domains and improve metabolic stability through strategic fluorination. This modification strategy is supported by recent SAR studies demonstrating that peripheral arylation at the N-10 and C-9 positions of acridinediones modulates kinase selectivity^6,60^.

**Fig. 1:**
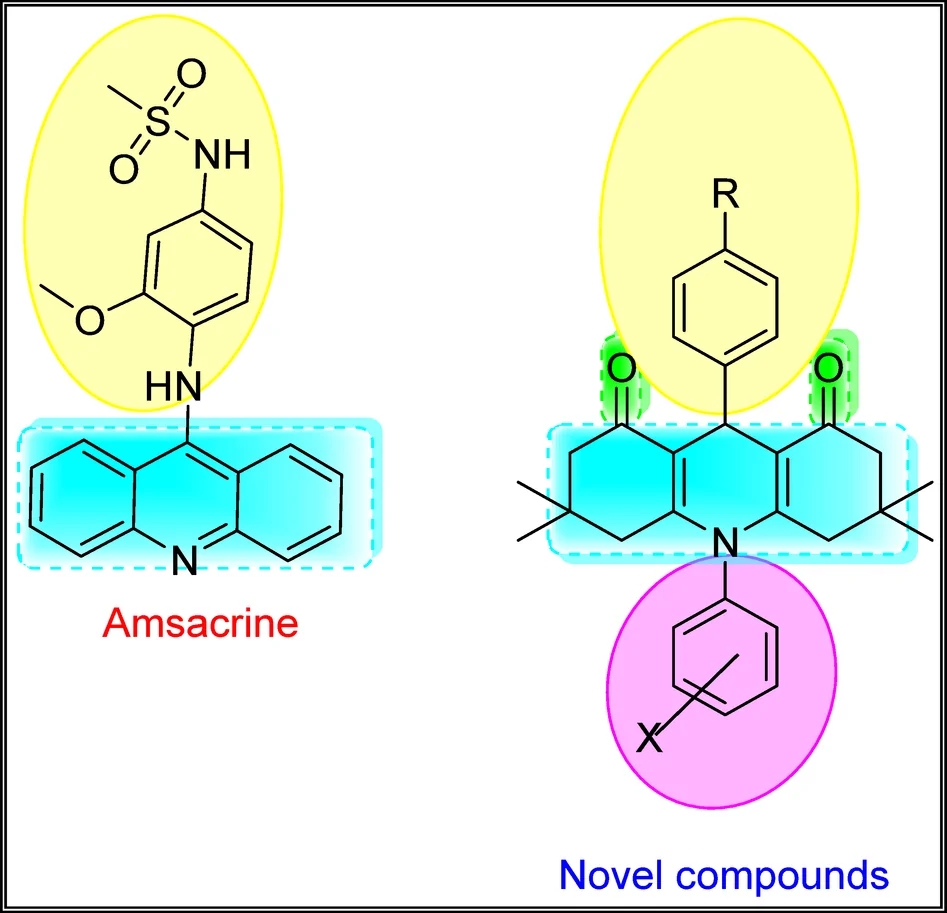
Chemical structures of Amsacrine and the designed N-aryl-9-substituted-1,8-acridinedione derivatives, highlighting key pharmacophoric features.

Central to this approach was the incorporation of the 1,8-acridinedione scaffold, where the two carbonyl groups at positions 1 and 8 serve as hydrogen bond acceptors, potentially forming critical interactions with amino acid residues (e.g., Arg, Lys, Asn) in the ATP-binding pocket of kinases or the DNA-enzyme interface of topoisomerases to enhance binding affinity and target selectivity. Complementing this core, the introduction of aryl groups at the nitrogen atom was designed to modulate electronic distribution across the heterocyclic core and provide opportunities for π-π stacking interactions with aromatic residues in the target protein binding site, while simultaneously influencing lipophilicity and cellular permeability. Within these aryl systems, strategic fluorine substitution was implemented based on well-documented benefits in anticancer drug design, including enhanced metabolic stability through the blockade of oxidative metabolism sites, improved membrane permeability and bioavailability via modulation of lipophilicity, increased binding affinity through favorable electrostatic interactions, and the potential formation of C-F···H-N or C-F···H-C hydrogen bonds with target proteins^7,61^. Beyond fluorination, diverse substituent patterns involving electron-donating and electron-withdrawing groups were systematically varied to establish SAR and optimize the balance between potency, selectivity, and physicochemical properties. These design elements, as depicted in Fig. 1, were subsequently validated through molecular docking studies to predict binding modes, identify key interactions with target proteins (e.g., EGFR, TOP2B), and prioritize compounds for synthesis and biological evaluation. This rational, mechanism-informed approach aims to yield novel acridinedione derivatives with enhanced antitumor activity and improved therapeutic indices compared to existing scaffolds.

## Results and discussion

### Chemistry

**Scheme 1:**
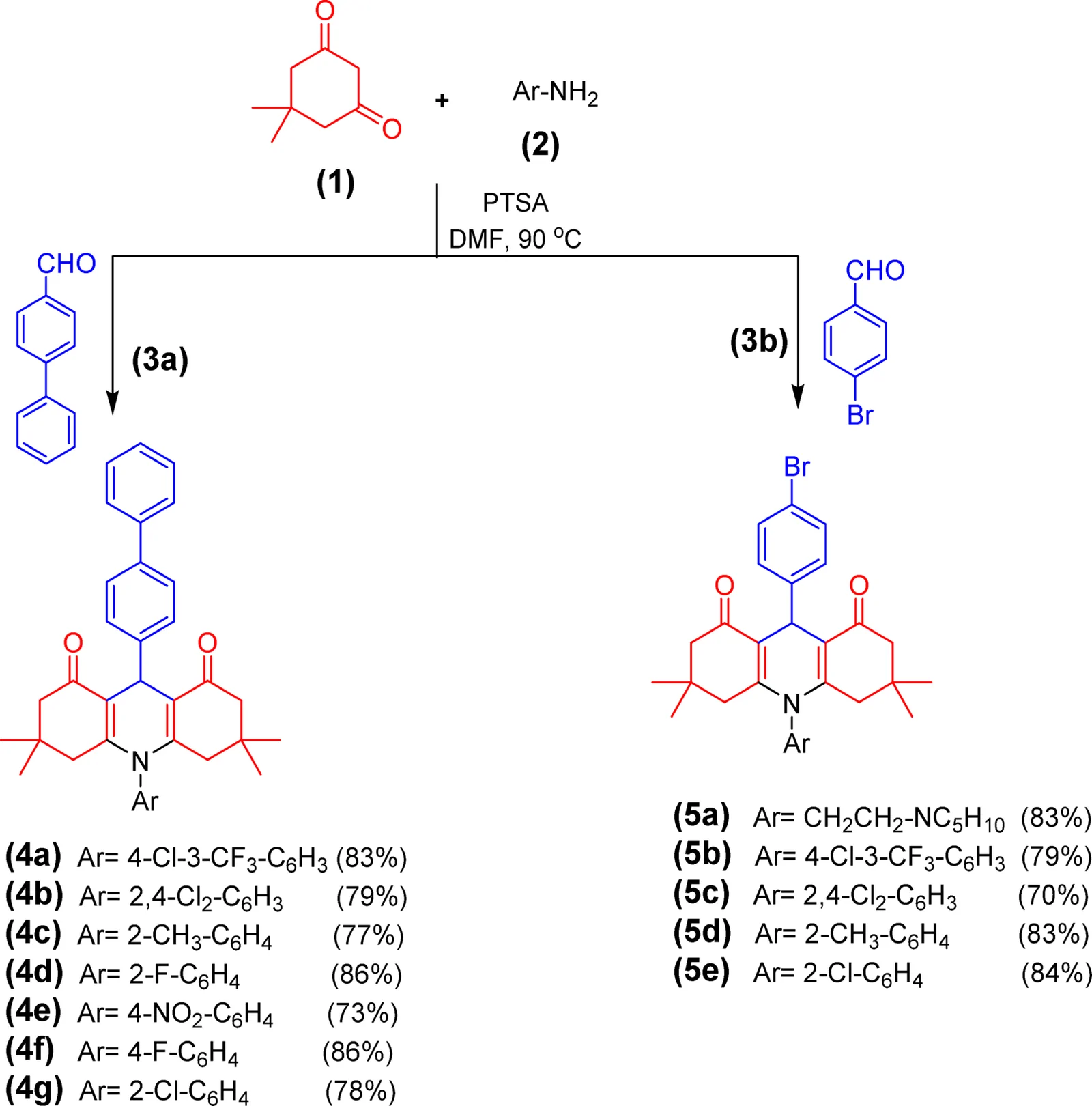
Synthesis of *N*-aryl-9-substituted-1,8-acridinediones.

As a part of our ongoing investigation into the discovery of effective new anticancer compounds, the present work aimed to study the influence of the 1,8-acridinedione scaffold as an anticancer agent. We planned to insert two aromatic moieties at positions 9 and 10. Thus, derivatives of 1,8-acridinedione **4a-4g** and **5a-5e** were produced through a one-pot reaction of dimedone **1** and aromatic amines **2** with [1,1′-biphenyl]-4-carbaldehyde (**3a**) or 4-bromobenzaldehyde **3b** in dimethylformamide (DMF) involving PTSA (*p*-toluenesulfonic acid) as a catalyst (Scheme 1) to get pure compounds and good yield (70%-86%). The chemical structures of these synthesized derivatives were verified using IR, ^1^H and ^13^C NMR, and mass spectral analysis.

The use of [1,1′-biphenyl]-4-carbaldehyde in the synthesis of *N*-aryl-1,8-acridinedione derivatives (compounds **4a–4 g**) was motivated by its ability to introduce a bulky, electron-rich biphenyl moiety at position-9 of the acridinedione scaffold. This structural feature enhances the planarity and π-π stacking interactions of the molecule, which are critical for DNA intercalation and binding to cancer-related proteins such as TOP2B and EGFR. The biphenyl group was selected to improve the compounds’ lipophilicity and binding affinity, potentially increasing their antitumor efficacy and selectivity, as demonstrated by the potent activity of compound **4d** against H460 lung cancer cells^25^.

The IR spectra of compounds **4a–4 g** and **5a–5e** displayed characteristic absorption bands consistent with the acridinedione framework: aromatic C–H stretches at 3065–3021 cm⁻^1^, aliphatic C–H stretches at 2962–2942 cm⁻^1^, strong carbonyl (C=O) stretching vibrations at 1648–1659 cm⁻^1^, aromatic C=C skeletal vibrations at 1590–1480 cm⁻^1^, and C–N stretching bands at 1290–1330 cm⁻^1^. Collectively, these diagnostic signals corroborate the proposed molecular structures of the synthesized derivatives. In turn, their ^1^H NMR spectra exhibited singlet signals corresponding to methyl protons of cyclohexenone rings, observed in the range δ 0.73–0.94 ppm. Doublet signals were viewed in the range of δ 1.53–2.25 ppm, representing the CH_2_ group protons of the cyclohexenone rings. Additionally, a singlet signal for CH proton was detected in δ 5.04 ppm of acridinedione, while signals for aromatic protons were perceived in the range of δ 7.08–8.03 ppm.

The ^13^C NMR spectrum revealed two carbonyl carbons of cyclohexenone rings at a chemical shift of 195.69 ppm, consistent with sp^2^ hybridization due to the presence of the double bond. The spectrum identified multiple aromatic carbons in the range of δ 113.67 ppm to δ 150.14 ppm. These include an aromatic carbon biphenyl ring, a 4-bromophenyl ring, and other substituted aromatic rings. The spectrum also showed aliphatic carbons at various chemical shifts that correlate with their hybridization (sp^3^) and their proximity to functional groups. The deshielding effect of the carbonyl group is evident when it is bonded to the CH₂ group, which resonates the latter CH_2_ group at a higher chemical shift compared to the other aliphatic carbons.

In ^13^C NMR, the fluorine atom in compounds causes benzene carbon signals to split into doublets. The coupling constants vary depending on the distance between the fluorine and the carbons. Notably, fluorine’s influence is detectable even when separated by more than three bonds, reflecting its significant long-range electronic effects^62^. In the ^13^C NMR spectrum of compound **4d**, the C-F carbon exhibited doublet resonances at 160.2 ppm and 157.8 ppm, with a J(C-F) coupling constant of 245 Hz, in addition to signals of ortho, meta, and para carbons at 113.8, 140.5, and 126.1 with *J* = 27, 7, and 2 Hz, respectively. Also, in the ^13^C NMR spectrum of compound **4f.**, the C-F carbon exhibited doublet resonances at 163.56 ppm and 161.10 ppm, with a J(C-F) coupling constant of 246 Hz. In the ^19^F NMR spectrum of compound **4a**, the fluorine resonated at δ − 61.24 ppm as a singlet. Also, in the ^19^F NMR spectrum of compound **4f.**, the fluorine resonated at δ − 111.64 ppm as a singlet.

Based on the mass spectral analysis, it could be concluded that compound (**4c**) is a main compound in the sample, with a retention time of 0.68 min. and a mass-to-charge ratio of 548. Additionally, the expected MS (ESI^+^) *m/z* for C_35_H_34_N_2_O_4_ [M + H]^+^: was found to be 548.25, which indicates the possible molecular formula of the compound.

A significant advantage of the present complexes is their facile synthesis via a one-pot reaction. This approach not only simplifies the synthetic process but also reduces the likelihood of side reactions. Additionally, the reaction mixture can be poured directly into water, enabling straightforward work-up procedures and minimizing the use of organic solvents or complex purification steps. These features enhance the practicality of the method for large-scale applications. Acridinedione compounds are stable under normal conditions, making them ideal for various medicinal applications. Their stability supports their use in developing antimicrobial, anticancer, and anti-inflammatory drugs^60^.

**Scheme 2:**
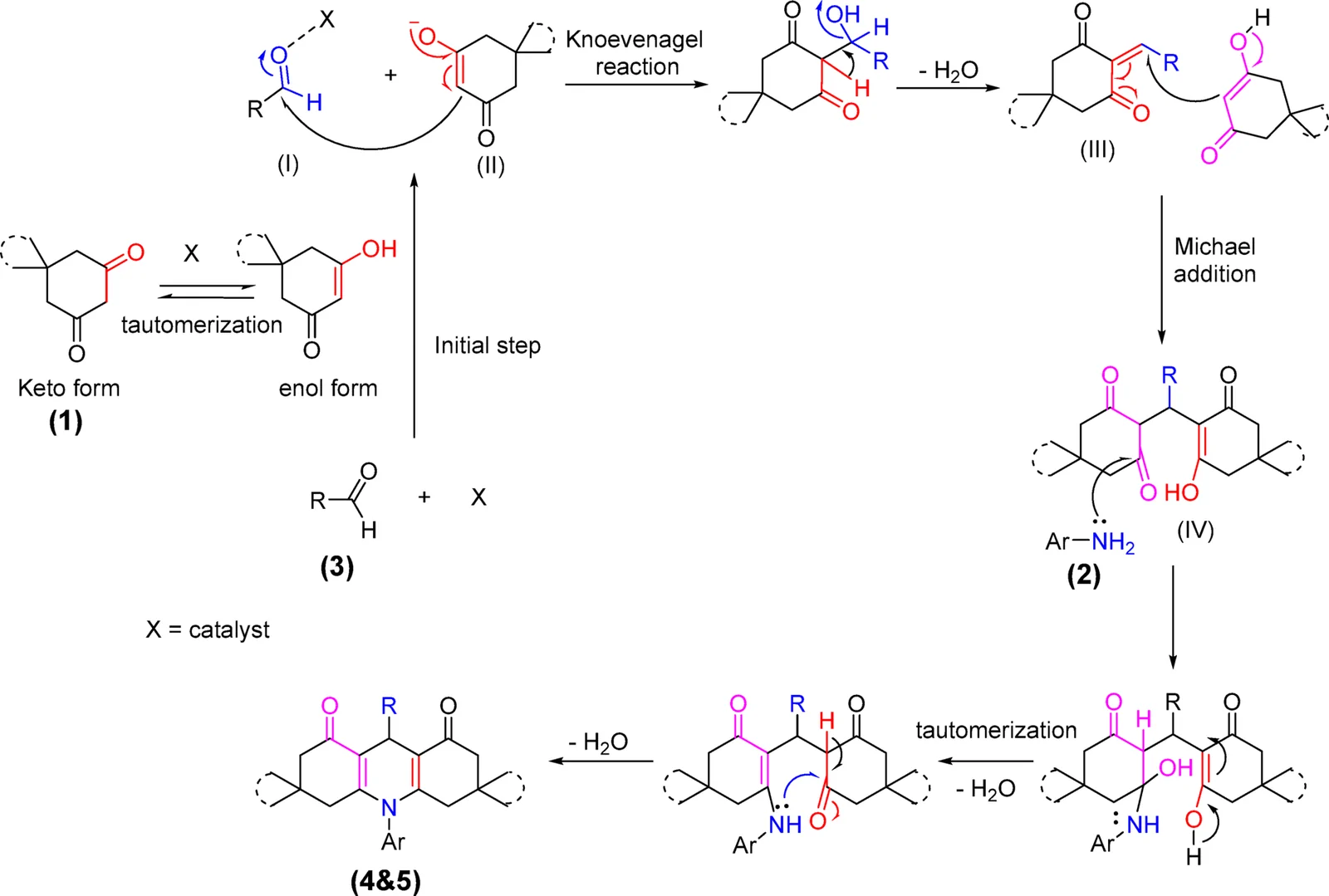
Proposed mechanistic pathway, illustrating the step-by-step formation of the compounds **4** and **5**.

The mechanistic pathway illustrating the formation of compounds **4** and** 5** is depicted in (Scheme 2). Initially, the aldehydic carbonyl group **3** is activated by a catalyst, reagent, or solvent (X), generating an electrophilic intermediate (I). This activation enhances the likelihood of faster nucleophilic addition. Subsequently, another activated 1,3-dicarbonyl compound (II) reacts with electrophile (I), forming intermediate (III). The active methylene group of an additional 1,3-dicarbonyl compound then reacts with intermediate (III), producing intermediate (IV). Following this, a nucleophilic attack occurs as the nitrogen atom of an amine (2) reacts with the carbonyl group of intermediate (IV). Cyclization and imine-enamine tautomerization subsequently take place. Finally, the removal of a water molecule leads to the formation of the desired acridine-1,8-diones (**4** and **5**)^63^.

Although reaction conditions in literature may vary depending on the specific experimental setup and reactants employed, in the present study, a uniform protocol was maintained for all derivatives. Specifically, *p*-toluenesulfonic acid (PTSA) was used as the catalyst in dimethylformamide (DMF) at 90 °C, ensuring consistent reaction conditions across the synthesized series (**4a–4 g** and **5a–5e**).

### Antitumor activity

The selection of compounds **4a, 4d, 4f., 5a,** and** 5b** was driven by SAR strategy focusing on electronic and steric variations. Specifically, fluorinated derivatives (**4a, 4d, 4f.,** and** 5b**) were selected because fluorine substitution is known to enhance metabolic stability, membrane permeability, and binding affinity through electrostatic interactions^61,64^. Compound **5a** was selected due to the presence of a piperidine moiety, which is recognized for its ability to interact with DNA and inhibit key enzymes involved in cancer proliferation^62^. Furthermore, compound** 5b** was strategically selected to evaluate the SAR of the position-9 substituent by comparing the 4-bromophenyl group against the [1,1′-biphenyl]-4-yl moiety present in compound** 4a**. Since both compounds share an identical substituent at position 10, this pairwise comparison isolates the steric and electronic effects of the position-9 aryl group, enabling us to determine whether the bulky biphenyl system or the halogenated bromophenyl group offers superior antitumor potency and selectivity^65^.

The cytotoxicity of the prepared compounds is thought to result from their ability to bind to DNA, disrupting its structure. This interference inhibits replication and transcription processes, ultimately leading to cell death^11,66^.

Both the current study and the work by Alvala et al. ^41^ explore the anticancer potential of 1,8-acridinedione derivatives, though with distinct mechanistic focuses driven by structural variations. Alvala et al. identified acridinediones primarily as SIRT1 inhibitors, reporting potent activity for their lead derivative (Compound I: 9-(2-(benzyloxy)phenyl)-10-((2,4-dinitrophenyl)amino)-3,3,6,6-tetramethyl-3,4,6,7,9,10-hexahydroacridine-1,8(2H,5H)-dione) against MDA-MB-231 breast cancer cells (IC₅₀ ≈ 1 µg/mL). In alignment with this, our compound 4d also demonstrated activity against MDA-MB-231 cells (IC₅₀ = 35.04 ± 2.5 µg/mL), while exhibiting its highest potency against H460 lung cancer cells (IC₅₀ = 17.91 ± 2.8 µg/mL).

### Telomerase inhibition activity

**Fig. 2:**
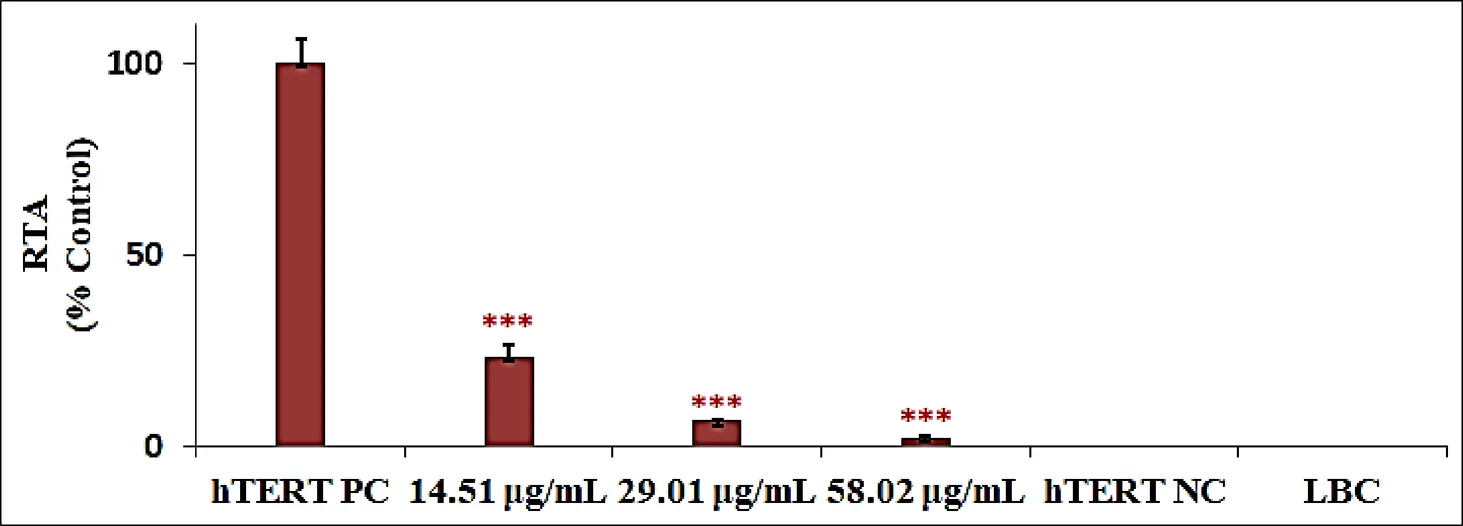
Effect of 4d derivative on telomerase activity in A549 cell line. Values were plotted as mean ± SEM (n = 3). RTA, Relative Telomerase Activity; hTERT NC, human Telomerase Reverse Transcriptase Negative Control; hTERT PC, human Telomerase Reverse Transcriptase Positive Control; LBC, Lysis Buffer Control. notation: low significance * (*p* < *0.05*), moderate significance ** (*p* < *0.01),* and high significance *** (*p* < *0.001*).

In the endeavor of scrutinizing the potential mode of action of the newly designed and synthesized class of N-arylacridine-1,8-dione derivatives as a potent antineoplastic, the compound **4d** emerged as a lead compound, being the most potent derivative, especially against the A549 lung adenocarcinoma cell line, reporting an IC₅₀ value of 29.01 ± 3.2 µg/mL and a tumor selectivity index (SI) value of 12.8. The derivative inhibited telomerase enzyme activity in the tested cell line as inferred from the implemented Q-TRAP assay, a commonly relied on method for the fundamental metric of relative telomerase activity (RTA)^67,68^. Data were statistically analyzed through one-way ANOVA followed by Tukey’s post-hoc test. Results showed that 4d significantly (*p* = 0.000) inhibited hTERT in the A549 cancer cell line by 76.7 ± 5.3%, 93.2 ± 0.6%, and 97.8 ± 1.7% when applied at the concentrations of ½IC_50_, IC_50_ and 2IC_50_, Fig. 2.

It is well known that most normal cells exhibit very little telomerase activity; nevertheless, over 85% of neoplastic cells exhibit telomerase reactivation, which preserves telomere length and is thought to be responsible for their longevity. The idea that the lead compound **4d** might specifically block telomerase activity in cancerous cells was sparked by this significant observation. The prominent anti-hTERT activity of the **4d** derivative as a polycyclic acridine derivative could be attributed to its flat aromatic surface, which is deemed to be optimal for π-π stacking with the G-quartet planar structure, forming a stable ligand-quadruplex complex^69^.

Critically, the structural differences between compound **I** and our series dictate a fundamental mechanistic shift from single-target SIRT1 inhibition to broad-spectrum polypharmacological engagement. Compound **I** features a 2,4-dinitrophenylamino group at N-10 and a benzyloxy moiety at C-9, which together create a precise geometric and electronic complementarity for the conserved hydrophobic groove of the SIRT1 catalytic domain. Through this single-target mechanism, Compound I stabilizes p53 acetylation at Lys382 since SIRT1 normally deacetylates p53, suppressing its tumor-suppressor function, thereby restoring p53-dependent transcription of pro-apoptotic targets including Bax and p21, and inducing caspase-dependent cell death.

This SIRT1–p53 axis is now firmly established as an anticancer strategy: pharmacological SIRT1 inhibition consistently elevates acetylated p53, triggering apoptosis across multiple cancer cell types^70–72^. However, the very structural precision that makes single-target agents potent also limits them: SIRT1-specific inhibitors show variable activity in tumor backgrounds with p53 mutations or deletions, and cancer cells can upregulate parallel survival pathways to compensate for single-node blockade^73,74^. In contrast, our compound **4d** was deliberately designed as a multi-target (polypharmacological) agent. Replacing the 2,4-dinitrophenylamino group at N-10 with a 2-fluorophenyl substituent, and the benzyloxy moiety at C-9 with an extended biphenyl system, dismantles the rigid geometry required for SIRT1-specific binding while creating a broader, more flexible interaction surface compatible with structurally diverse oncogenic targets.

Molecular docking confirmed that **4d** engages four distinct proteins: TOP2B (− 7.02 kcal/mol), p38 MAPK (− 7.05 kcal/mol), p53 (− 7.25 kcal/mol), and EGFR (− 6.55 kcal/mol), with 100-ns MD simulations further confirming a stable 4d–EGFR complex (RMSD ≈ 1.31 Å; persistent interactions at CYS773, MET769, and ARG817). The mechanistic advantage of this polypharmacological profile over single-target inhibition is well-supported by the literature: cancer is an inherently pathway-redundant disease, and drugs against individual targets frequently fail because tumor cells activate compensatory signaling routes to maintain proliferation^74,75^. This is especially relevant for TOP2B and EGFR: EGFR overexpression is a documented resistance mechanism against TOP2B-targeted therapies, since EGFR signaling can sustain proliferative output even when DNA damage is induced by topoisomerase inhibition^76^. Similarly, p38 MAPK is specifically activated as a compensatory survival signal in response to EGFR inhibition in lung and bladder cancer models^77^.

By simultaneously inhibiting all four targets, compound 4d blocks the primary cytotoxic axis (TOP2B → DNA double-strand breaks → p53-mediated apoptosis) while preemptively suppressing the two most clinically relevant escape routes (EGFR-mediated proliferative rescue and p38-mediated stress survival signaling). This mechanistic architecture directly explains compound **4d**’s markedly superior selectivity index (SI = 20.7 against H460 lung cancer cells) and distinguishes it from compound **I**, whose single-target SIRT1 mechanism, while potent against wild-type p53 breast cancer cells, offers no barrier to resistance through pathway compensation. These four targets were strategically selected based on literature precedents demonstrating that acridine derivatives modulate their mRNA expression levels, collectively covering vital signalling axes: p53^78,79^ and p38^80,81^ (regulating cell cycle arrest and apoptosis) alongside TOP2B^82–84^ and EGFR^85,86^ (governing DNA transcription and proliferative signaling).

Mechanistically, TOP2B inhibition induces DNA double-strand breaks that activate p53-mediated apoptosis, while EGFR overexpression is frequently associated with resistance to TOP2B-targeted therapies; thus, their simultaneous modulation represents a rational polypharmacological strategy to overcome compensatory survival pathways. Our docking analyses further validated this target selection by confirming favorable binding geometries and interaction energies between the synthesized compounds and the catalytic domains of these proteins. Collectively, these findings underscore the versatility of the acridinedione scaffold, where strategic structural modifications at positions 9 and 10 can fine-tune target engagement, from SIRT1-specific inhibition to multi-target kinase and topoisomerase modulation, as evidenced by the improved selectivity index (SI = 20.7) observed for compound 4d in cancer versus normal cell lines.

The effects of compounds **5a, 5b, 4a, 4d**, and **4f.** on cell viability were evaluated across multiple cell lines, including human skin fibroblasts (HSF), lung cancer cells (H460), skin cancer cells (A431), lung cancer cells (A549), and breast cancer cells (MDA-MB-231) by means of MTT assay^4^. Concentrations ranging from 0 to 200 µg/mL were tested for each compound after treatment for 48 h. Results were compared to untreated control cells and are expressed as mean ± standard error of the mean (SEM). The results showed varying impacts on the viability of both normal (HSF) and cancer cell lines. Notably, compound **4d** demonstrated significant efficacy against lung cancer cells (H460), as depicted in Fig. S1^16^.

All tumor cell lines were treated with compounds (**5a, 5b, 4a, 4d**, and **4f.**) at their respective IC_50_ doses for a duration of 48 h, alongside untreated control cells. The data illustrated by Fig. S2A, (Table 1), represents the IC_50_ values for these compounds across various cell types, including (HSF), (H460), (A431), (A549), and (MDA-MB-231). The IC_50_ value indicates the concentration at which a compound inhibits in vitro 50% of cell growth. Notably, each compound demonstrates different IC_50_ values across the various cell types^87^.

**Table 1:** The IC_50_ values of the prepared derivatives **5a, 5b, 4a, 4d**, and **4f.** (μg/mL) represent the half maximal inhibitory concentration, with mean values presented alongside the standard error of the mean (SEM).

Compds	Normal (HSF) cells		Lung (H460) Cells		Skin (A431) Cells		Lung (A549) Cells		Breast (MDA) Cells	
5a	115.9	± 4.3	29.05	± 3	49.42	± 3.2	64.26	± 2.5	48.17	± 2.7
4a	189.1	± 2.7	34.65	± 2.9	27.42	± 3.2	48.3	± 3.4	27.99	± 2.3
5b	173.5	± 3.9	28.45	± 3.1	22.54	± 3.6	51.66	± 2.3	55.44	± 2.9
4d	371.6	± 2.3	17.91	± 2.8	36.48	± 3.2	29.01	± 3.2	35.04	± 2.5
4f.	282.6	± 2.6	38.62	± 2.3	24.23	± 4.2	74.71	± 2.7	25.03	± 2.8
Etoposide	~ 18.88^88^		~ 3.59^89^		NA		~ 0.736^90^		~ 22.36^91^	
Sorafenib	~ 1.86^92^		~ 8.37^93^		NA		~ 2.75^94^		~ 2.75^95^	
Erlotinib	~ 4.3^96^		> 11.8^93^		~ 0.61^97^		~ 12.47^98^		~ 2.16^99^	

NA, Not available.

For example, compound **4d** exhibits the lowest IC_50_ values in H460 (17.91 ± 2.8), indicating its high potency in preventing the growth of this cell type. Similarly, compound **4f.** displays the lowest IC_50_ values in MDA-MB-231 (25.03 ± 2.3) and A431 (24.23 ± 4.2), highlighting its effectiveness in preventing the growth of these cell types.

The Selectivity Index (SI) values indicate a compound’s ability to selectively deter cancer cells while careful normal cells. Higher SI values reflect greater selectivity towards cancer cells. As shown in Fig. S2B (Table 2), compound **4d** exhibits the highest selectivity towards H460 with SI values of 20.7. Compound **4f.** demonstrates mid selectivity towards MDA-MB-231 and A431 with SI values of 11.3 and 11.7, respectively.

**Table 2:** Selectivity index (SI)* values, indicating the compounds’ selectivity towards cancer cells versus normal cells, are presented as mean values.

Compds	Lung (H460) Cells	Skin (A431) Cells	Lung (A549) Cells	Breast (MDA) Cells
5a	4.0	2.3	1.8	2.4
4a	5.5	6.9	3.9	6.8
5b	6.1	7.7	3.4	3.1
4d	20.7	10.2	12.8	10.6
4f.	7.3	11.7	3.8	11.3
Etoposide	5.3	NA	25.7	0.84
Sorafenib	0.22	NA	0.68	0.19
Erlotinib	0.36	7.1	0.34	2.0

NA, Not available; *SI = IC₅₀ (normal cells)/IC₅₀ (tumor cells).

To better contextualize the therapeutic potential of these derivatives, the cytotoxicity of the most potent compound **4d** was benchmarked against standard clinically used anticancer agents. Notably, compound **4d** exhibits potency against H460 lung cancer cells (IC₅₀ = 17.91 ± 2.8 µg/mL) that falls within the range reported for clinically used EGFR inhibitors such as Erlotinib in wild-type models (IC₅₀ ≈ 10–20 µg/mL)^100,101^.

### Molecular docking

Molecular docking analyses were conducted for five compounds (**5a, 5b, 4a, 4d**, and **4f.**) using crystal structures obtained from the Protein Data Bank (PDB IDs: 3QX3 for TOP2B, 3HEG for p38, 3JZK for p53, and 1M17 for EGFR. These four targets were selected based on literature precedents demonstrating that acridine derivatives modulate their mRNA expression levels, collectively covering vital signaling pathways: p53^78,79^ and p38^80,81^ (regulating cell cycle and apoptosis) and TOP2B^82–84^ and EGFR^85,86^ (governing DNA transcription and cell division). Mechanistically, TOP2B inhibition induces DNA damage that activates p53-mediated apoptosis, while EGFR overexpression is often associated with resistance to TOP2B inhibitors; thus, their simultaneous inhibition represents a strategic anticancer approach. Our molecular docking analyses further validated this selection by confirming effective binding interactions between the synthesized compounds and these specific proteins.

The binding affinities of every compound towards the respective target proteins were calculated and compared with a reference ligand known for its high-affinity binding to the proteins. The predicted binding poses of every compound were evaluated by employing the RMSD (root mean square deviation) metric to assess their similarity to the crystal structures of the protein–ligand complexes (refer to Table 3).

**Table 3:** Binding Energies (BE) (kcal/mol) and RMSD values of compounds targeting TOP2B, p38, p53, and EGFR compared to reference ligands Etoposide, Sorafenib, chromeno triazolopyrimidine, and erlotinib, respectively.

Compds	TOP2B		p38		p53		EGFR	
	BE	RMSD (Å)	BE	RMSD (Å)	BE	RMSD (Å)	BE	RMSD (Å)
4a	− 7.3	1.47	− 7.14	1.76	− 6.64	1.83	− 5.08	1.63
4d	− 7.02	1.62	− 7.05	1.94	− 7.25	1.66	− 6.55	1.31
4f.	− 6.93	1.64	− 6.81	1.99	− 6.79	1.38	− 5.50	1.71
5a	− 6.72	1.58	− 7.00	1.82	− 7.13	1.76	− 5.99	1.63
5b	− 6.54	2.12	− 6.50	1.83	− 6.73	1.96	− 6.45	1.78
Reference ligand	− 8.30	1.39	− 10.18	1.05	− 7.58	0.97	− 8.93	0.84

According to findings presented in Table 3, binding energy values for most compounds closely resemble those of the reference drug. Notably, compound **4d** demonstrated notable binding affinity with TOP2B, p38, p53, and EGFR, exhibiting binding energies of − 7.02, − 7.05, − 7.25, and − 6.55 kcal/mol, respectively.

The tabulated data in Table 4 gives details on the specific amino acids involved in the interactions of tested compounds against TOP2B, p38, p53, and EGFR proteins, and reference ligands against their corresponding specific target proteins. For every compound, the table enumerates the binding amino acids for each protein target, in consort with the type of bond formed (e.g., H-acceptor, pi-cation, etc.).

**Table 4:** Binding amino acids of tested compounds against all studied proteins (TOP2B, p38, p53, and EGFR) and reference ligands against their specific target proteins.

Compds	TOP2B	p38	p53	EGFR
	Binding Amino Acids (*Bond Type*)	Binding Amino Acids (*Bond Type*)	Binding Amino Acids (*Bond Type*)	Binding Amino Acids (*Bond Type*)
**4a**	MET-782 (*pi-H)*, GLY-871 (*pi-H)*	ARG-70 (*H-acceptor*), LEU-74 (*pi-H*)	HIS-96 (*H-pi*)	
**4d**	GLN-789 (*H-acceptor*), LYS-814 (*pi-H)*	LYS-53 (pi-cation), ASP-168 (*pi-H*)	HIS-96 (*pi-pi*)	GLY-772 (*H-acceptor*), CYS-773 (*H-acceptor*)
**4f.**	LYS-739 *(H-acceptor)*, ASN-786 *(pi-H)*	GLU-71 (*H-acceptor*), LYS-53 (*pi-cation*), ASP- 168 (*pi-H*)	HIS-96 (*pi-pi*)	
**5a**	MET-782 (*pi-H*)	ARG-70 (*H-acceptor*), HIS-148 (*H-pi*)	TYR-67 (*H-pi*)	LYS-721 (*H-acceptor*)
**5b**	LYS-814 (*pi-cation*)	ASP-168 (*pi-H*)	HIS-96 (*H-pi*)	CYS-773 (*pi-H*)
Etoposide	6-ring of DG 13 (the six-membered ring of deoxyguanosine) (*pi-H*)			
Sorafenib		MET-109 *(H-donor*), GLU-71 (*H-donor*), GLU-71 (*H-donor*), ASP-168 (*H-acceptor*), MET-109 (*H-acceptor*)		
chromeno-triazolopyrimidine			HIS-96 (*pi-pi*)	
Erlotinib				CYS-773 (*H-donor*), GLN-767 (*H-donor*), LYS-692 (*H-acceptor*), MET-769 (*H-acceptor*)

**Fig. 3:**
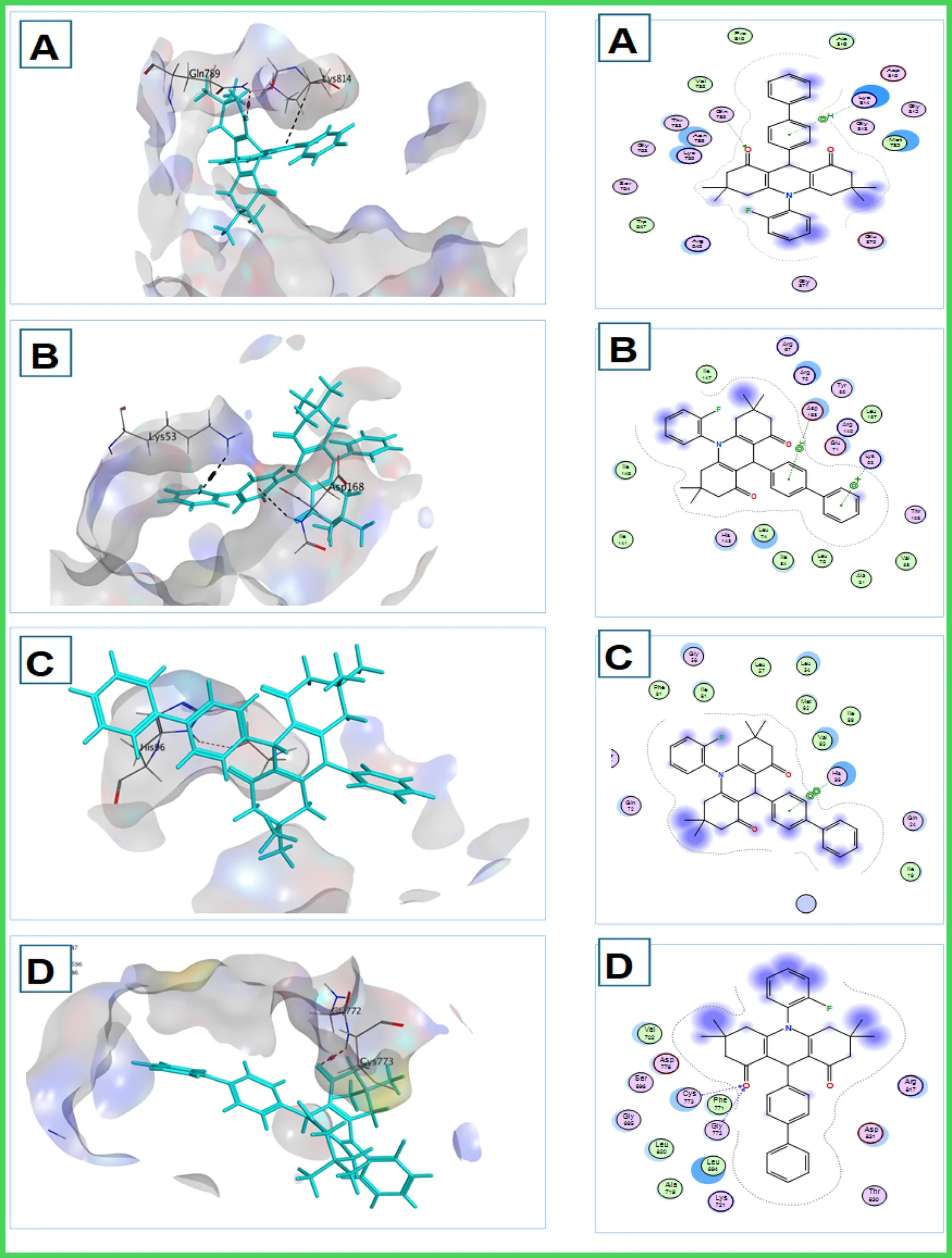
The docking outcomes of compound **4d** with proteins-TOP2B (A), p38 (B), p53 (C), and EGFR (D) are presented in the figure. In the left panels, a 3D representation illustrates the binding interaction between compound **4d** (cyan color) and the proteins (highlighted in red for hydrogen bonding and black for pi-H bonding). Conversely, the right panels showcase a 2D depiction of the binding interaction between compound **4d** and every protein.

We extended our investigation to analyze the interactions between compound **4d** and the amino acids present in 4 proteins-TOP2B, p38, p53, and EGFR operating molecular docking simulations. Our results demonstrated that compound **4d** formed stable interactions, including hydrogen bonds, pi-cation interactions, and pi-H bonds, with various key amino acids within every protein (see Fig. 3).

**Fig. 4:**
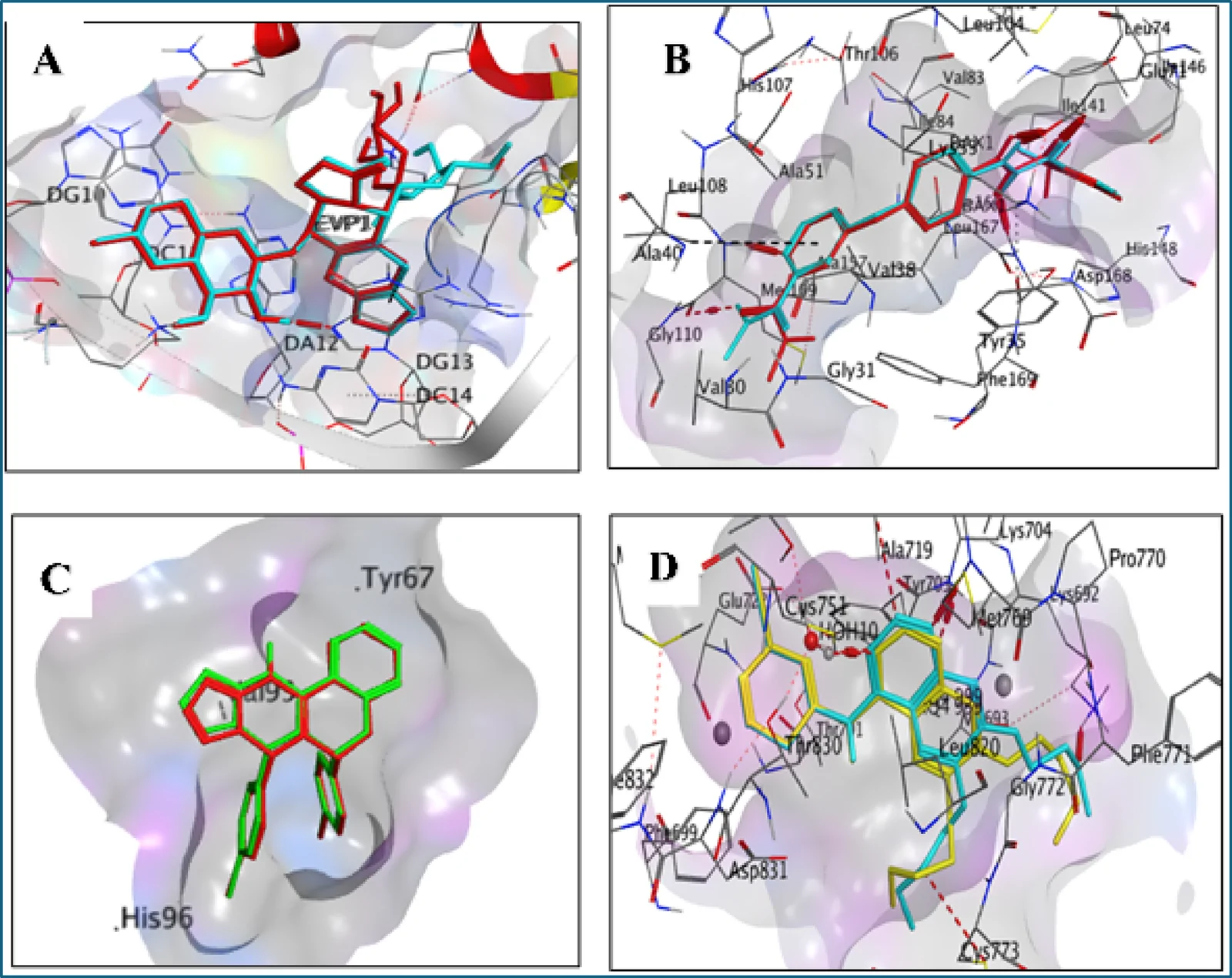
In (**A**), the 3D diagram illustrates the overlap of the native co-crystallized ligand Etoposide (cyan) with the redocked co-crystallized Etoposide (red) structure at the TOP2B protein target. The RMSD value, indicative of the disparity between the two structures, was computed to be 1.39 Å. In (**B**), the 3D diagram depicts the alignment of the native co-crystallized ligand Sorafenib (cyan) with the redocked co-crystallized Sorafenib (red) structure at the p38 protein target. The RMSD value was determined to be 1.05 Å. In (**C**), the 3D diagram shows the overlay of the native co-crystallized ligand chromeno-triazolopyrimidine (green) with the redocked co-crystallized chromeno-triazolopyrimidine (red) structure at the p53 protein target. The RMSD value was calculated to be 0.97 Å. Lastly, in (**D**), the 3D diagram exhibits the alignment of the native co-crystallized ligand erlotinib (cyan) with the redocked co-crystallized erlotinib (yellow) structure at the EGFR protein target. The RMSD value was determined to be 0.84 Å.

The validation of docking was ensured by comparing the native co-crystallized ligands with their relevant protein targets against the redocked co-crystallized ligands. This comparison was envisioned through 3D diagrams, enabling the calculation of RMSD values for the superimpositions. The outcomes, depicted in graphical form (Fig. 4), provided insight into the accuracy of the docking simulations.

### Molecular dynamics (MD) simulations

**Fig. 5:**
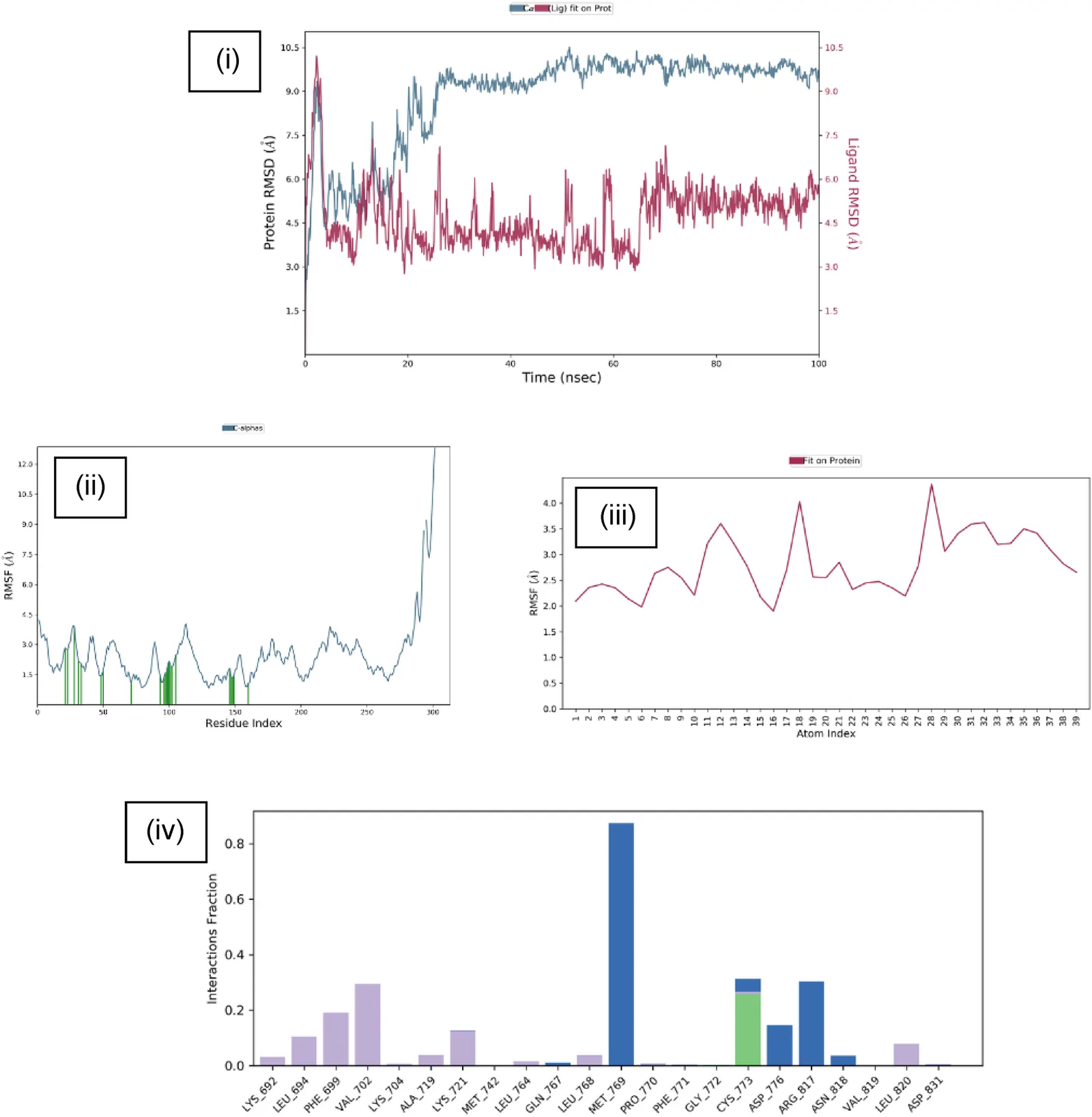
(i) RMSD analysis of EGFR bound to compound **4d** over a 100-ns MD simulation. (ii) RMSF analysis comparing the stability and flexibility of EGFR when bound to compound **4d** with low fluctuations, suggesting a stable conformation with minimal localized flexibility. (iii) RMSF analysis shows that compound **4d** has low fluctuations, maintaining a stable conformation. Overall, compound **4d** induces a more stable binding conformation in EGFR. (iv) MD Histogram of protein–ligand interactions for compound **4d**, illustrating the interaction fractions of different types of molecular interactions between the protein EGFR and ligand (compound **4d**). The types of interactions are categorized into hydrogen bonds (H-bonds, green), hydrophobic interactions (purple), ionic interactions (pink), and water bridges (blue). The x-axis represents specific amino acid residues involved in the interactions, while the y-axis shows the fraction of interactions observed during the MD simulation.

However molecular docking is a valuable technique for predicting the binding affinities of ligands to proteins, it is limited by its static representation of molecular interactions. While docking effectively predicts binding modes, it may not fully account for the dynamic nature of ligand–protein interactions influenced by factors such as solvent effects, conformational flexibility, and entropy. To address these limitations, MD simulations are crucial, as they provide a detailed and time-resolved perspective on these interactions, offering a more comprehensive understanding. In this study, we selected compound **4d**, the most potent compound, as it demonstrated the highest binding affinity with EGFR protein receptors (related to DNA transcription and cell division). MD simulations demonstrated that compound **4d** maintains a stable binding profile with EGFR, supporting prior molecular docking and bioassay findings that indicated its superior potency as an EGFR inhibitor. Over the 100 ns simulation, RMSD of the EGFR-compound **4d** complex stabilized more quickly and showed minimal fluctuations (around 1.31 Å), which likely accounts for its enhanced inhibitory activity observed in bioassays (Fig. 5i).

This graph provides evidence of the stability of the protein–ligand complex during the MD simulation, with both the protein and ligand showing stabilization, albeit at different RMSD ranges. The root-mean square fluctuation (RMSF) analysis revealed that the binding of compound **4d** resulted in reduced conformational flexibility in critical regions of EGFR, especially around the active site. The lower RMSF values for the residues surrounding the binding pocket in the compound **4d** complex suggest that this ligand stabilizes the enzyme in a more rigid and catalytically less active conformation, which may account for its improved inhibitory effects (Fig. 5ii and iii). Although the RMSF profile shows small local fluctuations along the protein backbone, most interacting residues remained within a fluctuation range below ~ 3 Å during the simulation. This behavior generally indicates acceptable structural stability of the protein–ligand complex. Moreover, the ligand itself displayed relatively limited atomic fluctuations, suggesting that compound **4d** maintained a consistent orientation within the binding pocket throughout the simulation. Taken together, these observations support the overall stability of the EGFR–compound **4d** complex during the MD trajectory (Fig. 5ii and iii).

MD simulations of compound **4d** with EGFR revealed significant interactions with key residues within the enzyme’s active site. The protein–ligand contacts histogram (Fig. 5iv) indicates that the key amino acids MET 769, VAL 702, CYS 773, and ARG 817 were the most interactive residues with compound **4d**. These interactions primarily involved hydrogen bonding, water-mediated bridges, and hydrophobic interactions, respectively, and were consistently maintained throughout the simulation. Such interactions are critical for stabilizing the ligand within the binding pocket, ensuring its proper orientation and contributing to the high binding affinity observed for compound **4d**.

### Comparative evaluation and lead prioritization

To systematically identify the most promising candidate among the evaluated derivatives, we performed a comparative assessment integrating cytotoxicity, selectivity, docking affinity, and molecular dynamics stability.

Specifically, we compared the compounds across four key criteria:

Potency (IC₅₀): Compound **4d** showed the lowest IC₅₀ against H460 lung cancer cells (17.91 ± 2.8 µg/mL), outperforming the other derivatives across most cell lines.

Selectivity (SI): Compound **4d** exhibited the highest selectivity index toward H460 cells (SI = 20.7), indicating a significantly wider therapeutic window compared to compounds **4a** (SI = 5.5), 5b (SI = 6.1), and others.

Binding Affinity (Docking): In molecular docking studies, **4d** demonstrated consistently strong binding energies across all four target proteins (TOP2B: − 7.02; p38: − 7.05; p53: − 7.25; EGFR: − 6.55 kcal/mol), with the most favorable pose stability (lowest RMSD = 1.31 Å for EGFR).

Based on this multi-parameter analysis, compound** 4d** emerges as the most promising candidate in this series. Its combination of high potency, exceptional selectivity, and robust multi-target binding supports its advancement as a lead scaffold for further structural optimization and preclinical evaluation. The comparative data integrated into Table (1) and Table (2), in terms of IC_50_ and SI values, respectively, demonstrated that our lead compound **4d** show superior tumor cells selectivity and lower toxicity to healthy cells relative to the standard drugs. Such notice can be strongly correlated with the previously mentioned fact that telomerase activity is widely-expressed/re-activated in more than 85% of the neoplastic cells, while being almost undetectable in normal ones. Hence so, the compounds under investigation in this research may be considered to selectively target neoplastic cells and concomitantly sparing the normal healthy cells the common undesired off-target effects of anti-neoplastic therapeutics.

## Conclusion

Employing a one-pot multicomponent reaction using PTSA catalyst, this work reported the efficient synthesis of hexahydro-1,8-acridinedione derivatives through structural modifications at positions-9 and 10 of the acridinedione scaffold, aimed to enhance their antitumor potency and selectivity. Compound **4d** emerged as the most potent derivative in this series, demonstrating an IC₅₀ of 17.91 ± 2.8 µg/mL against H460 lung cancer cells, comparable to literature-reported EGFR inhibitors. Compound **4d** exhibits superior selectivity toward cancer cells (SI = 20.7). It also inhibited telomerase activity in A549 cancer cell line. Its efficacy is linked to its unique structural features and interactions with key proteins involved in cancer cell growth and survival. The originality of the present study lies in exploring novel structural modifications to the 1,8-acridinedione framework, specifically the introduction of N-arylated substituents and an additional aryl group at position-9, which are hypothesized to enhance DNA-binding affinity and specificity. These innovative structural variations represent a significant advancement in the design of 1,8-acridinedione derivatives. Future perspectives include in vivo efficacy studies and pharmacokinetic profiling to translate these promising in vitro findings into therapeutic applications.

## Materials and methods

### Chemistry

Melting points were determined using the Stuart® SMP50 Automatic Digital Melting Point Apparatus. Infrared (IR) spectra were recorded utilizing potassium bromide (KBr) disks on a Fourier transform infrared Thermo Electron Nicolet 7600 spectrometer (Thermo Fisher Scientific Inc., Waltham, MA, USA) at the Central Laboratory of the Faculty of Science, Ain Shams University. The ^1^H NMR, ^13^C APT NMR, and ^19^F NMR spectra were recorded using a Bruker 400 MHz Avance III Nuclear Magnetic Resonance (NMR) spectrometer at 400, 100, and 376 MHz, respectively using tetramethyl silane (TMS) as an internal standard in deuterated dimethyl sulfoxide (DMSO-*d*_6_) at the Faculty of Pharmacy, Mansoura University. Mass spectra were recorded on a Shimadzu Liquid Chromatograph Mass Spectrometer LCMS-2020 at Egypt-Japan University for Science and Technology. Reaction progress was monitored via Thin Layer Chromatography (TLC) using Merck Kiesel Gel 60 F_254_ analytical sheets sourced from Fluka. Antitumor activity assessments were conducted at the Protein Research Department, GEBRI, City of Scientific Research and Technological Applications (SRTACity), New Borg Al-Arab City, 21934, Alexandria, Egypt.

### 9-([1,1′-Biphenyl]-4-yl)-10-(aryl)-3,3,6,6-tetramethyl-3,4,6,7,9,10-hexahydroacridine-1,8(2H,5H)-dione (4a-4 g)

A solution of dimedone (**1**) (2 mmol), different aromatic amines **2** (1 mmol), biphenyl-4-carbaldehyde **3a** (1 mmol, 0.182 g) and PTSA (0.05 g) in DMF (0.5 mL) were stirred in a 10 mL round-bottomed flask at 90 °C for 4 h (TLC). After the reaction completion, the solid that deposited on cooling was filtered and recrystallized from methanol to get pure compounds.

### 9-([1,1′-Biphenyl]-4-yl)-10-(4-chloro-3-(trifluoromethyl)phenyl)-3,3,6,6-tetramethyl-3,4,6,7,9,10-hexahydroacridine-1,8(2H,5H)-dione (4a)

White powder, (0.5 g, 83% yield); mp. 252–254 °C. IR (KBr) *ν*_max_ (cm^−1^): 3066, 3030 (=C–H, sp^2^); 2959, 2871 (C–H, sp^3^); 1643 (C=O acridinedione); 1578, 1481 (C=C), 1311 (C–N stretching). ^1^H NMR (400 MHz, DMSO-*d*_6_) *δ* (ppm):): 0.73 (s, 6 H, 2 CH_3_), 0.88 (s, 6 H, 2 CH_3_), 1.76 (d, 2 H, CH_2_, *J* = *7.5 Hz*), 1.99 (d, 2 H, CH_2_, *J* = 7.2 Hz), 2.17 (d, 2 H, CH_2_, J = 7.9 Hz), 2.21 (d, 2 H, CH_2_, J = 7.0 Hz), 5.04 (s, 1 H, acridinedione-H_9_), 7.30–7.61 (m, 9 H, Ar–H, biphenyl-H), 7.79–7.96 (m, 3 H, Ar–H). ^19^F NMR (376 MHz, DMSO-*d*_6_): -61.24. MS (ESI^+^) *m/z*: Expected for C_36_H_33_ClF_3_NO_2_ [M + H]^+^: 604.11, found 604.

### 9-([1,1′-Biphenyl]-4-yl)-10-(2,4-dichlorophenyl)-3,3,6,6-tetramethyl-3,4,6,7,9,10-hexahydroacridine-1,8(2H,5H)-dione (4b)

White powder, (0.45 g, 79% yield); mp. 240–242 °C. IR (KBr) *ν*_max_ (cm^−1^): 3066, 3027 (=C–H, sp^2^); 2959, 2871 (C–H, sp^3^); 1640 (C=O acridinedione); 1579, 1480 (C=C); 1304 (C–N stretching). ^1^H NMR (400 MHz, DMSO-*d*_6_) *δ* (ppm):): 0.78 (s, 6 H, 2 CH_3_), 0.90 (s, 6 H, 2 CH_3_), 1.55 (d, 2 H, CH_2_, J = 7.0 Hz), 1.93 (d, 2 H, CH_2_, J = 6.9 Hz), 1.99 (d, 2 H, CH_2_, J = 7.4 Hz), 2.19 (d, 2H, CH_2_, J = 7.1 Hz), 2.23 (d, 2 H, CH_2_, J = 7.3 Hz), 5.04 (s, 1 H, acridinedione-H_9_), 7.30–7.51 (m, 11 H, Ar–H), 8.04 (s, 1 H, Ar-H_15_). MS (ESI +) *m/z*: Expected for C_35_H_33_Cl_2_NO_2_ [M + H]^+^: 570.19, found 570.

### 9-([1,1′-Biphenyl]-4-yl)-3,3,6,6-tetramethyl-10-(o-tolyl)-3,4,6,7,9,10-hexahydroacridine-1,8(2H,5H)-dione (4c)

White powder, (0.40 g, 77% yield); mp. 222–224 °C. IR (KBr) *ν*_max_ (cm^−1^): 3056, 3030 (=C–H, sp^2^); 2959, 2928, 2867 (C–H, sp^3^); 1654, 1638 (C=O acridinedione); 1576, 1487 (C=C); 1302 (C–N stretching). ^1^H NMR (400 MHz, DMSO-*d*_6_) *δ* (ppm):): 0.79 (s, 6 H, 2 CH_3_), 0.87 (s, 6 H, 2 CH_3_), 1.53 (d, 2 H, CH_2_, J = 7.5 Hz), 2.01 (d, 2 H, CH_2_, J = 7.9 Hz), 2.19 (d, 2 H, CH_2_, J = 7.8 Hz), 2.23 (d, 2 H, CH_2_, J = 7.0 Hz), 2.30 (s, 3 H, aryl-CH_3_), 5.13 (s, 1 H, acridinedione-H_9_), 7.31–7.65 (m, 13 H, Ar–H). ^13^C NMR (100 MHz, DMSO-*d*_6_) *δ* (ppm): 195.63 (2 C=O, acridinedione), 150.58, 146.23, 140.34, 137.91, 137.82, 136.79, 131.83, 130.55, 130.18, 129.33, 129.01, 128.03, 127.61, 126.91, 126.41, (Ar–C), 113.08, 49.94, 41.60, 32.58, 32.21, 30.08 (2 CH_3_), 26.19 (2 CH_3_), 18.31. MS (ESI +) *m/z*: Expected for C_36_H_37_NO_2_ [M + H]^+^: 517.28, found 517.

### 9-([1,1′-Biphenyl]-4-yl)-10-(2-fluorophenyl)-3,3,6,6-tetramethyl-3,4,6,7,9,10-hexahydroacridine-1,8(2H,5H)-dione (4d)

White powder, (0.45 g, 86% yield); mp. 240–242 °C. IR (KBr) *ν*_max_ (cm^−1^): 3073, 3022 (=C–H, sp^2^); 2956, 2867, 2817 (C–H, sp^3^); 1637 (C=O); 1579, 1499, 1458 (C=C); 1303 (C–N stretching). ^1^H NMR (400 MHz, DMSO-*d*_6_) *δ* (ppm):): 0.78 (s, 6H, 2 CH_3_), 0.90 (s, 6H, 2 CH_3_), 1.69 (d, 2H, CH_2_, J = 7.2 Hz), 1.92 (d, 2H, CH_2_, J = 7.5 Hz), 2.02 (d, 2H, CH_2_, J = 7.1 Hz), 2.21 (d, 2H, CH_2_, J = 7.9 Hz), 2.28 (d, 2H, CH_2_, J = 7.6 Hz), 5.07 (s, 1H, acridinedione-H_9_), 7.32–7.65 (m, 13H, Ar–H). ^13^C NMR (100 MHz, DMSO-*d*_6_) *δ* (ppm): 195.63 (2 C=O), 160.24, 157.79, 150.26, 146.09, 145.75, 140.49, 140.41, 138.17, 138.08, 129.32, 128.97, 128.78, 126.92, 126.76, 126.45, (Ar–C), 113.82, 49.96, 41.13, 32.58, 32.27, 29.84 (2 CH_3_), 27.04 (2 CH_3_). ^19^F NMR (376 MHz, DMSO-*d*_6_): − 120.12, − 122.10. MS (ESI +) *m/z*: Expected for C_35_H_34_FNO_2_ [M + H]^+^: 520.26, found 520.

### 9-([1,1′-Biphenyl]-4-yl)-3,3,6,6-tetramethyl-10-(4-nitrophenyl)-3,4,6,7,9,10-hexahydroacridine-1,8(2H,5H)-dione (4e)

White powder, (0.4 g, 73% yield); mp. 281–283 °C. IR (KBr) *ν*_max_ (cm^−1^): 3059, 3029 (=C–H, sp^2^); 2959, 2871 (C–H, sp^3^); 1641 (C=O acridinedione); 1590, 1527, 1487, 1448 (C=C); 1300 (C–N stretching). ^1^H NMR (400 MHz, DMSO-*d*_6_) *δ* (ppm): ): 0.76 (s, 6H, 2 CH_3_), 0.90 (s, 6H, 2 CH_3_), 1.81 (d, 2H, CH_2_, J = 7.7 Hz), 2.04 (d, 2H, CH_2_, J = 7.3 Hz), 2.21 (d, 2H, CH_2_, J = 7.9 Hz), 2.25 (d, 2H, CH_2_, J = 6.5 Hz), 5.11 (s, 1H, acridinedione-H_9_), 7.32–7.65 (t, 1H, Ar–H, biphenyl-H), 7.43–7.46 (m, 4H, Ar–H, biphenyl-H), 7.57–7.59 (d, 2H, Ar–H, biphenyl-H, J = 8.2 Hz), 7.63–7.64 (d, 2H, Ar–H, biphenyl-H, J = 8.1 Hz), 7.78–7.80 (d, 2H, Ar–H, nitrophenylene-H, J = 8.3 Hz), 8.46–8.48 (d, 2H, Ar–H, nitrophenylene-H, J = 8.1 Hz). ^13^C NMR (100 MHz, DMSO-*d*_6_) *δ* (ppm): 195.69 (2 C=O), 150.14, 148.12, 145.74, 144.60, 140.46, 138.15, 132.04, 129.38, 128.76, 127.64, 126.91, 126.74, 125.78 (Ar–C), 113.67, 50.07, 41.35, 32.62, 32.30, 29.61 (2 CH_3_), 26.66 (2 CH_3_). MS (ESI +) *m/z*: Expected for C_35_H_34_N_2_O_4_ [M + H]^+^: 548.25, found 548.

### 9-([1,1′-Biphenyl]-4-yl)-10-(4-fluorophenyl)-3,3,6,6-tetramethyl-3,4,6,7,9,10-hexahydroacridine-1,8(2H,5H)-dione (4f.)

White powder, (0.45 g, 86% yield); mp. 265–267 °C. IR (KBr) *ν*_max_ (cm^−1^): 3076, 3019 (=C–H, sp^2^); 2955, 2871, 2821 (C–H, sp^3^); 1636 (C=O acridinedione); 1578, 1509, 1481, 1448 (C=C) ;1301 (C–N stretching). ^1^H NMR (400 MHz, DMSO-*d*_6_) *δ* (ppm):): 0.76 (s, 6H, 2 CH_3_), 0.91 (s, 6H, 2 CH_3_), 1.79 (d, 2H, CH_2_, J = 7.4 Hz), 2.04 (d, 2H, CH_2_, J = 7.7 Hz), 2.19 (d, 2H, CH_2_, J = 7.1 Hz), 2.23 (d, 2H, CH_2_, J = 7.5 Hz), 5.10 (s, 1H, acridinedione-H_9_), 7.31–7.64 (m, 13H, Ar–H). ^13^C NMR (100 MHz, DMSO-*d*_6_) *δ* (ppm): 195.64 (2 C=O), 163.56, 161.10, 150.94, 145.97, 140.48, 138.05, 135.20, 129.36, 128.70, 127.61, 126.90, 126.71 (Ar–C), 113.41, 50.02, 41.41, 32.47, 32.24, 29.71 (2 CH_3_), 26.68 (2 CH_3_). ^19^F NMR (376 MHz, DMSO-*d*_6_): − 111.64. MS (ESI +) m/z: Expected for C_35_H_34_FNO_2_ [M + H]^+^: 521.26, found 521.

### 9-([1,1′-Biphenyl]-4-yl)-10-(2-chlorophenyl)-3,3,6,6-tetramethyl-3,4,6,7,9,10-hexahydroacridine-1,8(2H,5H)-dione (4 g)

White powder, (0.42 g, 78% yield); mp. 256–258 °C. IR (KBr) *ν*_max_ (cm^−1^): 3060, 3027 (=C–H, sp^2^); 2960, 2929, 2868 (C–H, sp^3^); 1641 (C=O acridinedione); 1580, 1517, 1478, 1449 (C=C); 1305 (C–N stretching). ^1^H NMR (400 MHz, DMSO-*d*_6_) *δ* (ppm): ): 0.74 (s, 6H, 2 CH_3_), 0.86 (s, 6H, 2 CH_3_), 1.48 (d, 2H, CH_2_, J = 7.1 Hz), 1.96 (d, 2 H, CH_2_, J = 6.9 Hz), 2.20 (d, 2H, CH_2_, J = 7.3 Hz), 2.24 (d, 2H, CH_2_, J = 7.1 Hz), 5.02 (s, 1H, acridinedione-H_9_), 7.29–7.33 (t, 1H, Ar–H), 7.40–7.44 (t, 2H, Ar–H, J = 7.5 Hz), 7.53–7.63 (m, 9H, Ar–H), 7.79–7.80 (d, 1H, Ar–H, J = 8.2 Hz). ^13^C NMR (100 MHz, DMSO-*d*_6_) *δ* (ppm): 195.66 (2 C=O), 150.26, 146.09, 145.75, 140.41, 138.08, 132.53, 129.32, 128.78, 126.92, 126.76, 126.45 (Ar–C), 113.82, 49.96, 41.13, 32.73, 32.27, 29.18 (2 CH_3_), 26.40 (2 CH_3_). MS (ESI +) *m/z*: Expected for C_35_H_34_ClNO_2_ [M + H]^+^: 536.23, found 536.

### 9-(4-Bromophenyl)-10-(aryl)-3,3,6,6-tetramethyl-3,4,6,7,9,10-hexahydroacridine-1,8(2H,5H)-dione (5a-e)

A mixture of dimedone **1** (2 mmol), different aromatic amines **2** (1 mmol), 4-bromobenzaldehyde **3b** (1 mmol, 0.185 g), and PTSA (0.05 g) in DMF (0.5 mL) stirred at 90 °C for 4 h (TLC). After the reaction completion, the solid that separated out on cooling was filtered and washed with hot methanol to get pure compounds.

### 9-(4-Bromophenyl)-3,3,6,6-tetramethyl-10-(2-(piperidin-1-yl)ethyl)-3,4,6,7,9,10-hexahydroacridine-1,8(2H,5H)-dione (5a)

White powder, (0.45 g, 83% yield); mp. 202–204 °C. IR (KBr) *ν*_max_ (cm^−1^): 3028 (=C–H, sp^2^); 2951, 2934, 2903, 2865 (C–H, sp^3^); 1644 (C=O acridinedione); 1559, 1483, 1470, 1449 (C=C); 1300 (C–N stretching). ^1^H NMR (400 MHz, DMSO-*d*_6_) *δ* (ppm): ): 0.90 (s, 6H, 2 CH_3_), 1.02 (s, 6H, 2 CH_3_), 1.40–1.51 (m, 6H, 3 CH_2_, piperidine-H), 2.05 (d, 2H, CH_2_, J = 7.5 Hz), 2.17 (d, 2H, CH_2_, J = 7.1 Hz), 2.39–2.40 (m, 6H, 3 CH_2_), 2.56–2.66 (m, 4H, 2 CH_2_), 3.83 (s, 2H, CH_2_), 4.97 (s, 1H, acridinedione-H_9_), 7.08–7.11 (d, 2H, Ar–H, J = 7.0 Hz), 7.31–7.33 (d, 2H, Ar–H, J = 8.0 Hz). MS (ESI +) *m/z*: Expected for C_30_H_39_BrN_2_O_2_ [M + H]^+^: 541.22, found 541.

### 9-(4-Bromophenyl)-10-(4-chloro-3-(trifluoromethyl)phenyl)-3,3,6,6-tetramethyl-3,4,6,7,9,10-hexahydroacridine-1,8(2H,5H)-dione (5b)

White powder, (0.48 g, 79% yield); mp. 302–304 °C. IR (KBr) *ν*_max_ (cm^−1^): 3064, 3040 (=C–H, sp^2^); 2951, 2934, 2903, 2865 (C–H, sp^3^); 1642 (C=O acridinedione); 1579, 1482, 1421 (C=C) ; 1300 (C–N stretching). ^1^H NMR (400 MHz, DMSO-*d*_6_) *δ* (ppm):): 0.72 (s, 6H, 2 CH_3_), 0.88 (s, 6H, 2 CH_3_), 1.75 (d, 2H, CH_2_, J = 7.0 Hz), 1.98 (d, 2H, CH_2_, J = 7.1 Hz), 2.16 (d, 2H, CH_2_, J = 7.4 Hz), 2.20 (d, 2H, CH_2_, J = 7.2 Hz), 4.97 (s, 1H, acridinedione-H_9_), 7.27 (d, 2H, Ar–H, bromophenylene-H, J = 8.1 Hz), 7.41 (d, 2H, Ar–H, bromophenylene-H, J = 8.2 Hz), 7.81–7.97 (m, 3H, Ar–H). ^19^F NMR (376 MHz, DMSO-*d*_6_): − 61.19. MS (ESI +) *m/z*: Expected for C_30_H_28_BrClF_3_NO_2_ [M + H]^+^: 608.09, found 608.

### 9-(4-Bromophenyl)-10-(2,4-dichlorophenyl)-3,3,6,6-tetramethyl-3,4,6,7,9,10-hexahydroacridine-1,8(2H,5H)-dione (5c)

White powder, (0.4 g, 70% yield); mp. 313–315 °C. IR (KBr) *ν*_max_ (cm^−1^): 3066, 3024 (=C–H, sp^2^); 2956, 2933, 2868 (C-H, sp^3^); 1649 (C=O acridinedione); 1580, 1477 (C=C); 1304 (C–N stretching). ^1^H NMR (400 MHz, DMSO-*d*_6_) *δ* (ppm):): 0.75 (s, 6H, 2 CH_3_), 0.91 (s, 6H, 2 CH_3_), 1.53 (d, 2H, CH_2_, J = 7.1 Hz), 1.95 (d, 2H, CH_2_, J = 7.2 Hz), 2.18 (d, 2H, CH_2_, J = 7.5 Hz), 2.23 (d, 2H, CH_2_, J = 7.7 Hz), 4.96 (s, 1H, acridinedione-H_9_), 7.27–7.46 (m, 4H, Ar–H), 7.70–7.74 (m, 2H, Ar–H), 8.03 (s, 1H, Ar–H). MS (ESI +) *m/z*: Expected for C_29_H_28_BrCl_2_NO_2_ [M + H]^+^: 574.07, found 574.

### 9-(4-Bromophenyl)-3,3,6,6-tetramethyl-10-(o-tolyl)-3,4,6,7,9,10-hexahydroacridine-1,8(2H,5H)-dione (5d)

White powder, (0.43 g, 83% yield); mp. 253–255 °C. IR (KBr) *ν*_max_ (cm^−1^): 3051, 3026 (=C–H, sp^2^); 2951, 2873 (C–H, sp^3^); 1659, 1641 (C=O acridinedione); 1577, 1486, 1468 (C=C); 1300 (C–N stretching). ^1^H NMR (400 MHz, DMSO-*d*_6_) *δ* (ppm):): 0.72 (s, 6H, 2 CH_3_), 0.83 (s, 6H, 2 CH_3_), 1.46 (d, 2H, CH_2_, J = 7.5 Hz), 2.00 (d, 2H, CH_2_, J = 7.3 Hz), 2.20 (d, 2H, CH_2_, J = 7.8 Hz), 2.28 (d, 2H, CH_2_, J = 7.4 Hz), 2.21 (s, 3H, CH_3_), 5.00 (s, 1H, acridinedione-H_9_), 7.10 (d, 2H, Ar–H, J = 8.2 Hz), 7.24 (m, 6H, Ar–H). MS (ESI +) *m/z*: Expected for C_30_H_32_BrNO_2_ [M + H]^+^: 520.16, found 520.

### 9-(4-Bromophenyl)-10-(2-chlorophenyl)-3,3,6,6-tetramethyl-3,4,6,7,9,10-hexahydroacridine-1,8(2H,5H)-dione (5e)

White powder, (0.45 g, 84% yield); mp. 281–283 °C. IR (KBr) *ν*_max_ (cm^−1^): 3061, 3027 (=C–H, sp^2^); 2955, 2930, 2909, 2879 (C–H, sp^3^); 1646 (C=O acridinedione); 1580, 1480 (C=C); 1302 (C–N stretching). ^1^H NMR (400 MHz, DMSO-*d*_6_) *δ* (ppm):): 0.74 (s, 6H, 2 CH_3_), 0.86 (s, 6H, 2 CH_3_), 1.46 (d, 2H, CH_2_, J = 7.6 Hz), 1.96 (d, 2H, CH_2_, J = 7.3 Hz), 2.04 (d, 2H, CH_2_, J = 7.1 Hz), 2.22 (d, 2H, CH_2_, J = 7.5 Hz), 5.00 (s, 1 H, acridinedione-H_9_), 7.27–7.81 (m, 8H, Ar–H). MS (ESI +) *m/z*: Expected for C_29_H_29_BrClNO_2_ [M + H]^+^: 540.11, found 540.

### In vitro cytotoxicity assay

The cell lines were obtained from American Type Culture Collection (ATCC). Using the Thiazolyl Blue Tetrazolium Bromide (MTT) method^11^. The cytotoxicity of various concentrations of compounds (**5a, 5b, 4a, 4d**, and **4f.**) was assessed against the HSF, H460, A431, A549, and MDA-MB-231 cell lines. Cells (10 × 10^3^) were cultured on a 96-well plate overnight at 37 °C, 5% CO_2_, and 88% humidity. The total volume of DMEM supplemented medium and the synthesized compounds supernatant used was 200 µL, with final concentrations of 25, 50, 100, 150, and 200 µg/mL. The plate was then incubated at 37 °C, 5% CO_2_ for 48 h. After incubation, debris and dead cells were removed by washing three times with fresh medium. MTT solution (5 mg/mL in PBS buffer, 20 µL) was added to each well and shaken for 5 min. at 150 rpm to ensure thorough mixing. The cells were further incubated at 37 °C and 5% CO_2_ for 3–5 h to allow for MTT metabolism by viable cells. Next, 200 µL of DMSO was added to each well and shaken for an additional 5 min. at 150 rpm. The cell viability was then determined by measuring the optical density at 630 nm after subtracting the optical density at 570 nm.

The relative cell viability (%) was calculated using the formula: [ODS / ODC] × 100, where ODS is the mean optical density of the sample and ODC is the mean optical density of the control group. The results were displayed as viability (%) as a function of concentrations using GraphPad Prism software version 8. The tumor-selectivity index (SI value) was determined by dividing the mean IC_50_ against normal cells by the mean IC_50_ against tumor cells. Additionally, the effect of the most potent antitumor compound (**4d**) on the morphology of the tested cancer cells at their IC_50_ values was visualized under phase-contrast microscopy (Olympus, Germany) and compared with untreated (control) cells.

IC_50_ values, representing the concentration inhibiting 50% of cell growth, were calculated using non-linear regression analysis of dose–response curves generated from MTT assay data using GraphPad Prism software (version 8). Experiments were conducted in triplicate (n = 3), and results are expressed as mean ± standard error of the mean (SEM). Statistical significance was determined using one-way ANOVA followed by Tukey’s post-hoc test, with *p*-values < 0.05 considered significant”. This addition clarifies the methodology and ensures transparency in data analysis.

### Telomerase inhibition assay

The potential anti-hTERT activity of the derivative compound **4d** was studied on A549 lung cancerous cells as per a quantitative telomeric repeat amplification protocol (Q-TRAP). Practically, A549 cells were seeded in a 6-well plate at a density of 3.5 ⅹ 10^5^ cells/well, then treated for 72 h with three different concentrations of the derivative compound **4d**, representing; ½IC_50_ (14.51 μg/mL), IC_50_ (29.01 μg/mL) and 2IC_50_ (58.02 μg/mL) values of the mentioned derivative compound against the mentioned cell line. After which, the cells were lysed and an equal protein amount from each cell lysate were used as a component of a usual qPCR reaction mixture that was initially incubated at 30 °C for 30 min. as optimum for hTERT-mediated telomeric ends extension followed by a standard qPCR reaction to detect the telomerase activity products and accordingly calculating relative telomerase activity (RTA)^68^. Xpert Fast SYBR Green qPCR Master Mix (GE20.5100) was used for running qPCR reactions in addition to bicinchoninic acid (BCA) protein assay kit (A65453) to quantify protein concentration in cell lysates.

### Docking studies

The Autodock vina software was utilized to simulate the binding orientations and interactions of promising anticancer compounds (**5a, 5b, 4a, 4d**, and **4f.**) with four tumor-regulating proteins: TOP2B, p38, p53, and EGFR. The 3D structures of the selected proteins were identified by their respective PDB IDs: 3QX3 (TOP2B), 3HEG (p38), 3JZK (p53), and 1M17 (EGFR). Water molecules and repeating chains were removed, and protons were added to the proteins. The energy of the proteins was minimized, and the ligand pocket was isolated. The validity of the downloaded structures was confirmed by re-docking the downloaded ligands into the isolated ligand pocket, resulting in a root mean square deviation (RMSD) of less than 1.5. The anticancer compounds were prepared for docking using Autodock vina chemical structure creation process, followed by the addition of protons to 3D structures. Energy minimization was further conducted using the Force Field MMFF94x. The structures of the prepared compounds were then added to the built database, and docking simulations were performed to determine the binding energies and mechanisms of binding for the recently synthesized compounds^16^.

### Molecular dynamic simulations

Molecular dynamics (MD) simulations for both compound **4d** in complex with p53 was performed using the non-commercial version 2022.1 of Desmond_Maestro, a high-performance MD simulation package by Schrödinger, Inc. The simulations began with the top-ranked poses from prior molecular docking studies, ensuring that the starting conformations closely represented the biologically relevant binding modes. The protein–ligand complexes were solvated in an orthorhombic box of TIP3P water molecules, with a 10 Å buffer extending in all directions around the protein. Counter ions (Na⁺ and Cl⁻) were added to neutralize the system, achieving a physiological ionic strength of 0.15 M. The OPLS_2005 force field was applied to describe the protein, ligands, and solvent. Energy minimization was carried out using the steepest descent algorithm until the gradient threshold of 25 kcal/mol/Å was met. The equilibration phase was performed in the NPT ensemble (constant number of particles, pressure, and temperature) at 300 K and 1 atm, using the Berendsen thermostat and barostat. Temperature and pressure were maintained with coupling constants of 1.0 ps and 2.0 ps, respectively. The production phase consisted of a 100 ns MD simulation for each complex, with a 2 fs time step. System coordinates were recorded every 1.2 ps for subsequent analysis. Post-simulation analyses included RMSD, RMSF, and secondary structure evaluations to assess the stability and conformational behavior of the protein–ligand complexes. Protein–ligand interactions were examined using the Simulation Interactions Diagram module in Maestro, categorizing them into hydrogen bonds, hydrophobic interactions, ionic interactions, and water bridges. The binding free energy of each ligand was estimated using the MM-GBSA method to quantify their relative binding affinities.

### Statistical analysis

The experiments were carried out in triplicate (n = 3), and the results are expressed as mean ± standard error of the mean (SEM). Statistical significance of the data was determined using one-way analysis of variance (ANOVA), followed by Tukey's post-hoc test for multiple comparisons. Graph Pad Prism software version 8 was utilized for statistical analysis. Differences between groups were considered statistically significant at *p*-values < 0.05.

## Supplementary Information

Below is the link to the electronic supplementary material.

Supplementary Material 1

Supplementary Material 2

## Data Availability

All data generated or analyzed during this study are included in this published article and its supplementary information files.
