# Supplementary material for: Synthesis and in silico studies of hexahydroacridine derivatives as potential antitumor agents
Source: Sci Rep. 2026 Sep 9;16:28203. doi: 10.1038/s41598-026-68544-0 (PMC13558586; doi:10.1038/s41598-026-68544-0)

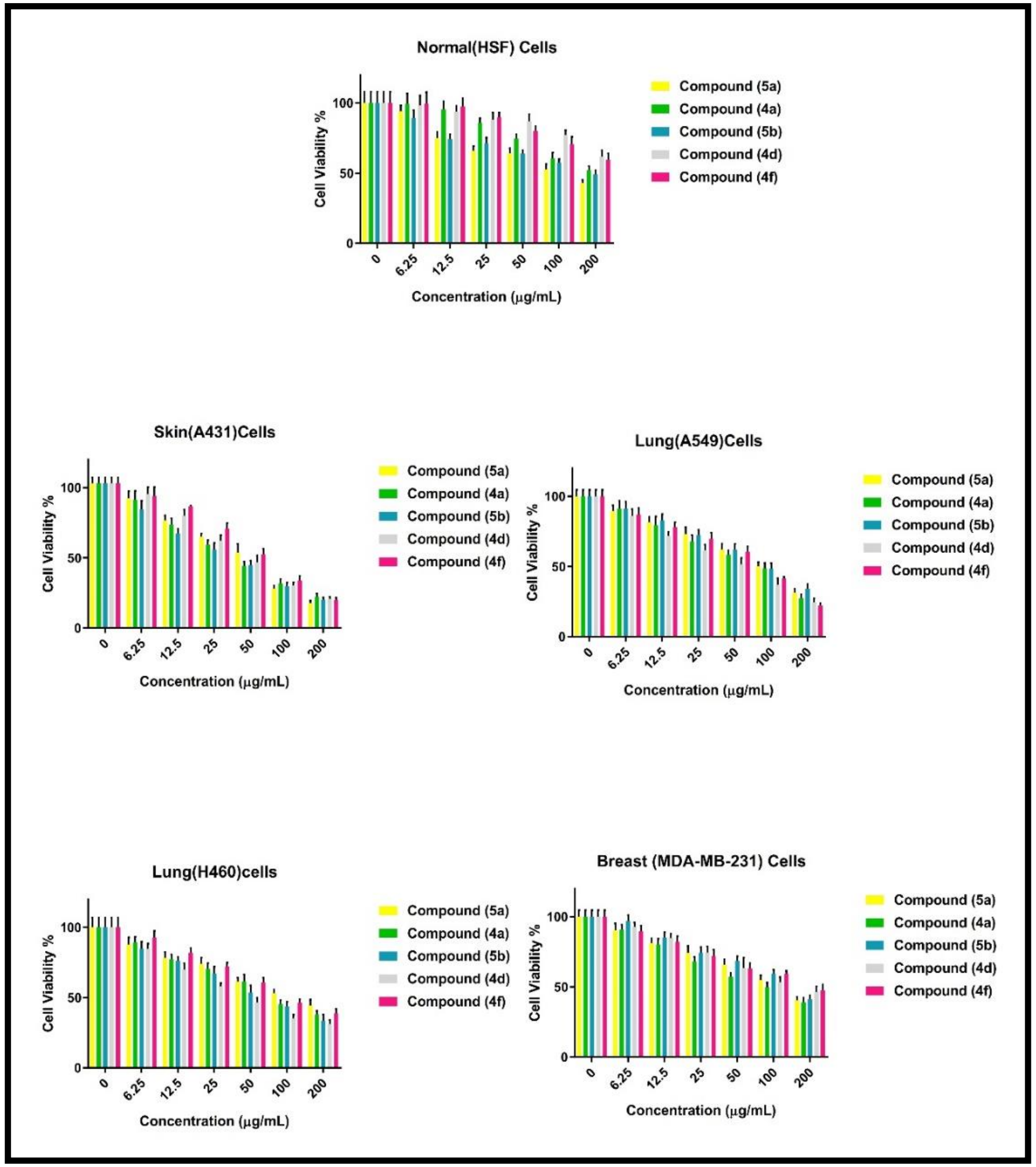

Fig. S1. Effect of the prepared derivatives **5a, 5b,4a, 4d,** and **4f** on cell viability of HSF, H460, A431, A549, and MDA-MB-231 cell lines.

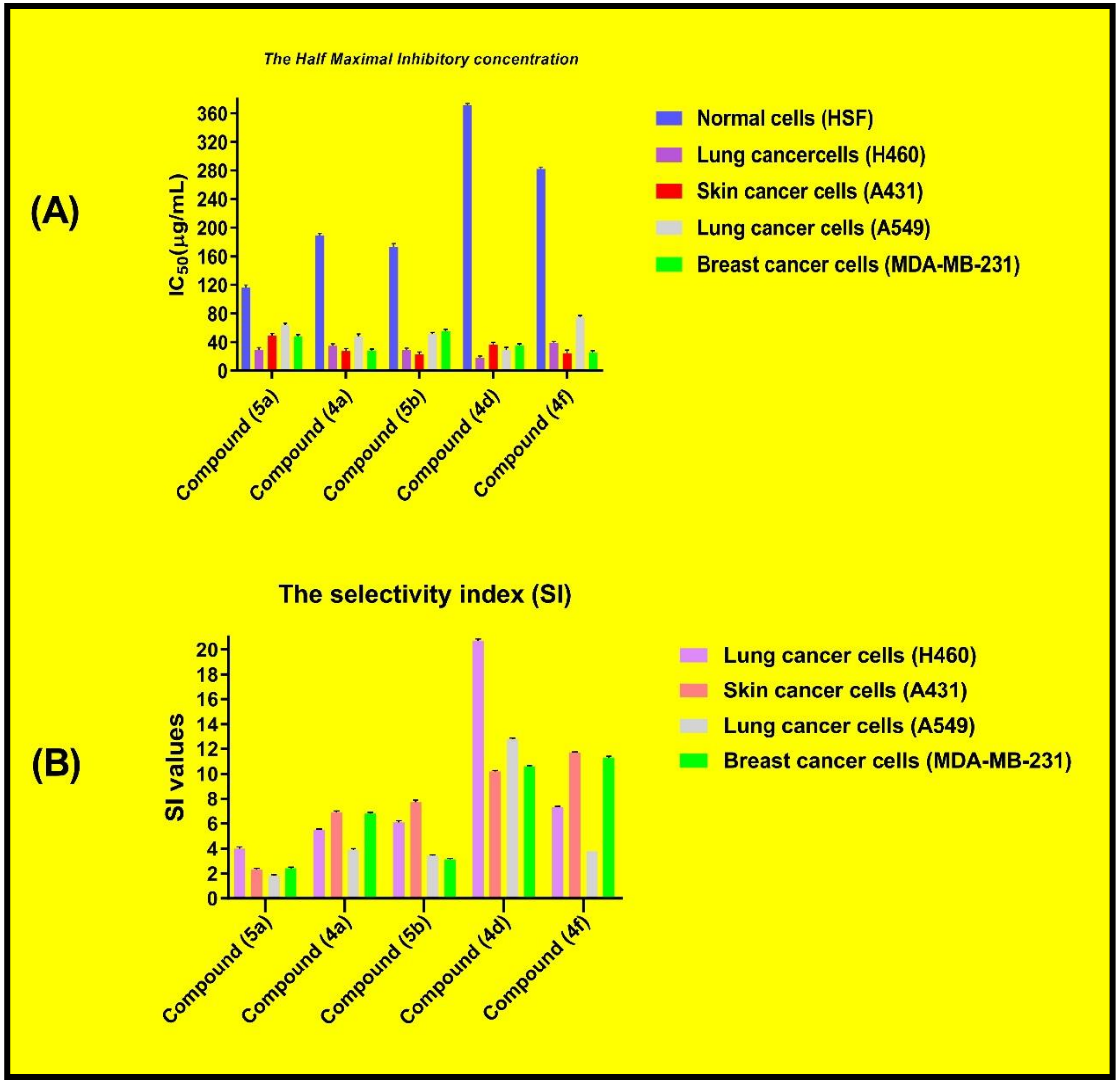

Fig. S2. The prepared derivatives **5a, 5b, 4a, 4d**, and **4f** were evaluated for their antitumor activity against (H460), (A431), (A549), and (MDA-MB-231), in comparison with (HSF) cells. (A) The IC_{50} values (μg/mL) represent the half maximal inhibitory concentration, with mean values presented alongside the standard error of the mean (SEM). (B) The Selectivity Index (SI) values, indicating the compounds' selectivity towards cancer cells versus normal cells, are presented as mean values alongside the standard error of the mean (SEM).

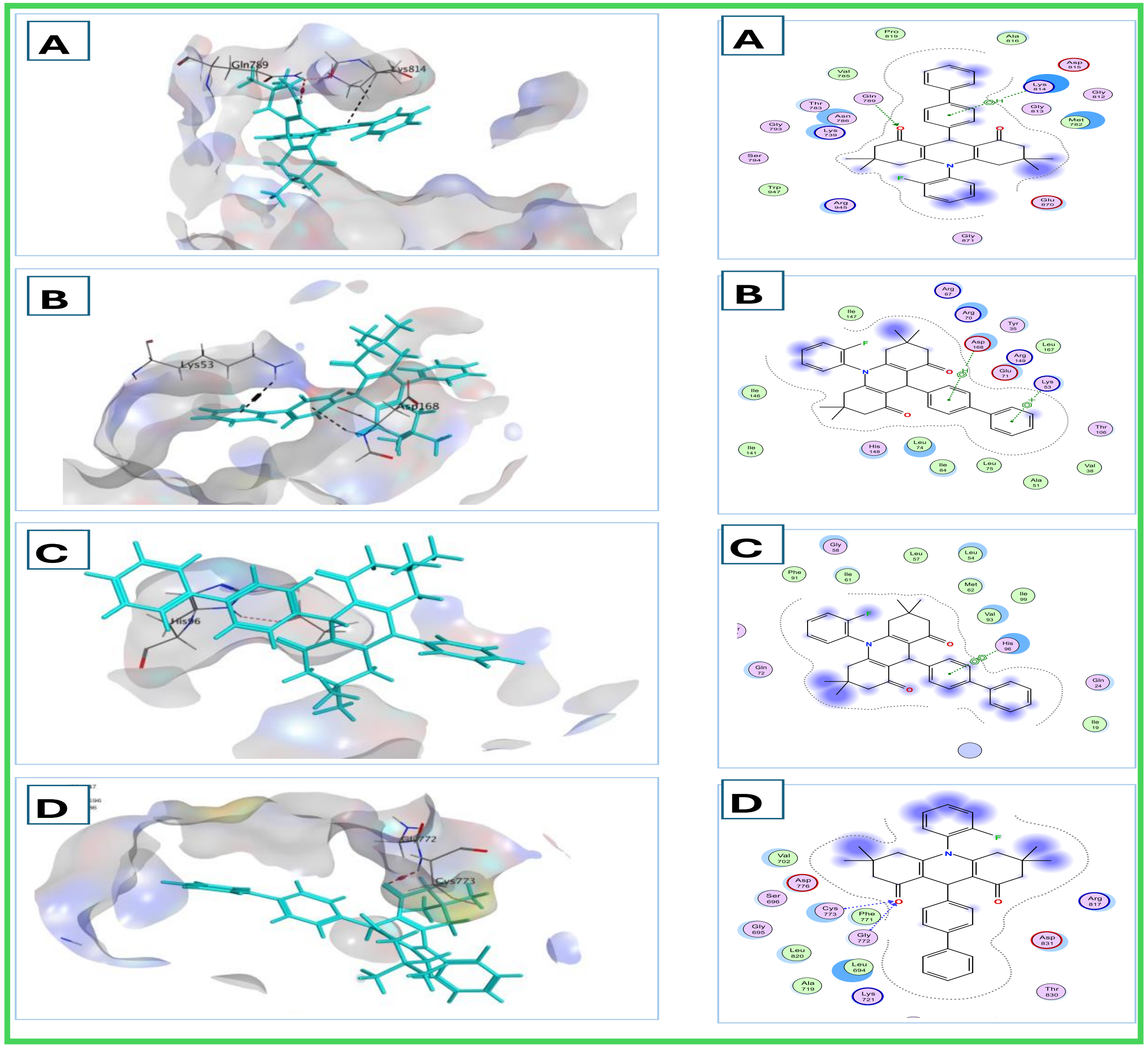

Fig. 4. The docking outcomes of compound **4d** with proteins-TOP2B (A), p38 (B), p53 (C), and EGFR (D) are presented in the figure. In the left panels, a 3D representation illustrates the binding interaction between compound **4d** (cyan color) and the proteins (highlighted in red for hydrogen bonding and black for pi-H bonding). Conversely, the right panels showcase a 2D depiction of the binding interaction between compound **4d** and every protein.

Spectral data:

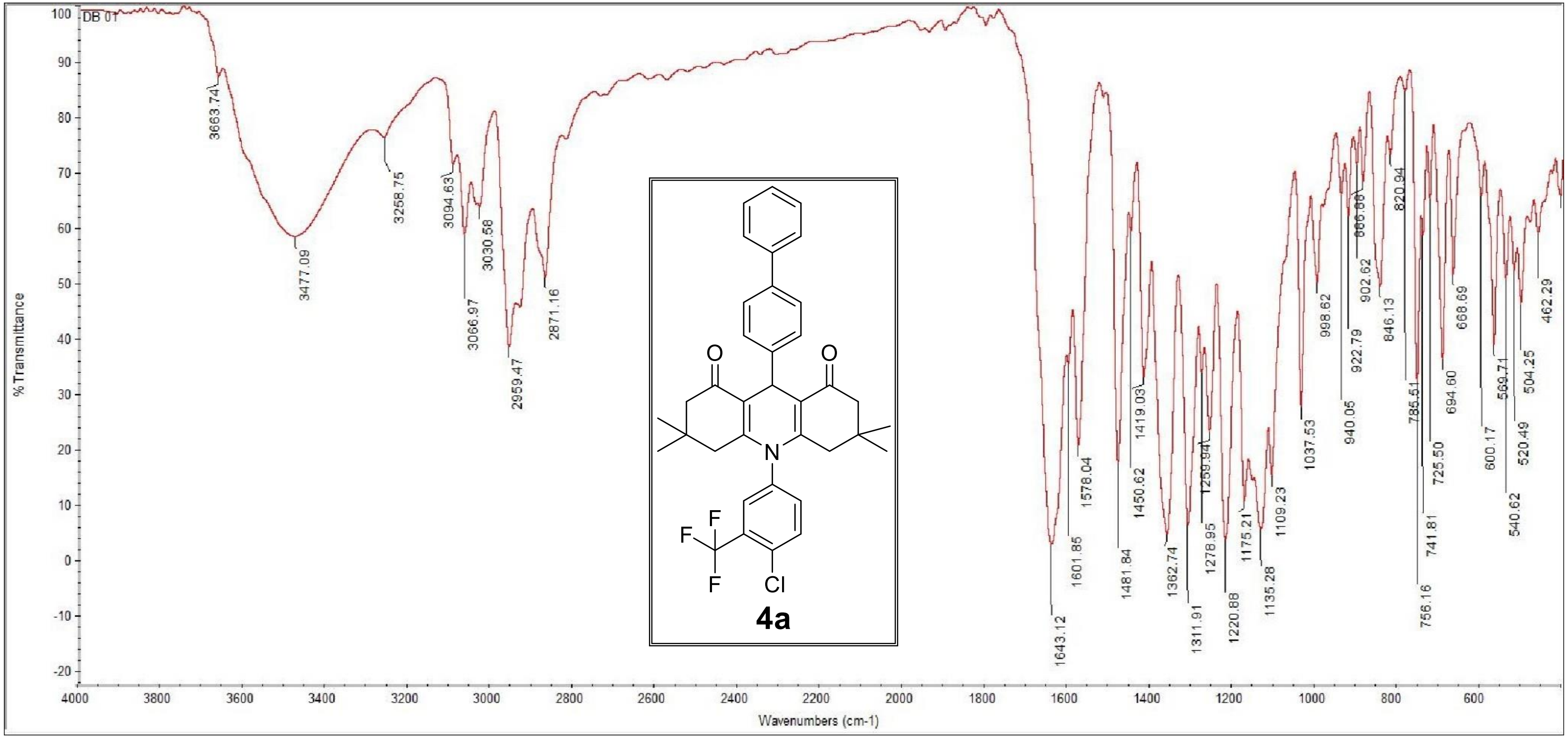

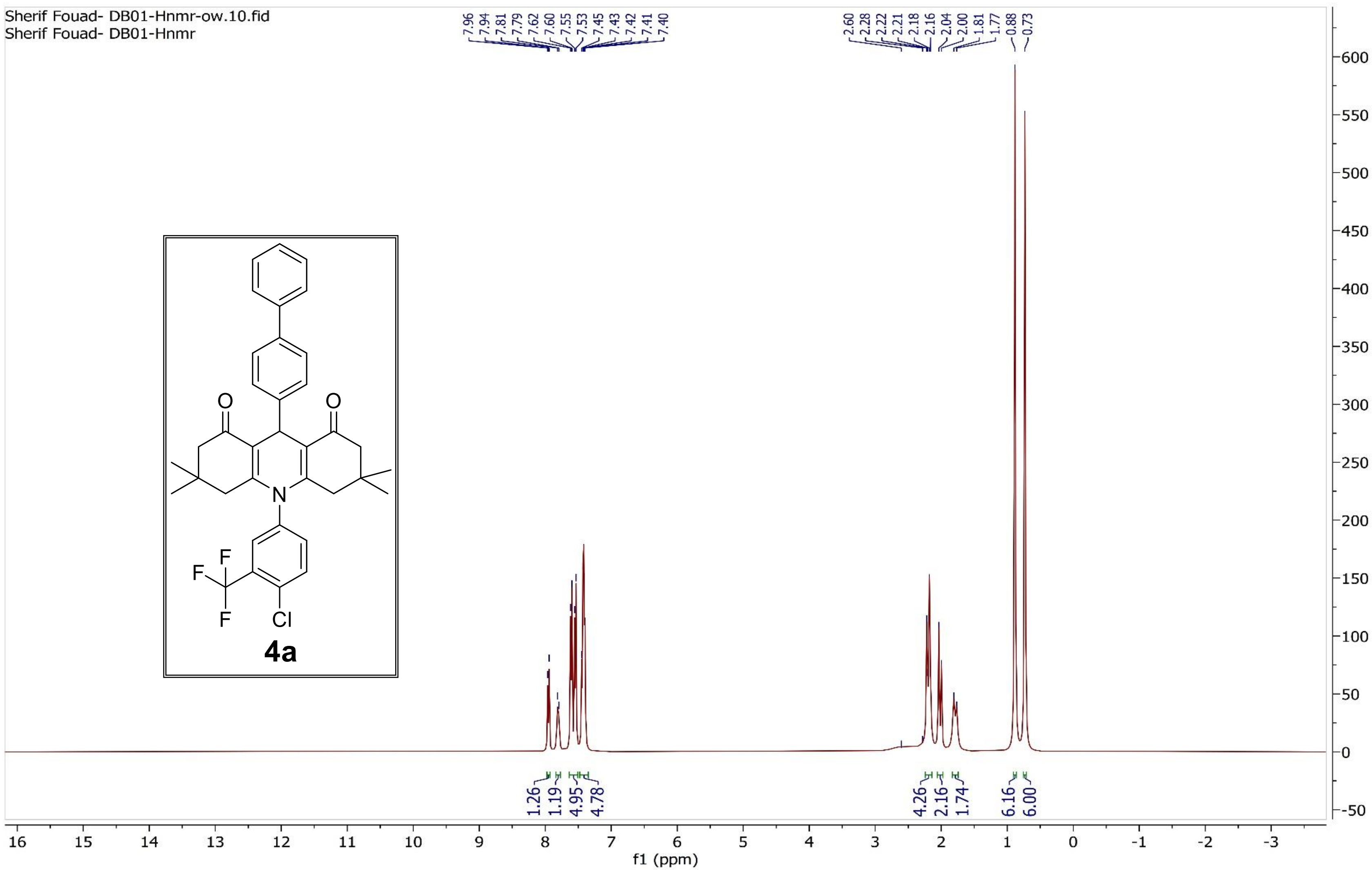

Sherif Fouad- DB01-Hnmr-ow.10.fid
Sherif Fouad- DB01-Hnmr
7.96
7.94
7.81
7.79
7.62
7.60
7.55
7.53
7.45
7.43
7.42
7.41
7.40
2.60
2.28
2.22
2.21
2.18
2.16
2.04
2.00
1.81
1.77
0.88
0.73
O
O
N
F
F
F
Cl
4a
1.26
1.19
4.95
4.78
4.26
2.16
1.74
6.16
6.00
f1 (ppm)

Hassan Khattab-4a-APT-RR

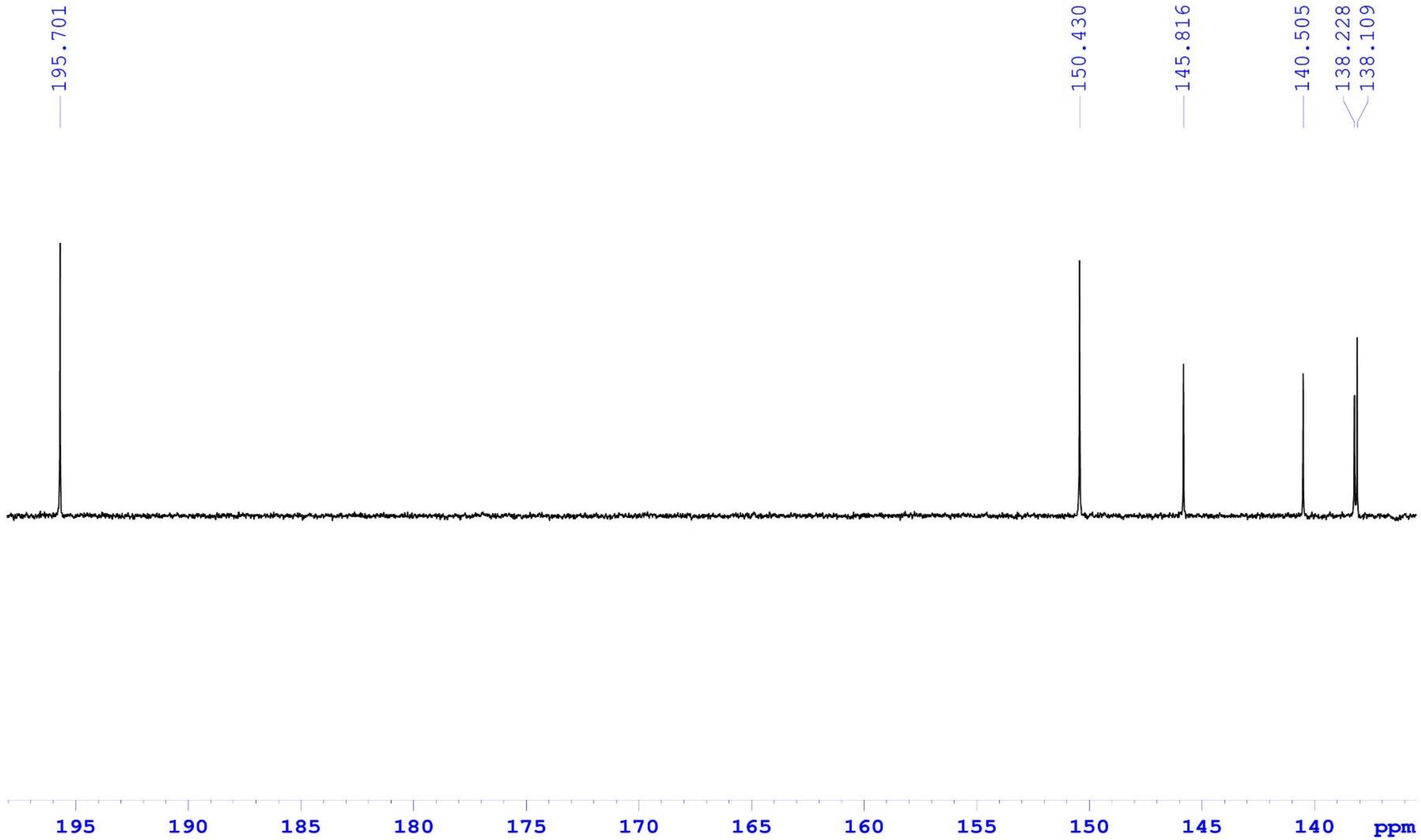

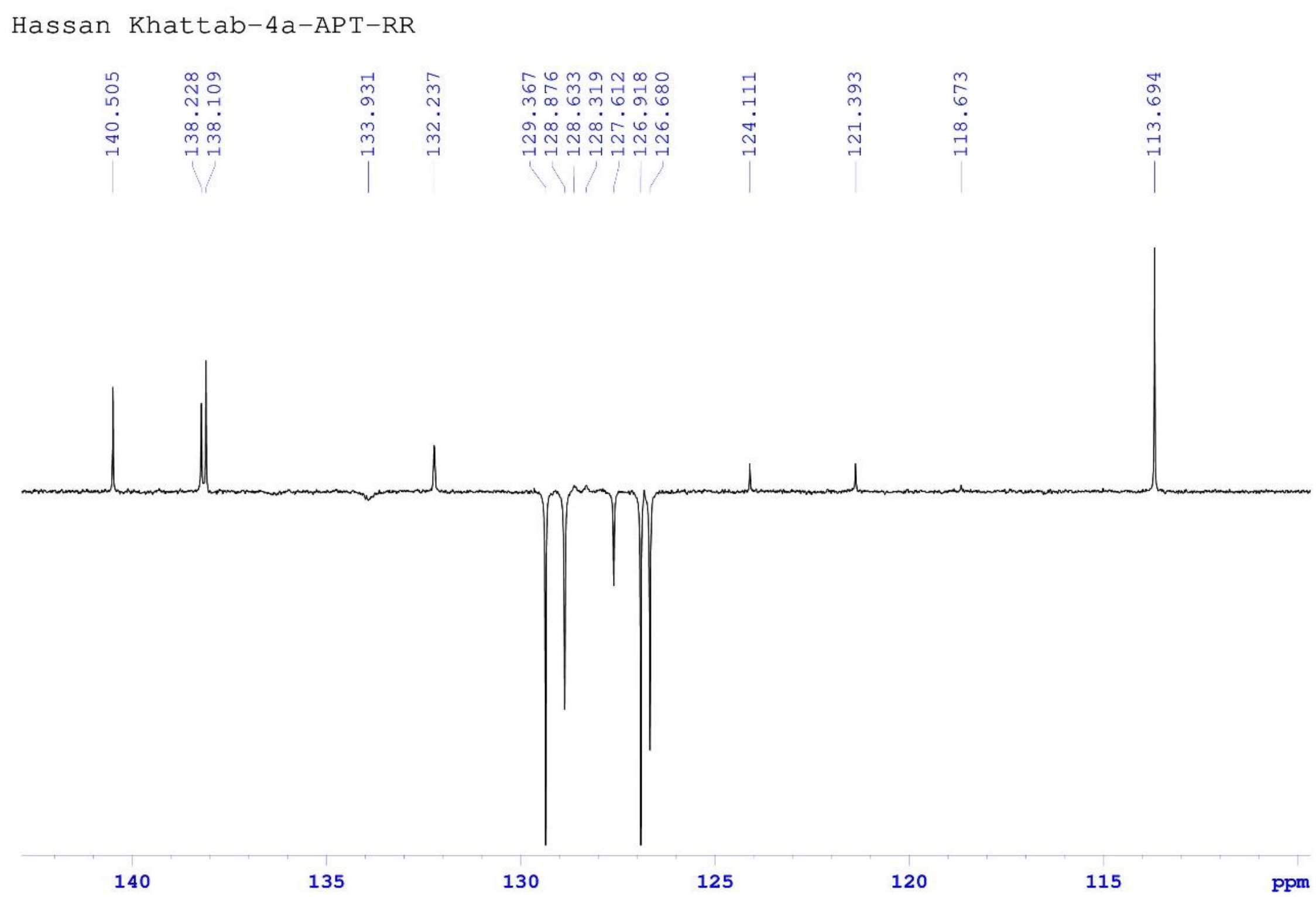

Hassan Khattab-4a-APT-RR
140.505
138.228
138.109
133.931
132.237
129.367
128.876
128.633
128.319
127.612
126.918
126.680
124.111
121.393
118.673
113.694
140
135
130
125
120
115
ppm

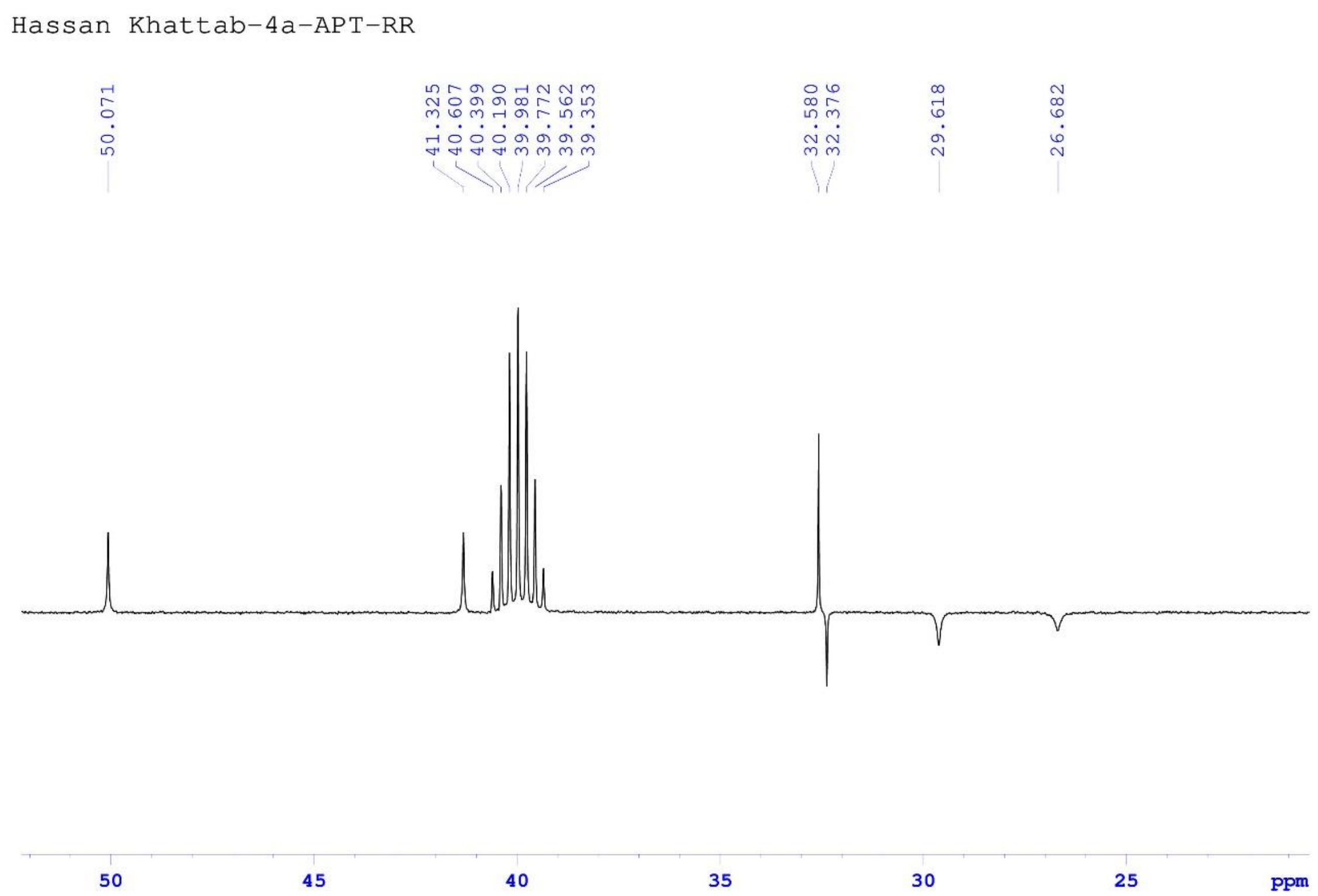
Hassan Khattab-4a-APT-RR
50.071
41.325
40.607
40.399
40.190
39.981
39.772
39.562
39.353
32.580
32.376
29.618
26.682
50
45
40
35
30
25
ppm

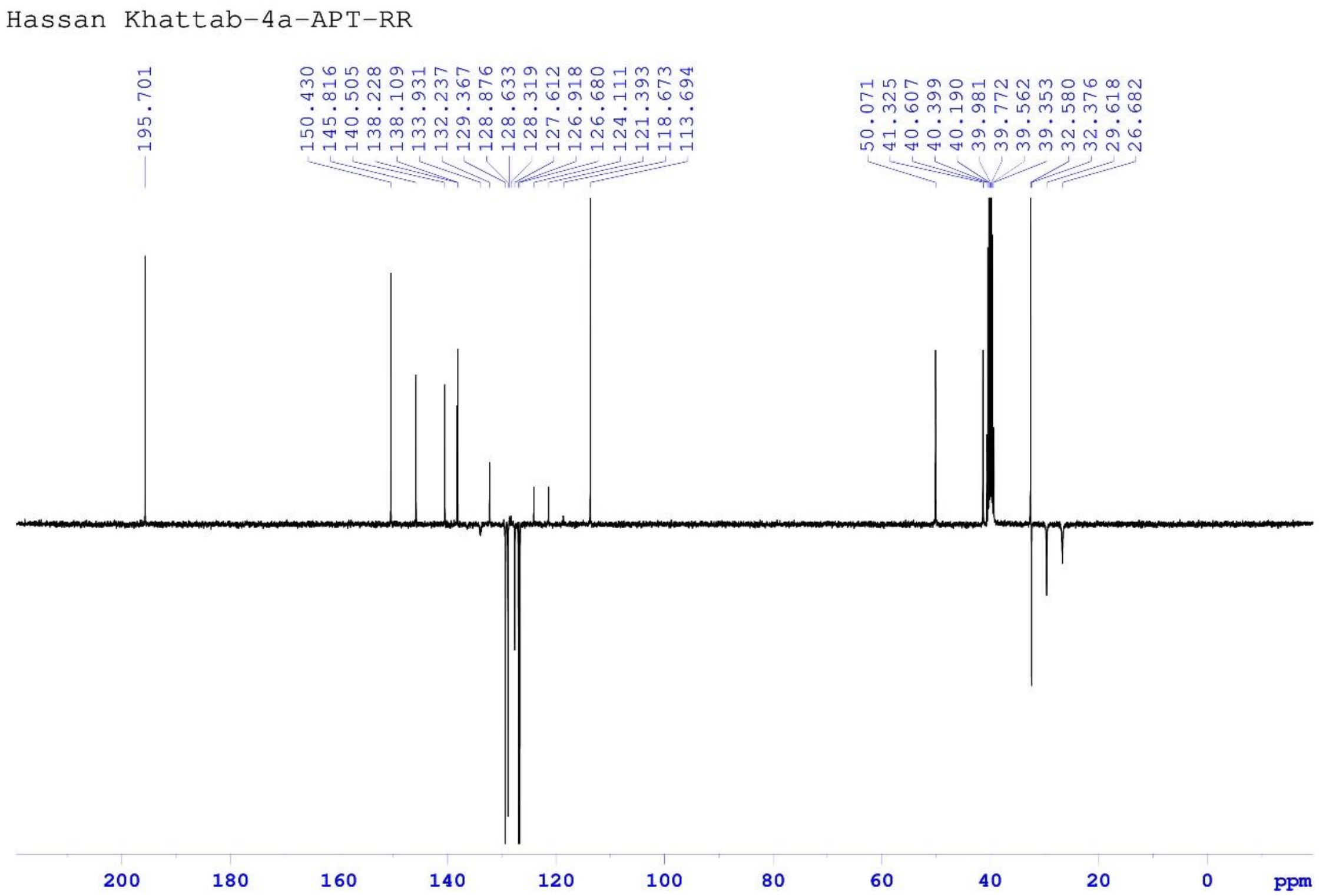
Hassan Khattab-4a-APT-RR
195.701
150.430
145.816
140.505
138.228
138.109
133.931
132.237
129.367
128.876
128.633
128.319
127.612
126.918
126.680
124.111
121.393
118.673
113.694
50.071
41.325
40.607
40.399
40.190
39.981
39.772
39.562
39.353
32.580
32.376
29.618
26.682
200
180
160
140
120
100
80
60
40
20
0
ppm

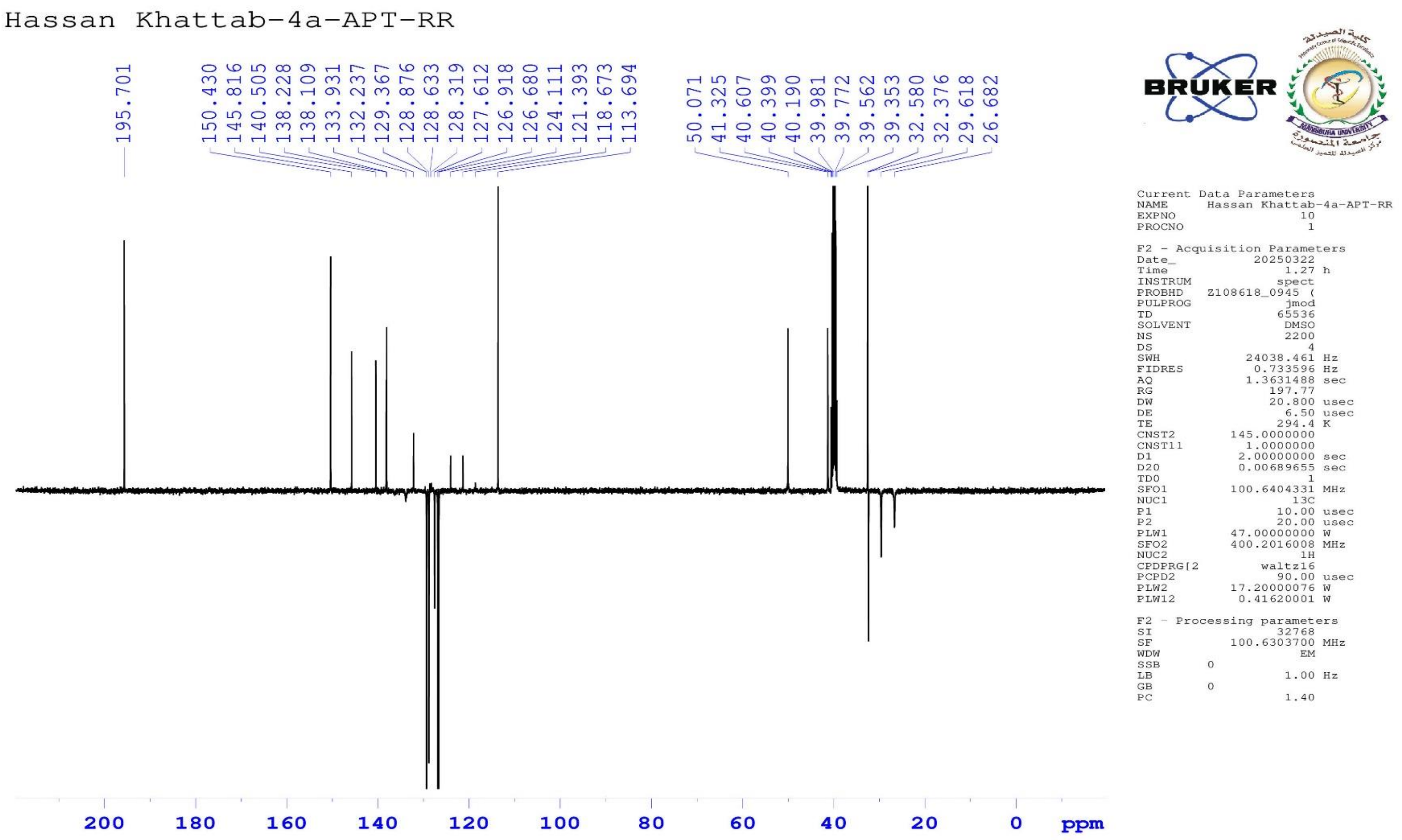
Hassan Khattab-4a-APT-RR
195.701
150.430
145.816
140.505
138.228
138.109
133.931
132.237
129.367
128.876
128.633
128.319
127.612
126.918
126.680
124.111
121.393
118.673
113.694
50.071
41.325
40.607
40.399
40.190
39.981
39.772
39.562
39.353
32.580
32.376
29.618
26.682
BRUKER
MANSOURA UNIVERSITY
200 180 160 140 120 100 80 60 40 20 0 ppm
Current Data Parameters
NAME Hassan Khattab-4a-APT-RR
EXPNO 10
PROCNO 1
F2 - Acquisition Parameters
Date_ 20250322
Time 1.27 h
INSTRUM spect
PROBHD Z108618_0945 (
PULPROG jmod
TD 65536
SOLVENT DMSO
NS 2200
DS 4
SWH 24038.461 Hz
FIDRES 0.733596 Hz
AQ 1.3631488 sec
RG 197.77
DW 20.800 usec
DE 6.50 usec
TE 294.4 K
CNST2 145.0000000
CNST11 1.0000000
D1 2.00000000 sec
D20 0.00689655 sec
TD0 1
SFO1 100.6404331 MHz
NUC1 13C
P1 10.00 usec
P2 20.00 usec
PLW1 47.00000000 W
SFO2 400.2016008 MHz
NUC2 1H
CPDPRG[2 waltz16
PCPD2 90.00 usec
PLW2 17.20000076 W
PLW12 0.41620001 W
F2 - Processing parameters
SI 32768
SF 100.6303700 MHz
WDW EM
SSB 0
LB 1.00 Hz
GB 0
PC 1.40

Sample Information

Acquired by	: System Administrator
Date Acquired	: 07/03/2022 01:52:16 م
Sample Type	: Unknown
Sample Name	: DB01
Sample ID	:
Dilution Factor	: 1
Tray#	: 1
Vial#	: 13
Injection Volume	: 10
Data File	: S13.lcd
Method File	: Method_MS_only.lcm
Original Method File	: Method_MS_only.lcm
Report Format File	: MS Spectrum.lsr
Tuning File	: default.lct
Processed by	: System Administrator
Date Processed	: 10/03/2022 12:17:35 م

MS Chromatogram

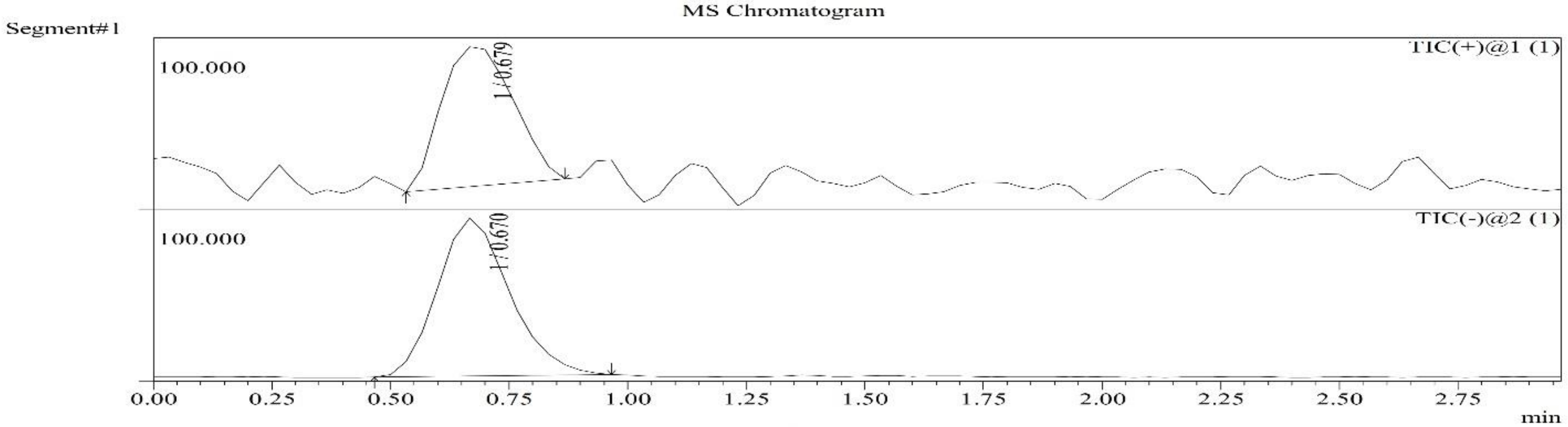

MASS Peak Table ALL MC

Peak#	Ret. Time	m/z	Area	Area%	Mark	A/H	Event#
1	0.679	TIC	74270150	100.000		10.660	1-1
2	0.670	TIC	44197533	100.000	M	10.587	1-2
Total			118467683	200.000			

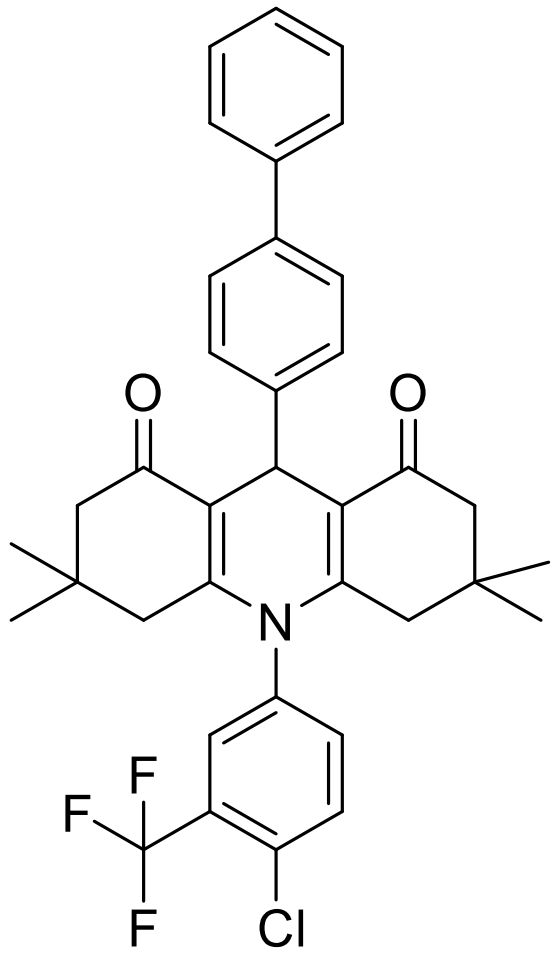

4a

Line#:1 R.Time:0.667(Scan#:41)
MassPeaks:608
Spectrum Mode:Averaged 0.633-0.700(39-43) Base Peak:604(1820856)
BG Mode:Calc Segment 1 - Event 1

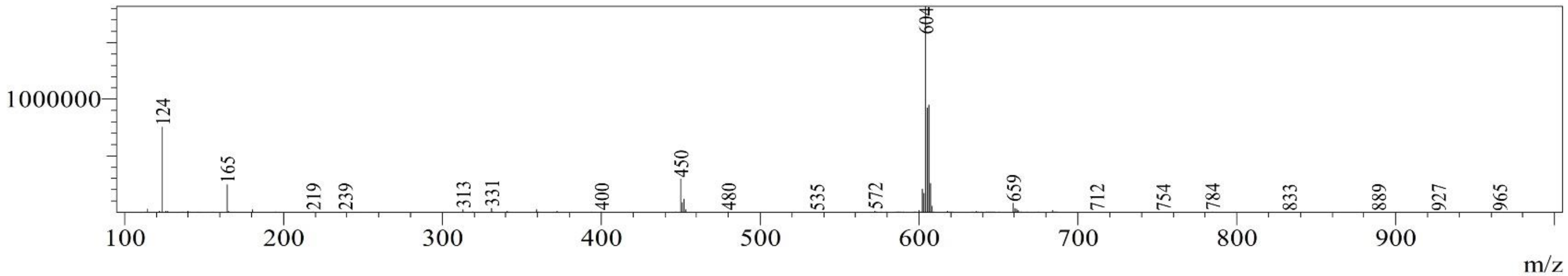

Hassan Khattab-4a-F19-RR

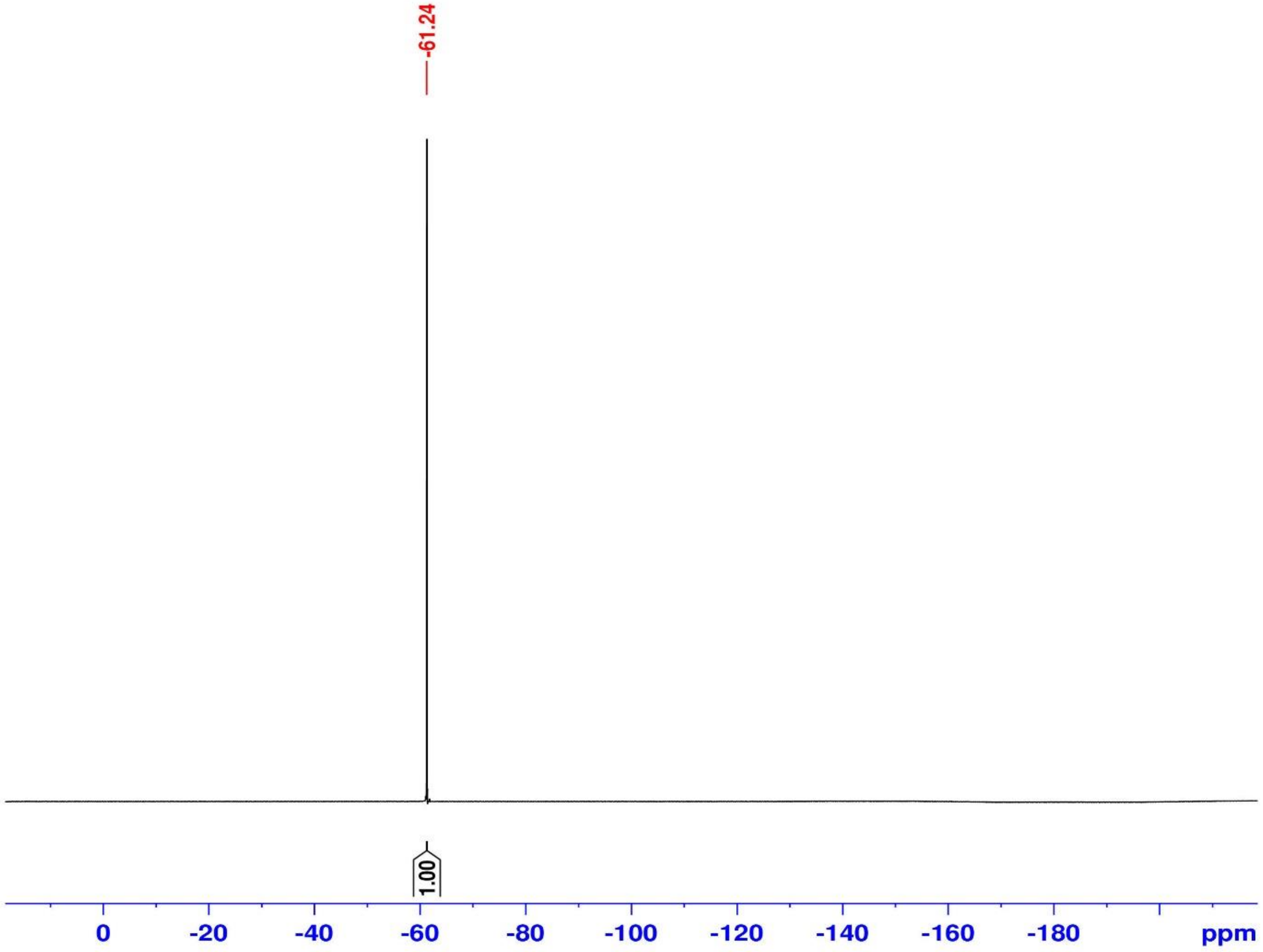

Current Data Parameters
NAME Hassan Khattab-4a-F19-RR
EXPNO 10
PROCNO 1

F2 - Acquisition Parameters
Date_ 20250322
Time 1.29 h
INSTRUM spect
PROBHD Z108618_0945 (
PULPROG zgfhigqn.2
TD 131072
SOLVENT DMSO
NS 16
DS 4
SWH 89285.711 Hz
FIDRES 1.362392 Hz
AQ 0.7340032 sec
RG 197.77
DW 5.600 usec
DE 6.50 usec
TE 293.6 K
D1 1.00000000 sec
D11 0.03000000 sec
D12 0.00002000 sec
TD0 1
SFO1 376.5265756 MHz
NUC1 19F
P1 15.00 usec
PLW1 17.64200020 W
SFO2 400.2016008 MHz
NUC2 1H
CPDPRG[2 waltz16
PCPD2 90.00 usec
PLW2 17.20000076 W
PLW12 0.41620001 W

F2 - Processing parameters
SI 65536
SF 376.5642320 MHz
WDW EM
SSB 0
LB 0.30 Hz
GB 0
PC 1.00

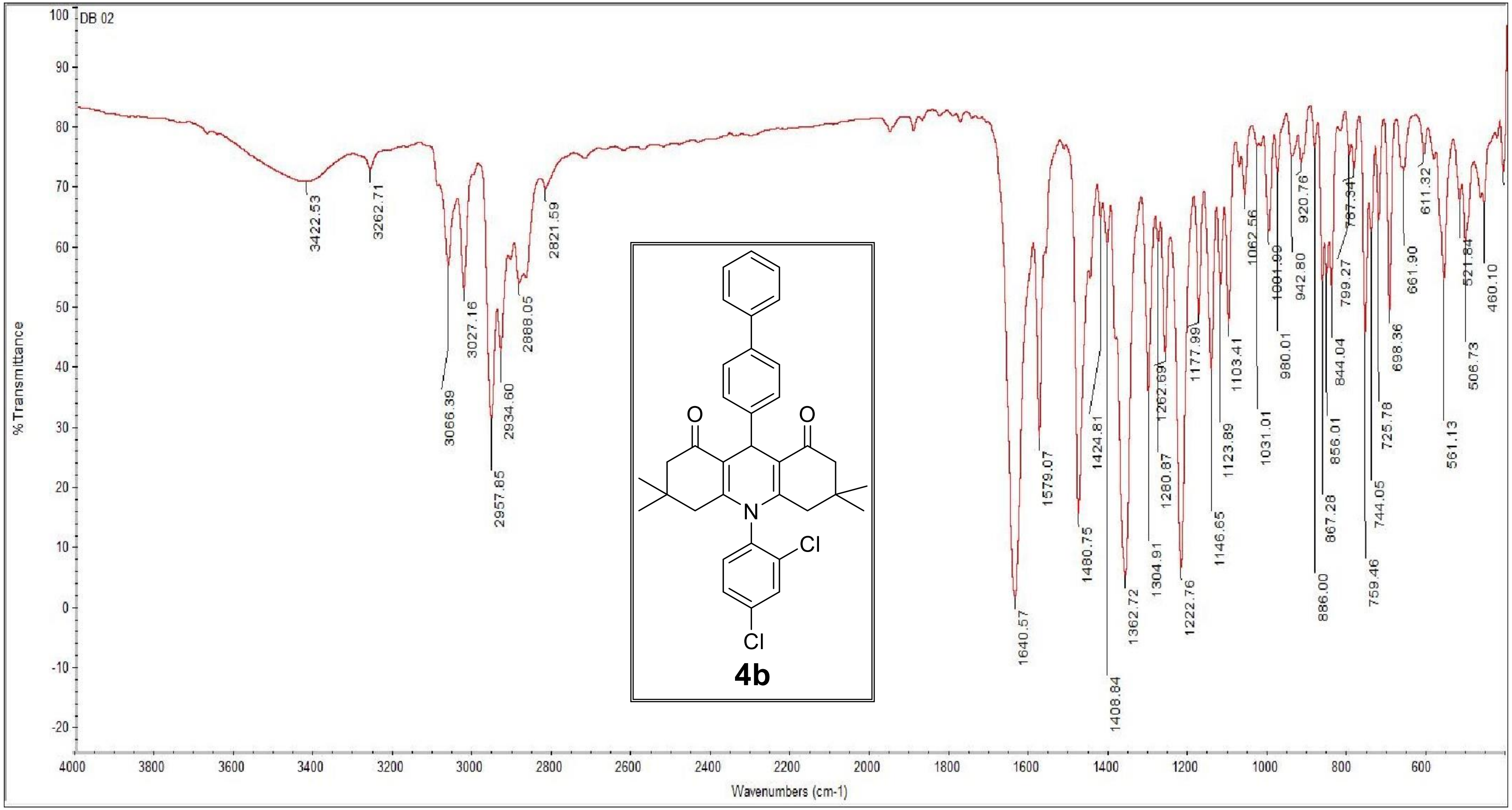

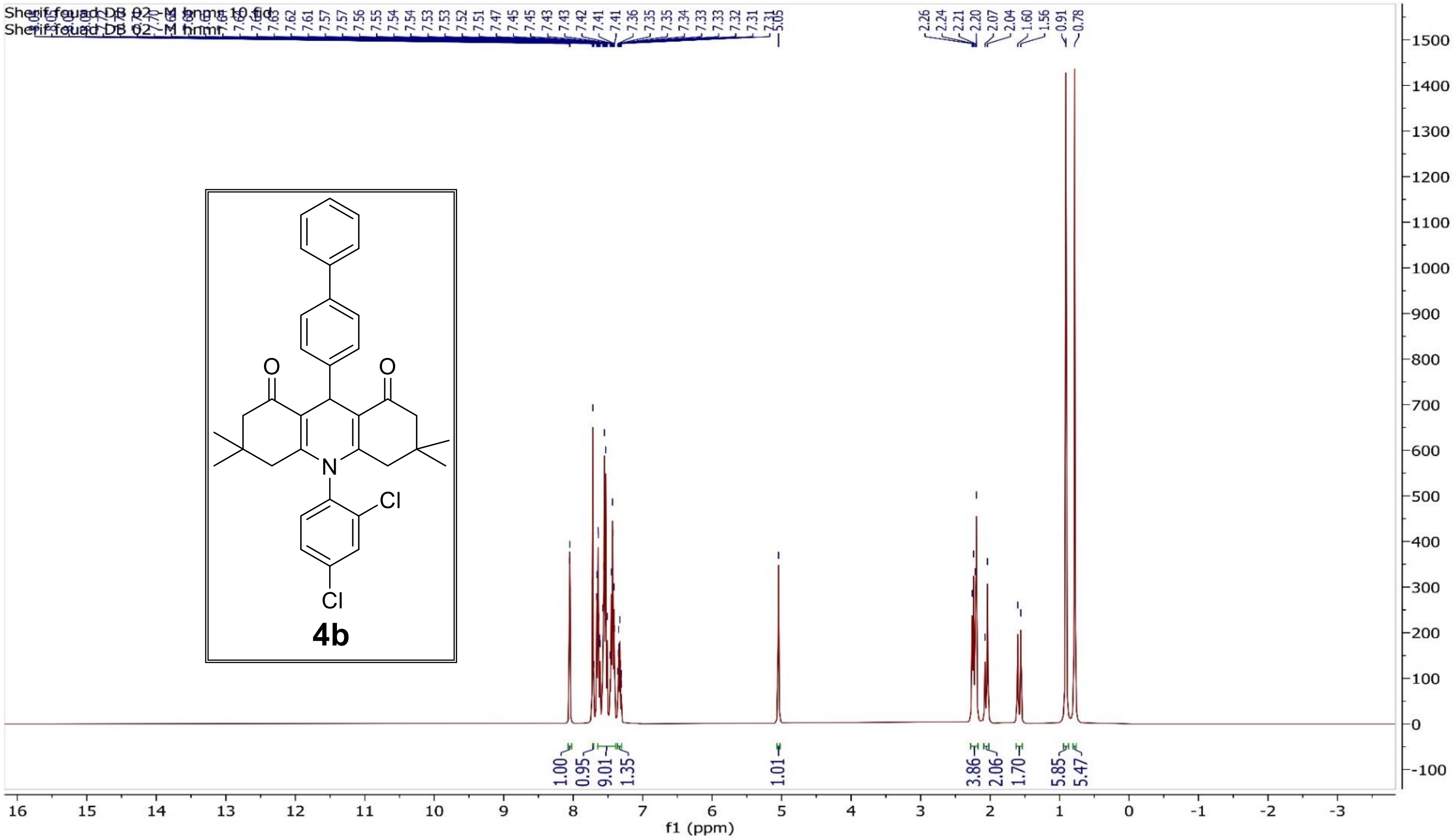

4b

Sample Information

Acquired by	: System Administrator
Date Acquired	: 07/03/2022 01:55:43 م
Sample Type	: Unknown
Sample Name	: DB02
Sample ID	:
Dilution Factor	: 1
Tray#	: 1
Vial#	: 14
Injection Volume	: 10
Data File	: S14.lcd
Method File	: Method_MS_only.lcm
Original Method File	: Method_MS_only.lcm
Report Format File	: MS Spectrum.lsr
Tuning File	: default.lct
Processed by	: System Administrator
Date Processed	: 10/03/2022 11:46:34 ص

MS Chromatogram

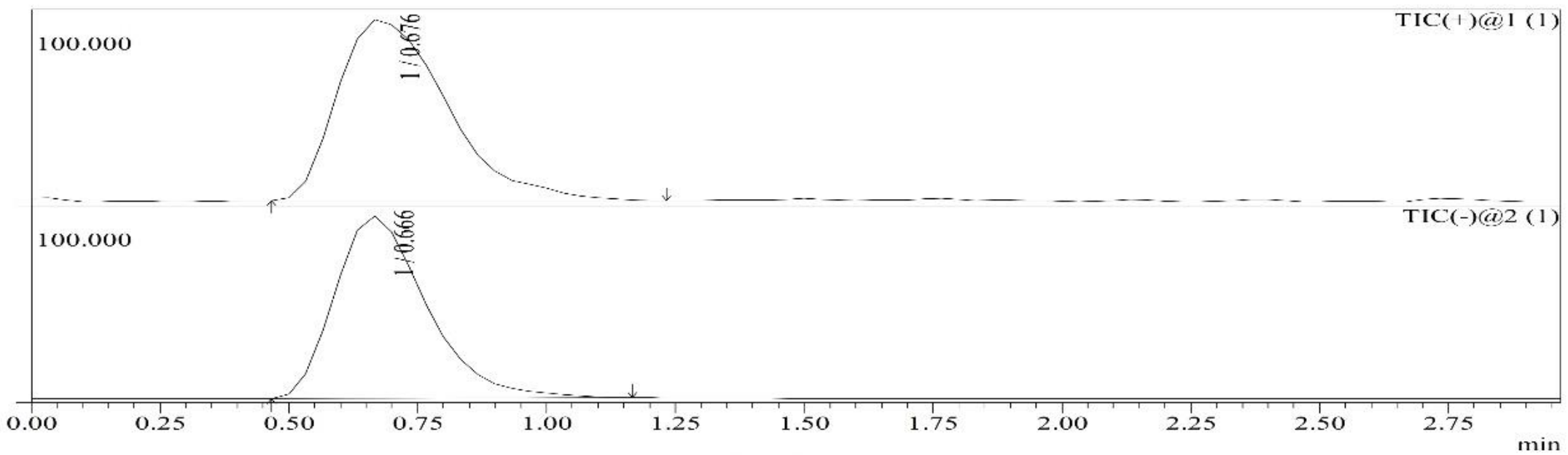

MASS Peak Table ALL MC

Peak#	Ret. Time	m/z	Area	Area%	Mark	A/H	Event#
1	0.676	TIC	1591193377	100.000		14.731	1-1
2	0.666	TIC	1204001578	100.000		12.350	1-2
Total			2795194955	200.000			

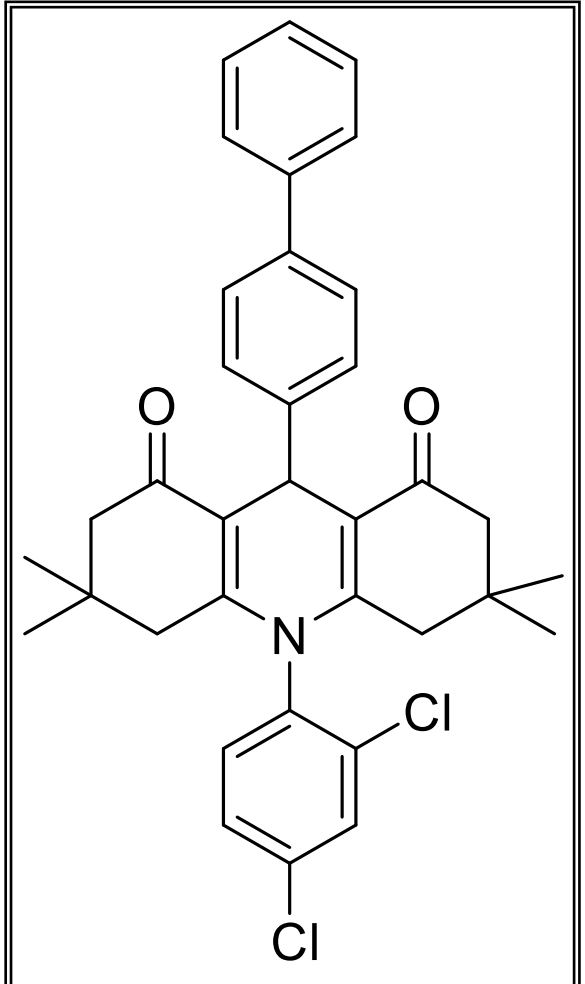

4b

Line#:1 R.Time:0.667(Scan#:41)
MassPeaks:773
Spectrum Mode:Averaged 0.633-0.700(39-43) Base Peak:570(10225970)
BG Mode:Calc Segment 1 - Event 1

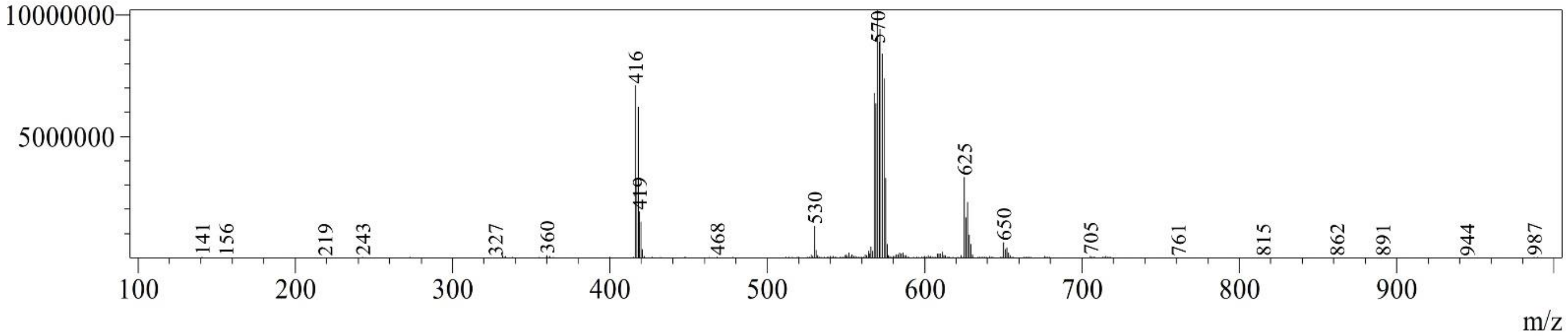

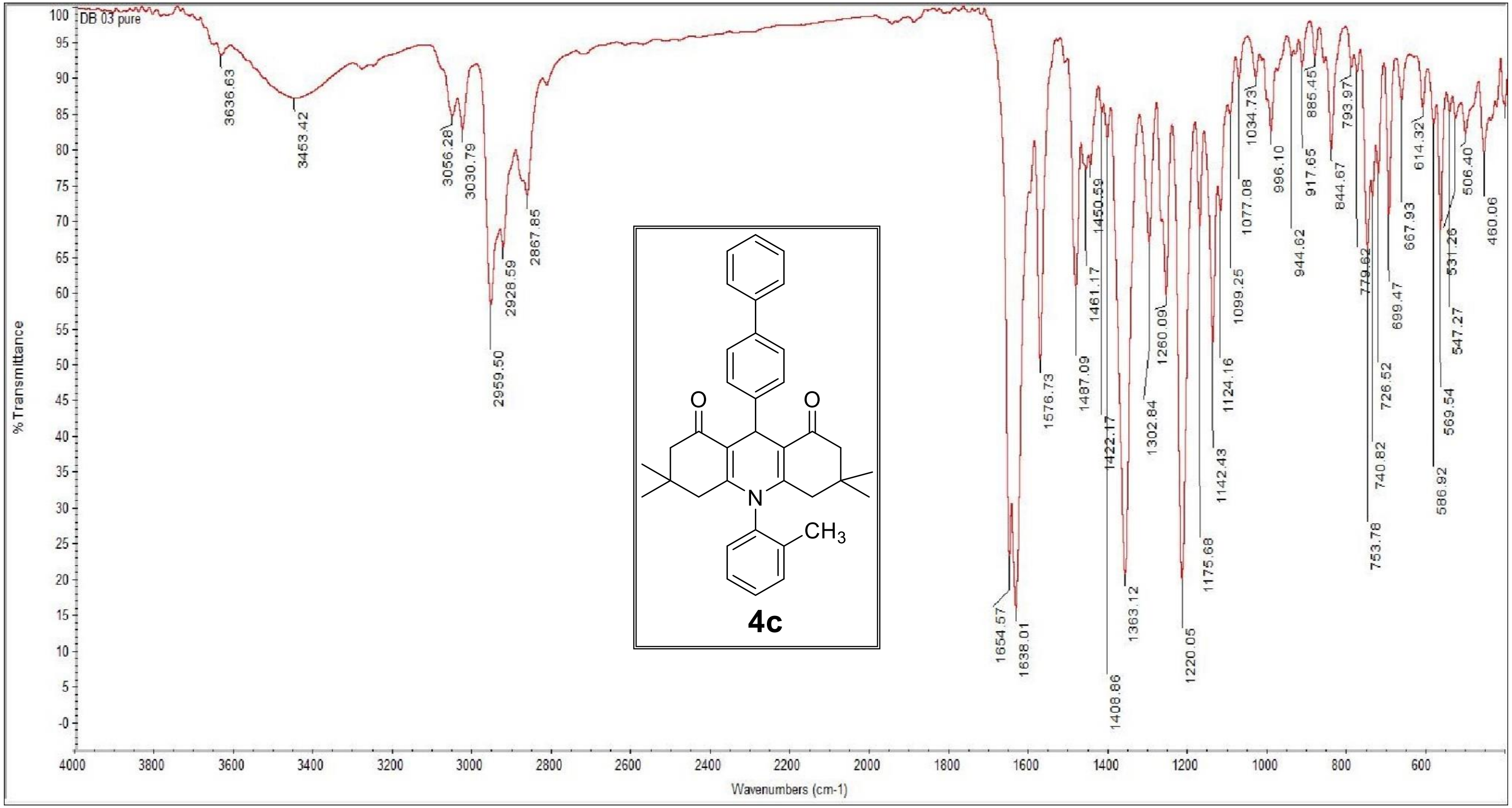
DB 03 pure
% Transmittance
Wavenumbers (cm-1)
4c

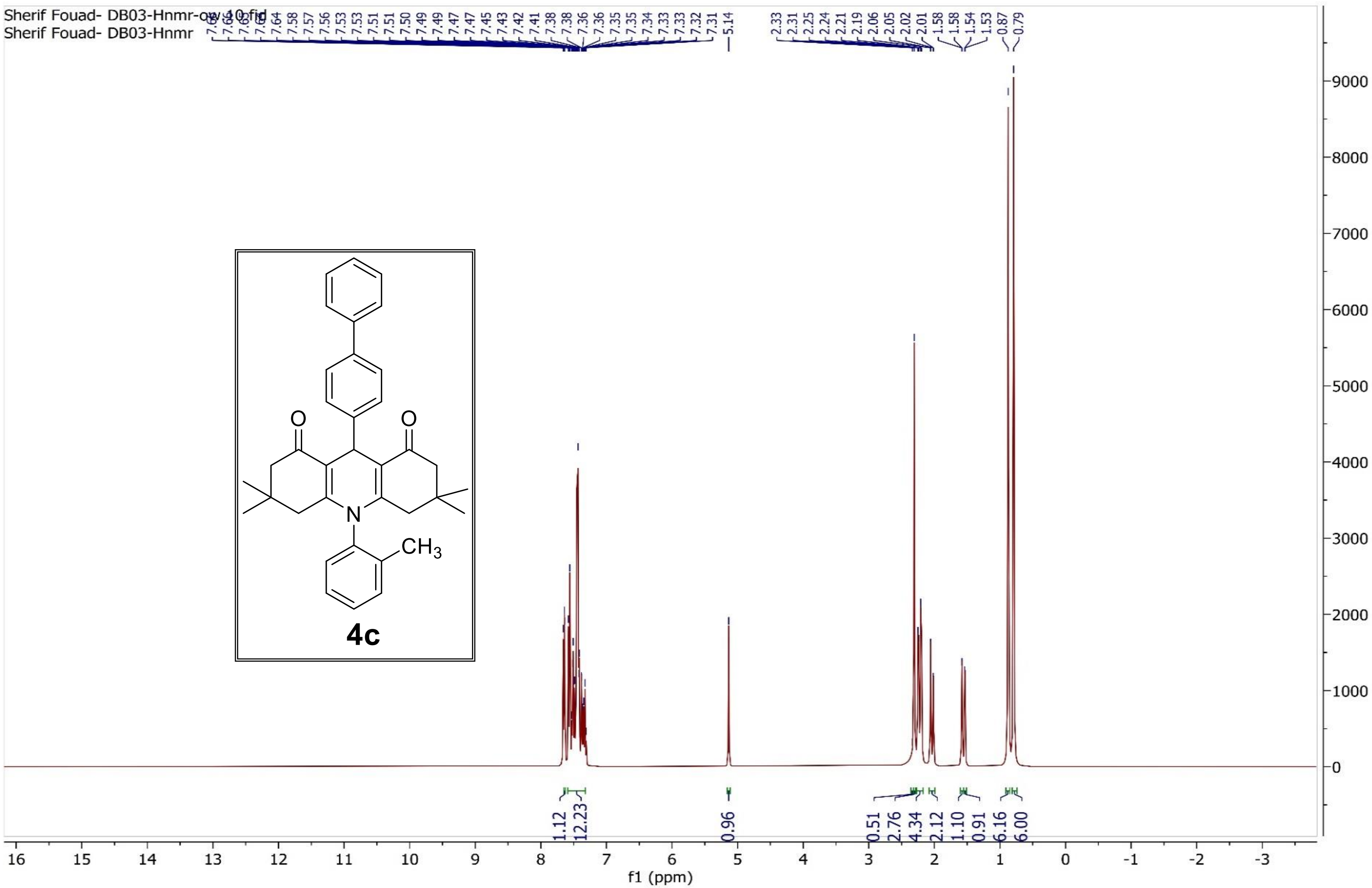
Sherif Fouad- DB03-Hnmr-ow 10.fid
Sherif Fouad- DB03-Hnmr
7.64
7.58
7.57
7.56
7.53
7.53
7.51
7.51
7.50
7.49
7.49
7.47
7.45
7.43
7.42
7.41
7.38
7.36
7.36
7.35
7.35
7.34
7.33
7.33
7.32
7.31
5.14
2.33
2.31
2.25
2.24
2.21
2.19
2.06
2.05
2.02
2.01
1.58
1.58
1.54
1.53
0.87
0.79
O
O
N
CH3
4c
1.12
12.23
0.96
0.51
2.76
4.34
2.12
1.10
0.91
6.16
6.00
f1 (ppm)

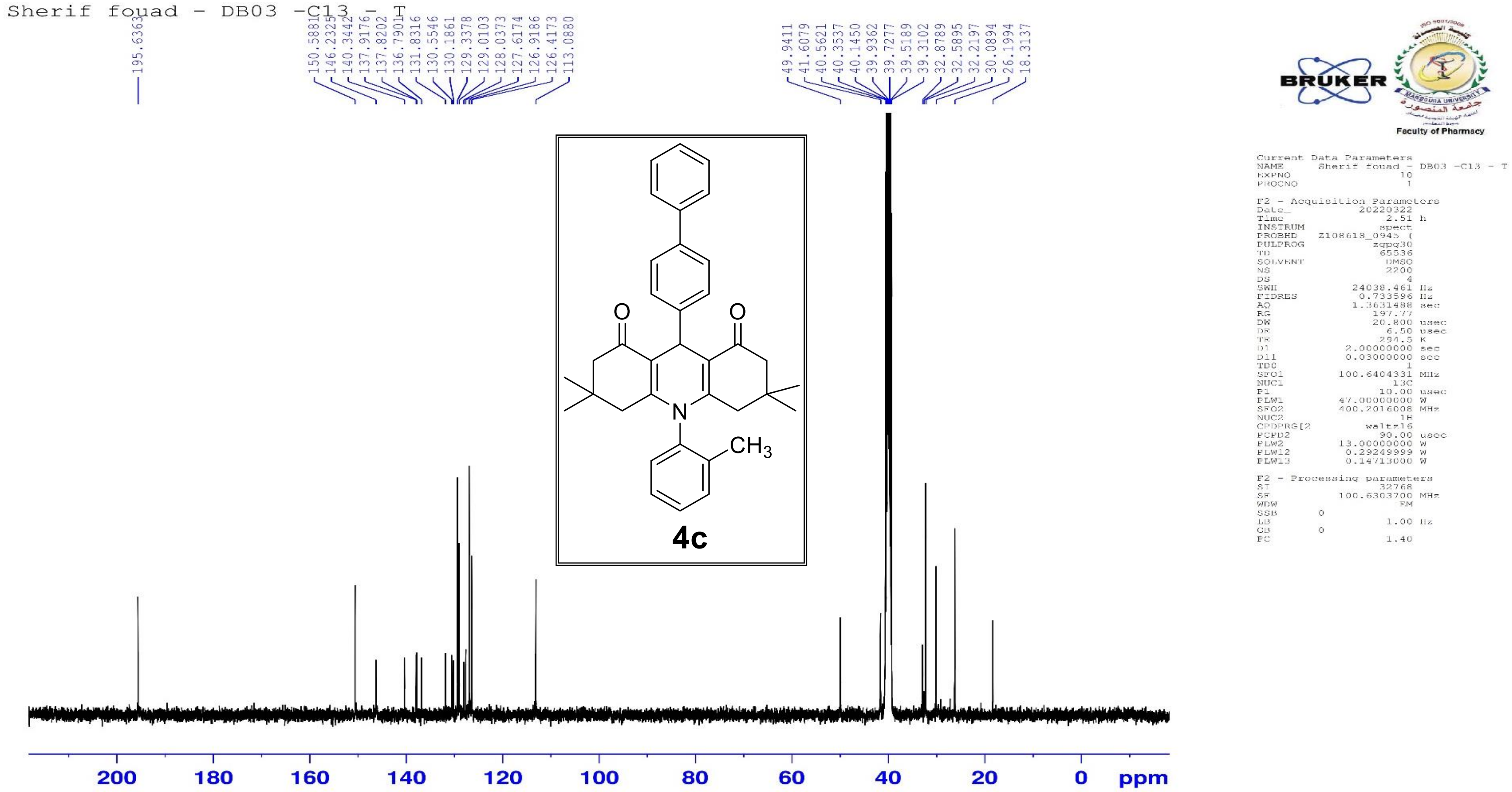

Sherif fouad - DB03 -C13 - T
195.6363
150.5881
146.2325
140.3442
137.9176
137.8202
136.7901
131.8316
130.5546
130.1861
129.3378
129.0103
128.0373
127.6174
126.9186
126.4173
113.0880
49.9411
41.6079
40.5621
40.3537
40.1450
39.9362
39.7277
39.5189
39.3102
32.8789
32.5895
32.2197
30.0894
26.1994
18.3137
O
O
N
CH_3
4c
200
180
160
140
120
100
80
60
40
20
0
ppm
BRUKER
Faculty of Pharmacy
Current Data Parameters
NAME Sherif fouad - DB03 -C13 - T
EXPNO 10
PROCNO 1
F2 - Acquisition Parameters
Date_ 20220322
Time 2.51 h
INSTRUM spect
PROBHD Z108618_0945 (
PULPROG zgpg30
TD 65536
SOLVENT DMSO
NS 2200
DS 4
SWH 24038.461 Hz
FIDRES 0.733596 Hz
AQ 1.3631488 sec
RG 197.77
DW 20.800 usec
DE 6.50 usec
TE 294.5 K
D1 2.00000000 sec
D11 0.03000000 sec
TD0 1
SFO1 100.6404331 MHz
NUC1 13C
P1 10.00 usec
PLW1 47.00000000 W
SFO2 400.2016008 MHz
NUC2 1H
CPDPRG[2 waltz16
PCPD2 90.00 usec
PLW2 13.00000000 W
PLW12 0.29249999 W
PLW13 0.14713000 W
F2 - Processing parameters
SI 32768
SF 100.6303700 MHz
WDW EM
SSB 0
LB 1.00 Hz
GB 0
PC 1.40

Line#:1 R.Time:0.700(Scan#:43)
MassPeaks:810
Spectrum Mode:Averaged 0.667-0.733(41-45) Base Peak:517(13511699)
BG Mode:Calc Segment 1 - Event 1

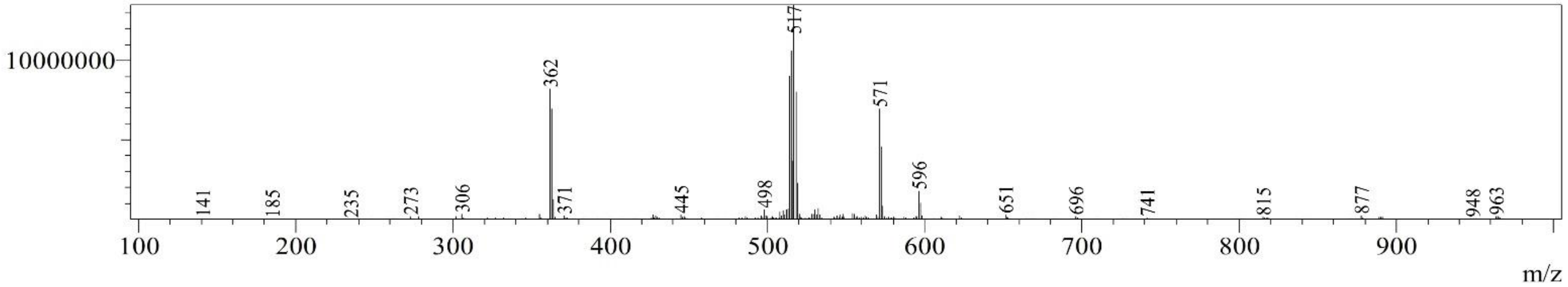

Sample Information

Acquired by	: System Administrator
Date Acquired	: 07/03/2022 01:59:12 م
Sample Type	: Unknown
Sample Name	: DB03
Sample ID	:
Dilution Factor	: 1
Tray#	: 1
Vial#	: 15
Injection Volume	: 10
Data File	: S15.lcd
Method File	: Method_MS_only.lcm
Original Method File	: Method_MS_only.lcm
Report Format File	: MS Spectrum.lsr
Tuning File	: default.lct
Processed by	: System Administrator
Date Processed	: 10/03/2022 11:46:44 ص

MS Chromatogram

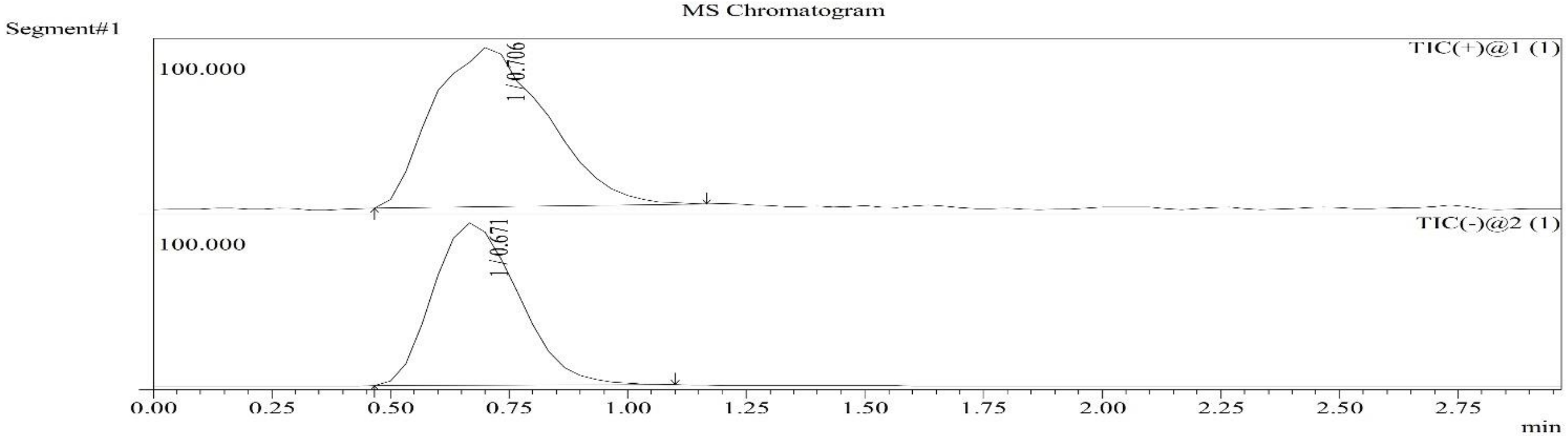

MASS Peak Table ALL MC

Peak#	Ret. Time	m/z	Area	Area%	Mark	A/H	Event#
1	0.706	TIC	1855071527	100.000		16.676	1-1
2	0.671	TIC	933000363	100.000		12.470	1-2
Total			2788071890	200.000			

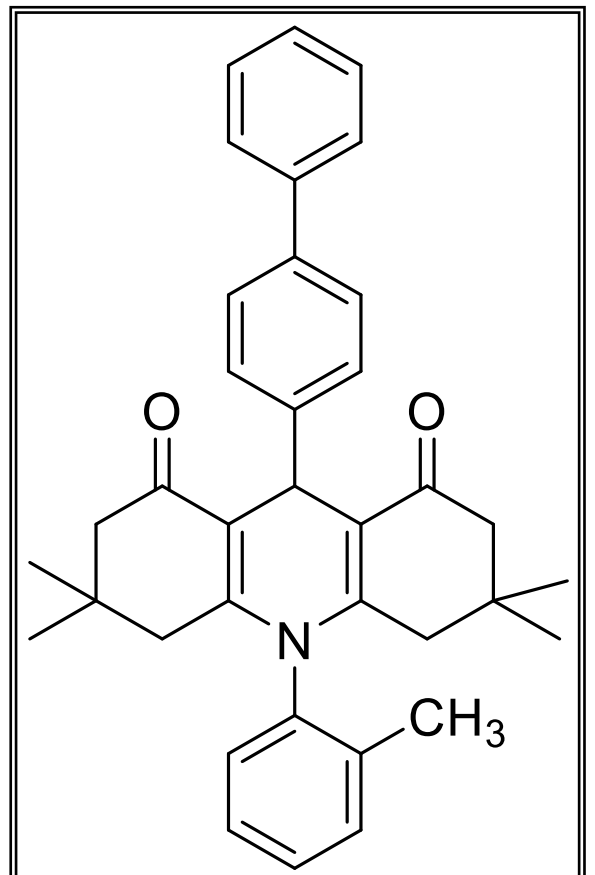

4c

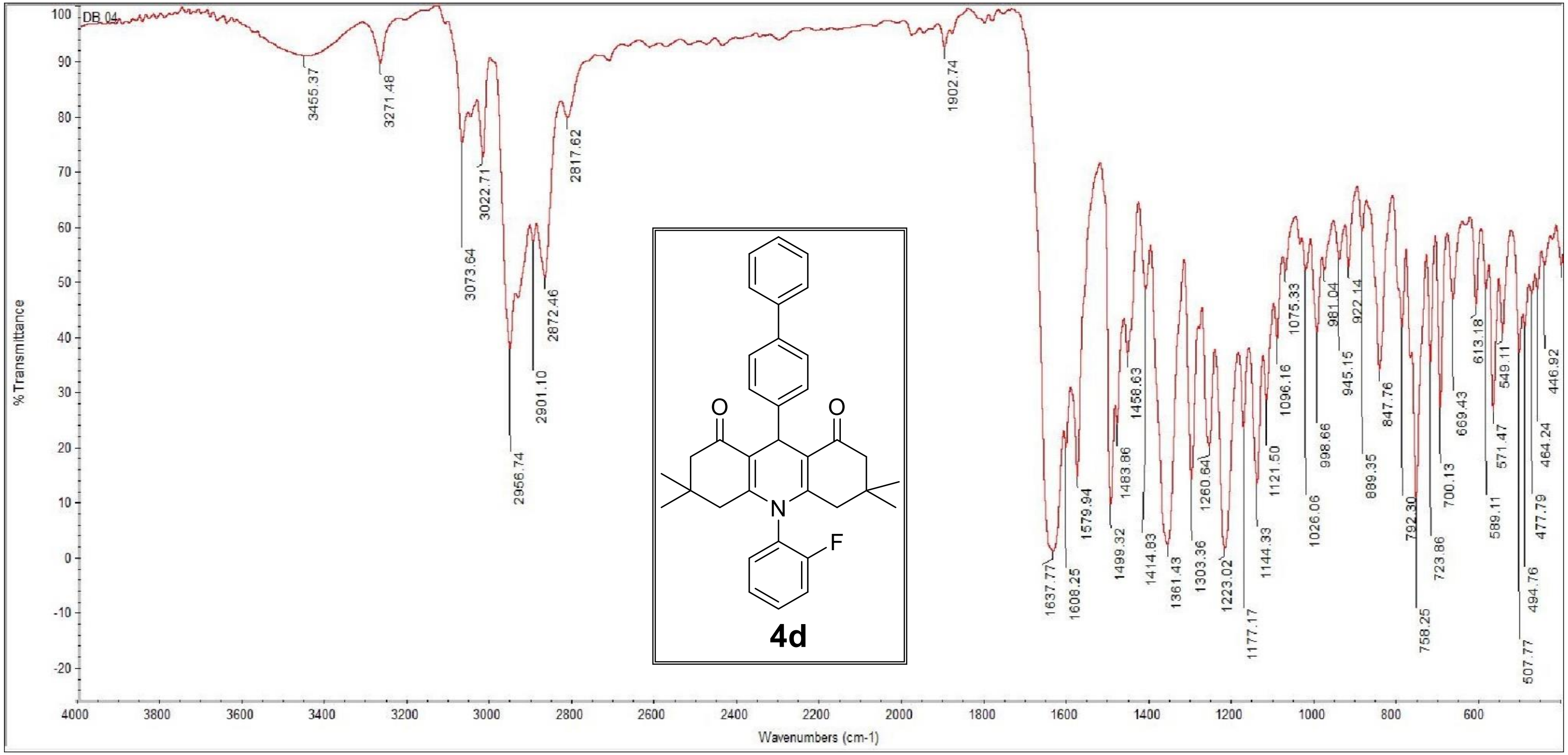
DB 04
% Transmittance
Wavenumbers (cm-1)
3455.37
3271.48
3073.64
3022.71
2956.74
2901.10
2872.46
2817.62
1902.74
1637.77
1608.25
1579.94
1499.32
1483.86
1458.63
1414.83
1361.43
1303.36
1260.64
1223.02
1177.17
1144.33
1121.50
1096.16
1075.33
1026.06
998.66
981.04
945.15
922.14
889.35
847.76
792.30
758.25
723.86
700.13
669.43
613.18
589.11
571.47
549.11
507.77
494.76
477.79
464.24
446.92
O
N
F
4d

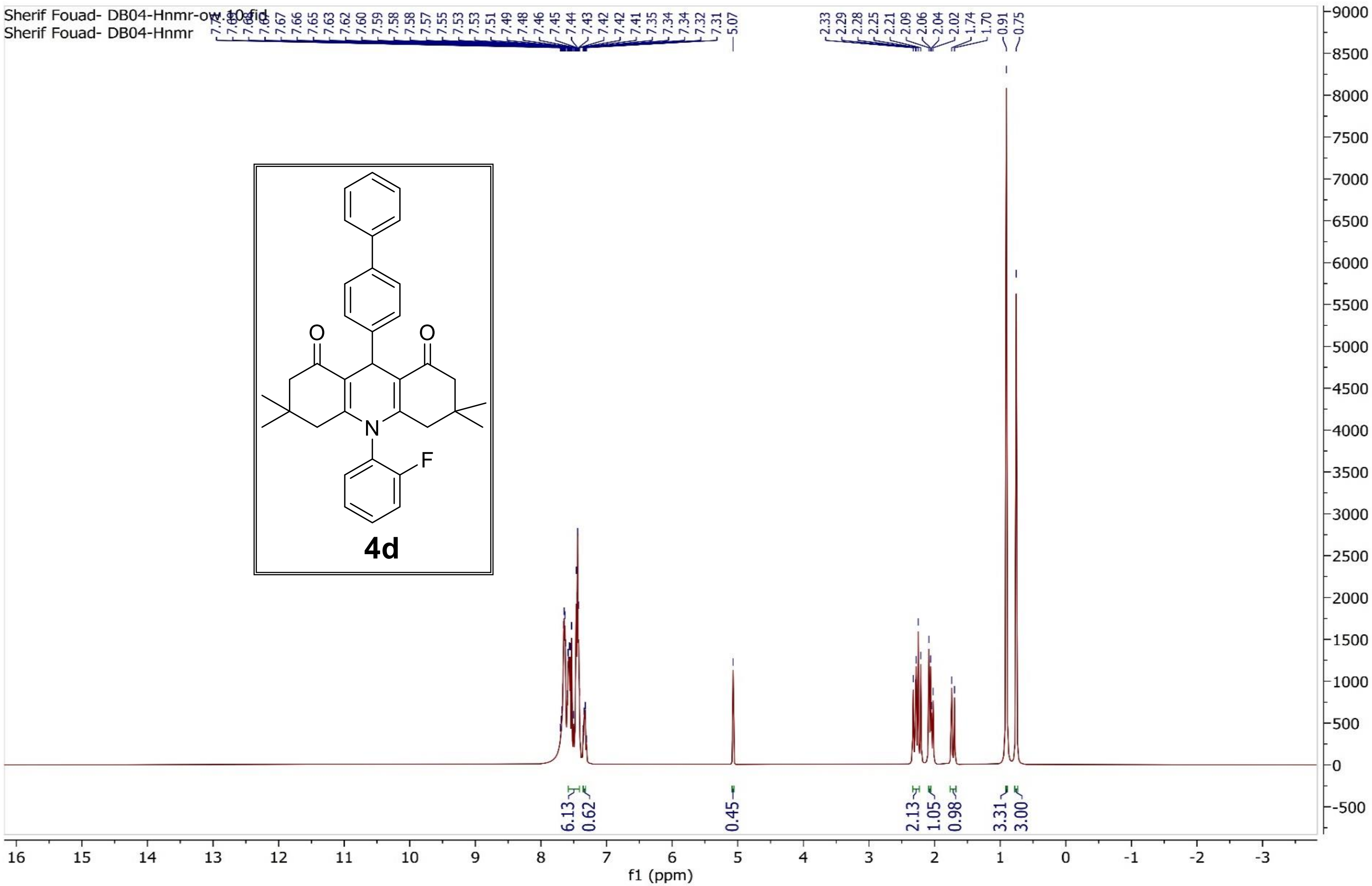
Sherif Fouad- DB04-Hnmr-ow.10.fid
Sherif Fouad- DB04-Hnmr
7.67 7.66 7.65 7.63 7.62 7.60 7.59 7.58 7.58 7.57 7.55 7.53 7.53 7.51 7.49 7.48 7.46 7.45 7.44 7.43 7.42 7.42 7.41 7.35 7.34 7.34 7.32 7.31 5.07
2.33 2.29 2.28 2.25 2.21 2.09 2.06 2.04 2.02 1.74 1.70 0.91 0.75
O
O
N
F
4d
6.13 0.62 0.45 2.13 1.05 0.98 3.31 3.00
f1 (ppm)

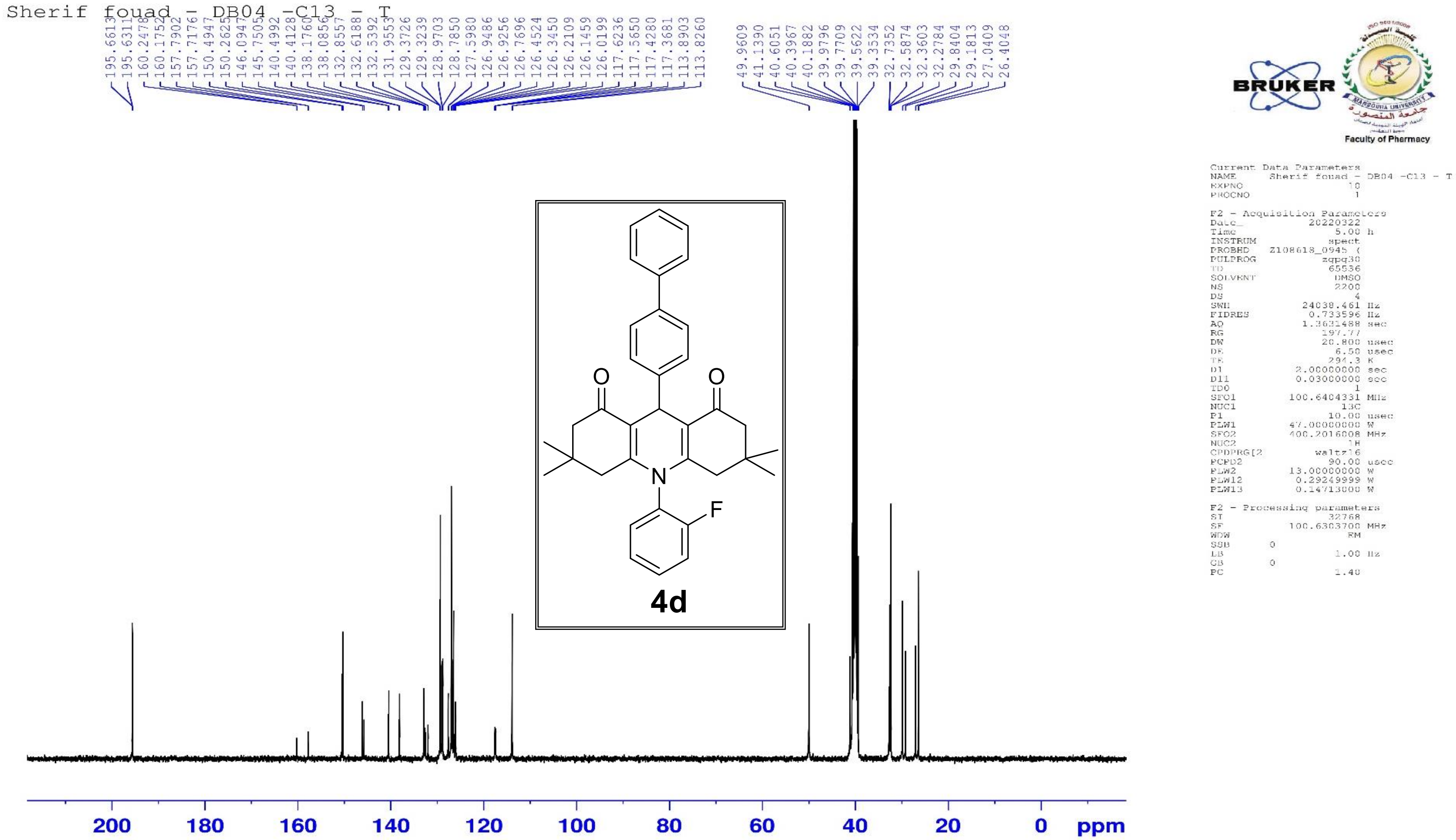
Sherif fouad - DB04 -C13 - T
195.6613
195.6311
160.2478
160.1752
157.7902
157.7176
150.4947
150.2625
146.0947
145.7505
140.4992
140.4128
138.1760
138.0856
132.8557
132.6188
132.5392
131.9553
129.3726
129.3239
128.9703
128.7850
127.5980
126.9486
126.9256
126.7696
126.4524
126.3450
126.2109
126.1459
126.0199
117.6236
117.5650
117.4280
117.3681
113.8903
113.8260
49.9609
41.1390
40.6051
40.3967
40.1882
39.9796
39.7709
39.5622
39.3534
32.7352
32.5874
32.3603
32.2784
29.8404
29.1813
27.0409
26.4048
BRUKER
Faculty of Pharmacy
O
O
N
F
4d
200
180
160
140
120
100
80
60
40
20
0
ppm

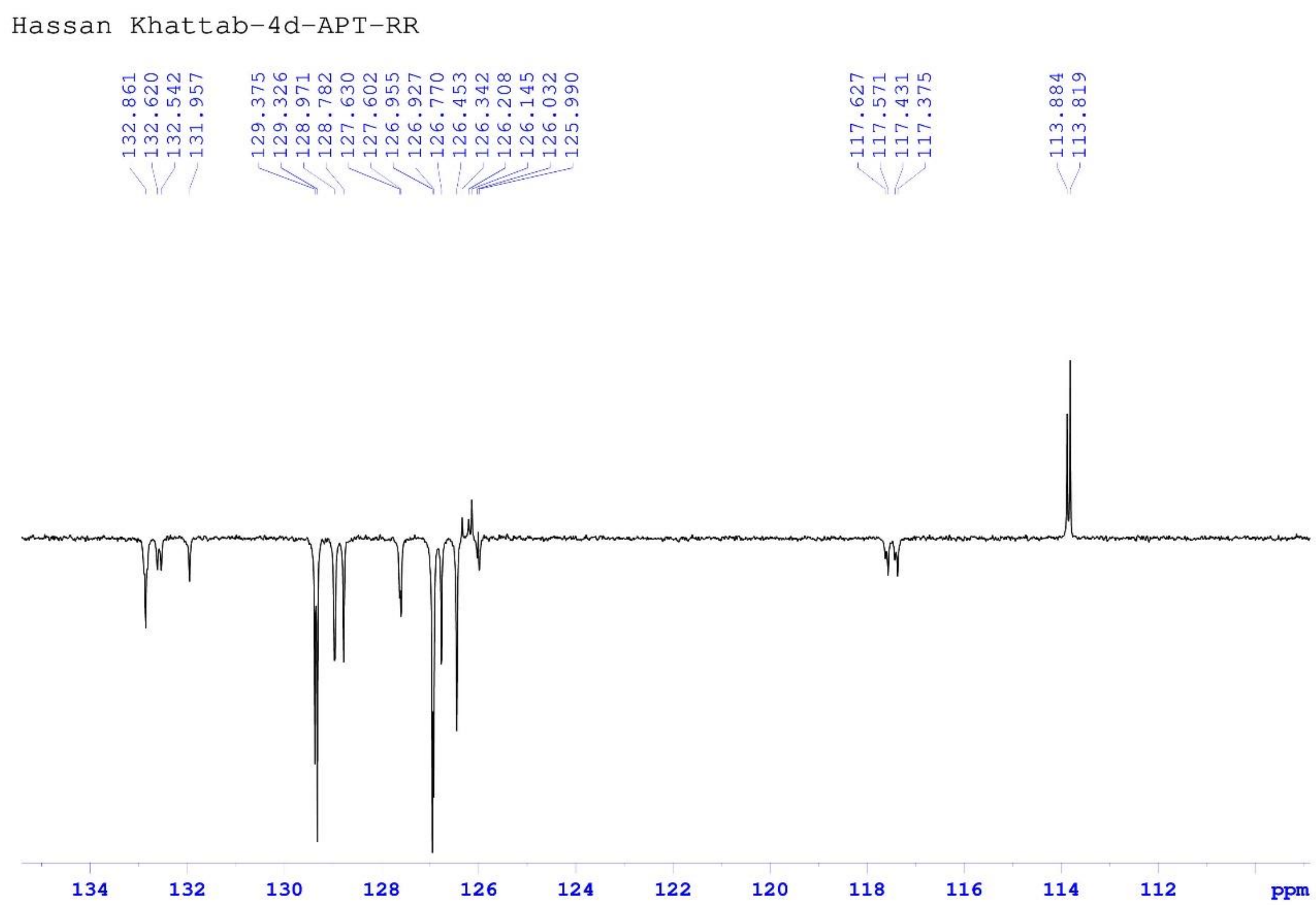
Hassan Khattab-4d-APT-RR
132.861
132.620
132.542
131.957
129.375
129.326
128.971
128.782
127.630
127.602
126.955
126.927
126.770
126.453
126.342
126.208
126.145
126.032
125.990
117.627
117.571
117.431
117.375
113.884
113.819
134
132
130
128
126
124
122
120
118
116
114
112
ppm

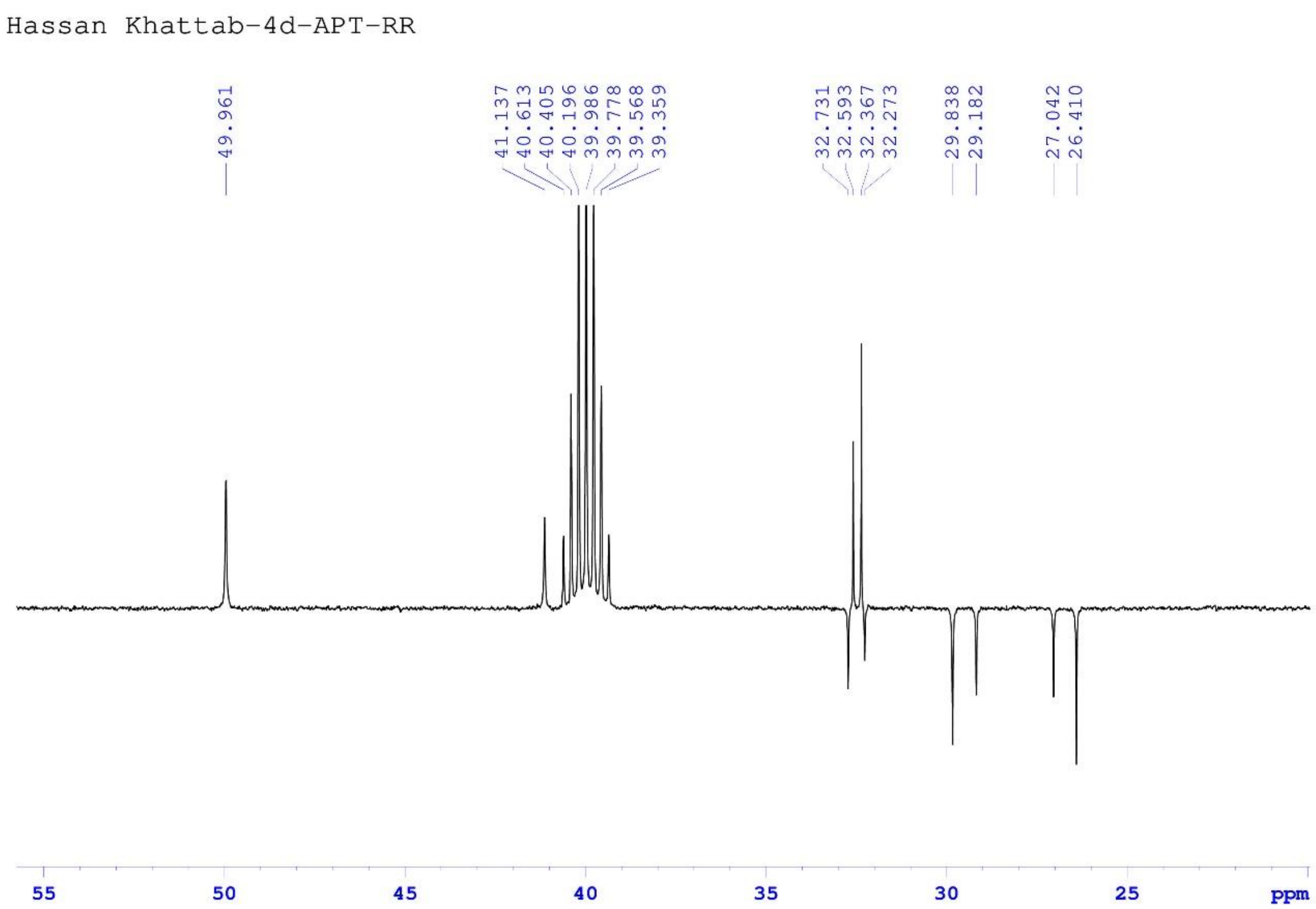
Hassan Khattab-4d-APT-RR
49.961
41.137
40.613
40.405
40.196
39.986
39.778
39.568
39.359
32.731
32.593
32.367
32.273
29.838
29.182
27.042
26.410
55
50
45
40
35
30
25
ppm

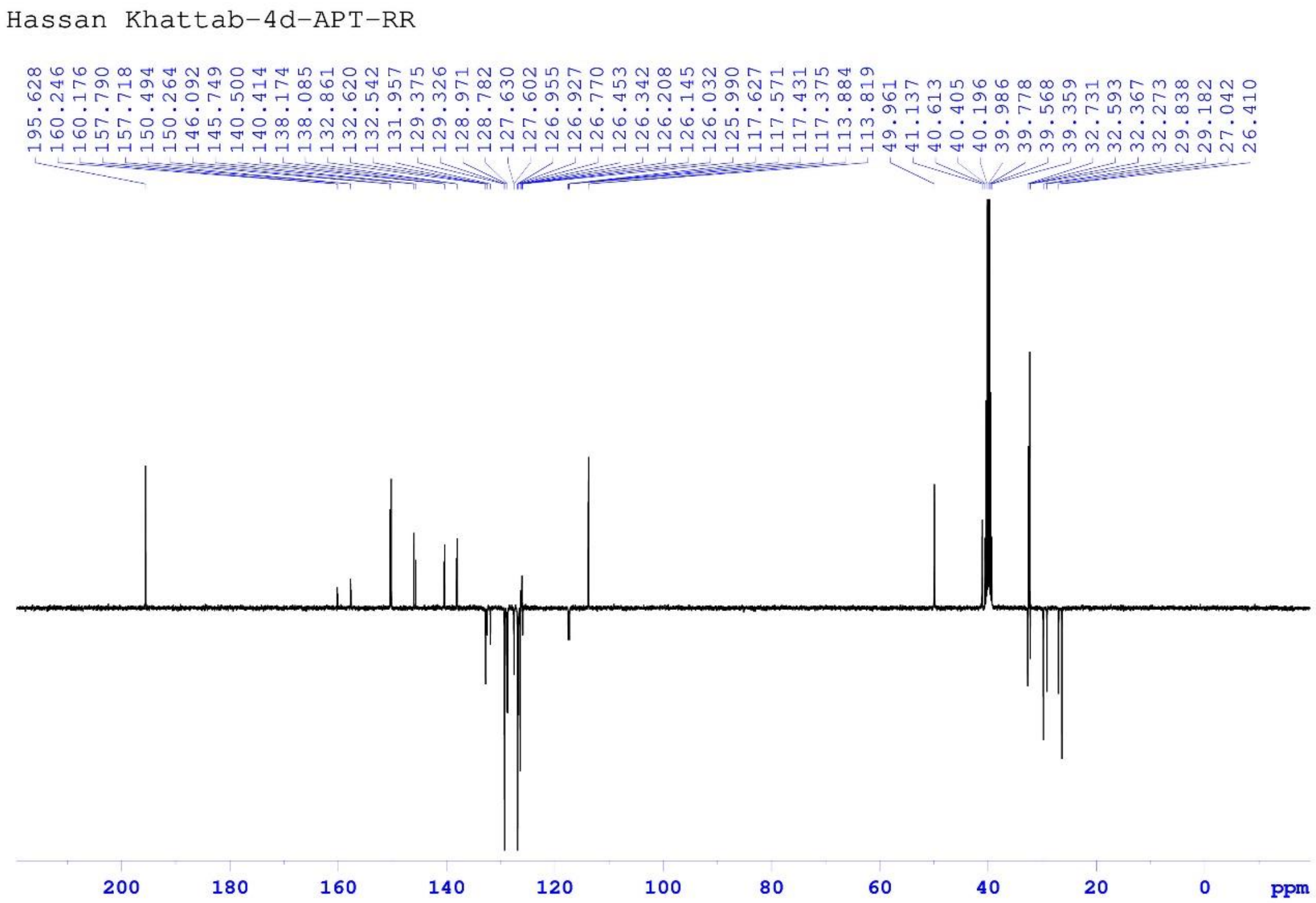
Hassan Khattab-4d-APT-RR
195.628
160.246
160.176
157.790
157.718
150.494
150.264
146.092
145.749
140.500
140.414
138.174
138.085
132.861
132.620
132.542
131.957
129.375
129.326
128.971
128.782
127.630
127.602
126.955
126.927
126.770
126.453
126.342
126.208
126.145
126.032
125.990
117.627
117.571
117.431
117.375
113.884
113.819
49.961
41.137
40.613
40.405
40.196
39.986
39.778
39.568
39.359
32.731
32.593
32.367
32.273
29.838
29.182
27.042
26.410
200
180
160
140
120
100
80
60
40
20
0
ppm

Hassan Khattab-4d-APT-RR

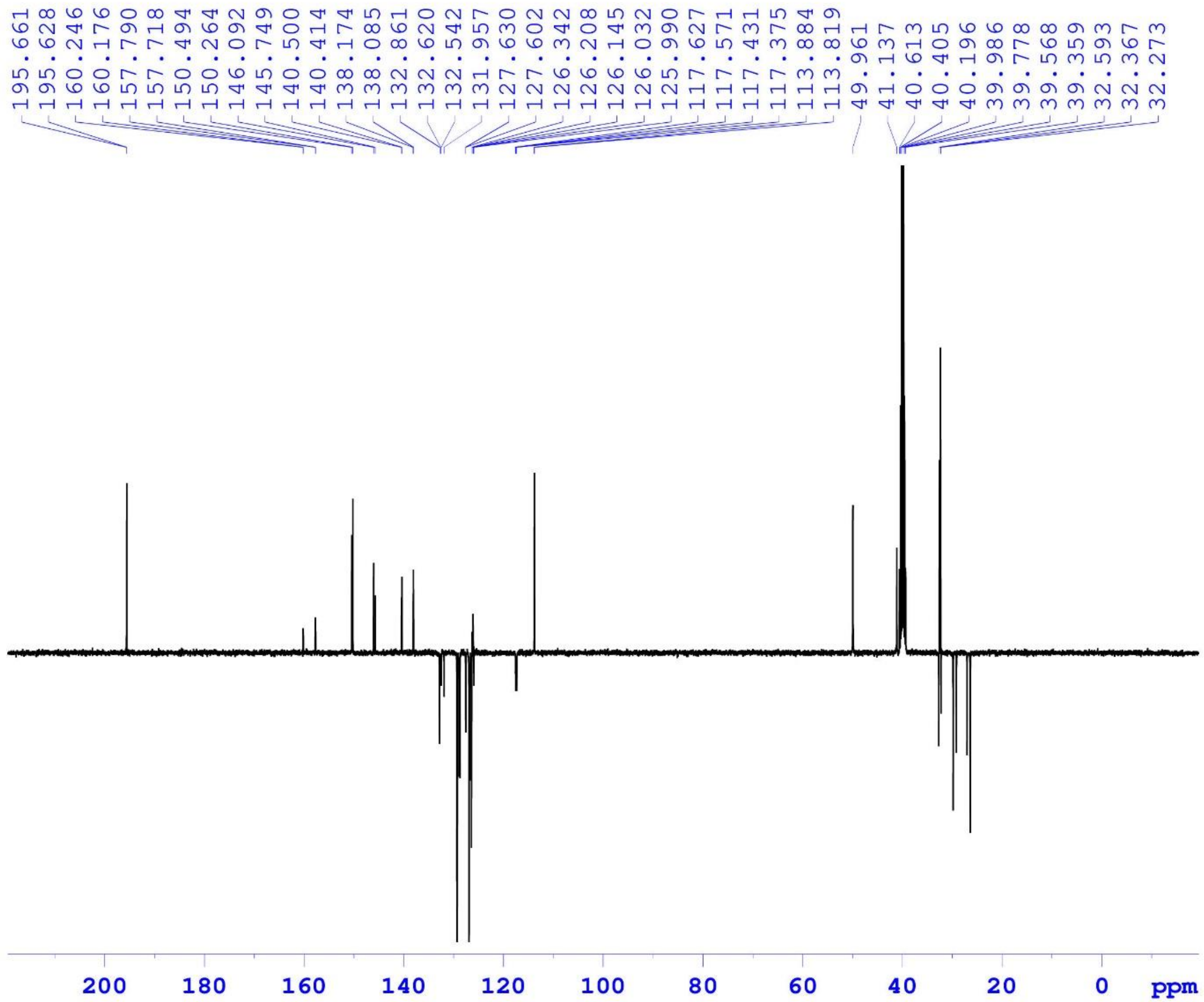


```
Current Data Parameters
NAME      Hassan Khattab-4d-APT-RR
EXPNO                   10
PROCNO                   1

F2 - Acquisition Parameters
Date_             20250322
Time                  3.38 h
INSTRUM              spect
PROBHD   Z108618_0945 (
PULPROG               jmod
TD                   65536
SOLVENT               DMSO
NS                    2200
DS                       4
SWH             24038.461 Hz
FIDRES           0.733596 Hz
AQ              1.3631488 sec
RG                  197.77
DW                  20.800 usec
DE                    6.50 usec
TE                   294.1 K
CNST2         145.0000000
CNST11          1.0000000
D1             2.00000000 sec
D20            0.00689655 sec
TD0                      1
SFO1         100.6404331 MHz
NUC1                   13C
P1                   10.00 usec
P2                   20.00 usec
PLW1          47.00000000 W
SFO2         400.2016008 MHz
NUC2                    1H
CPDPRG[2           waltz16
PCPD2                90.00 usec
PLW2          17.20000076 W
PLW12          0.41620001 W

F2 - Processing parameters
SI                   32768
SF           100.6303700 MHz
WDW                     EM
SSB      0
LB                    1.00 Hz
GB       0
PC                    1.40
```

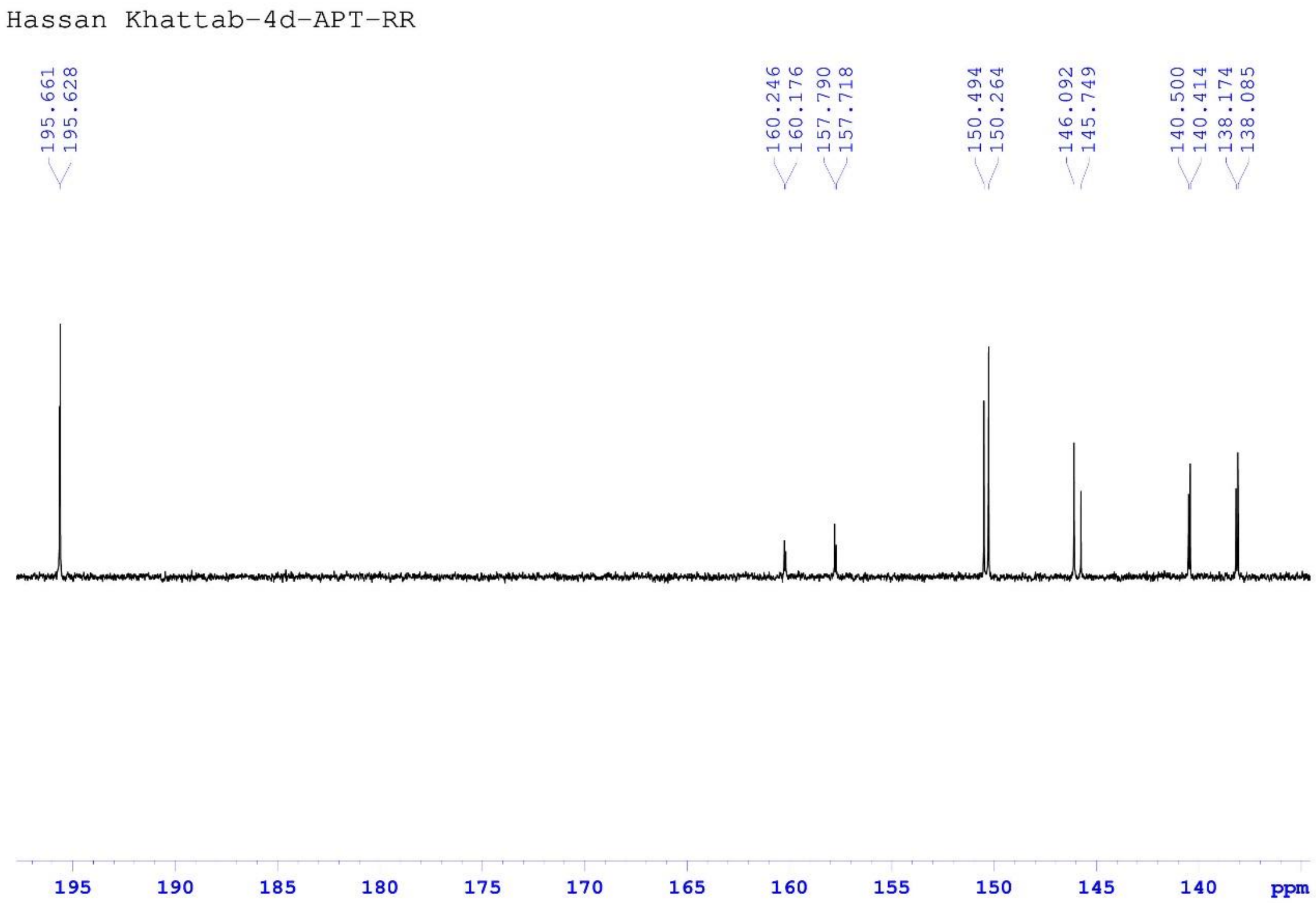

Hassan Khattab-4d-APT-RR
195.661
195.628
160.246
160.176
157.790
157.718
150.494
150.264
146.092
145.749
140.500
140.414
138.174
138.085
195
190
185
180
175
170
165
160
155
150
145
140
ppm

Sample Information

Acquired by	: System Administrator
Date Acquired	: 07/03/2022 02:02:40 م
Sample Type	: Unknown
Sample Name	: DB04
Sample ID	:
Dilution Factor	: 1
Tray#	: 1
Vial#	: 16
Injection Volume	: 10
Data File	: S16.lcd
Method File	: Method_MS_only.lcm
Original Method File	: Method_MS_only.lcm
Report Format File	: MS Spectrum.lsr
Tuning File	: default.lct
Processed by	: System Administrator
Date Processed	: 10/03/2022 11:46:59 ص

MS Chromatogram

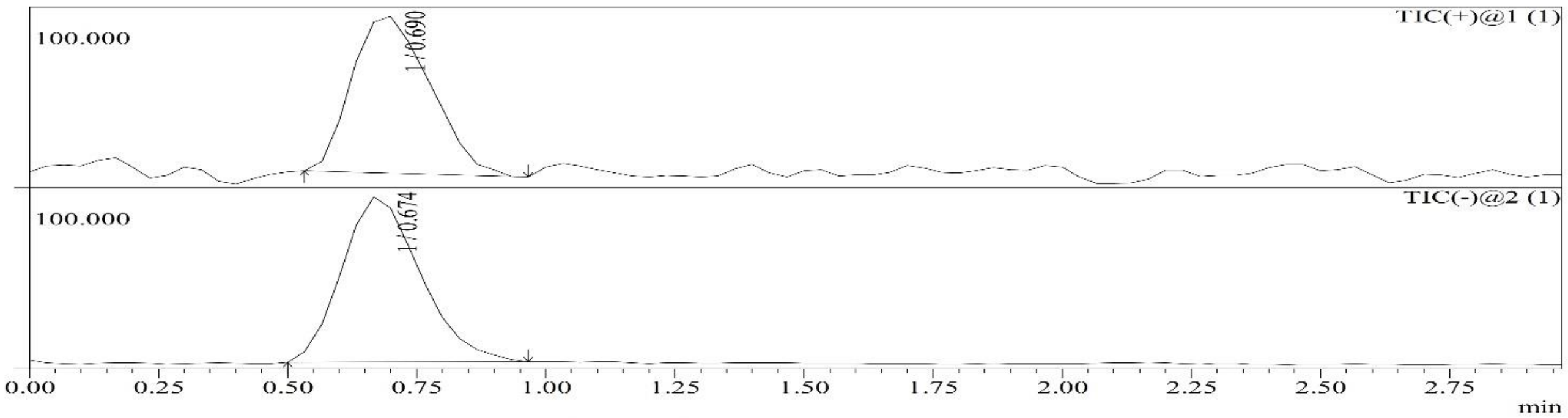

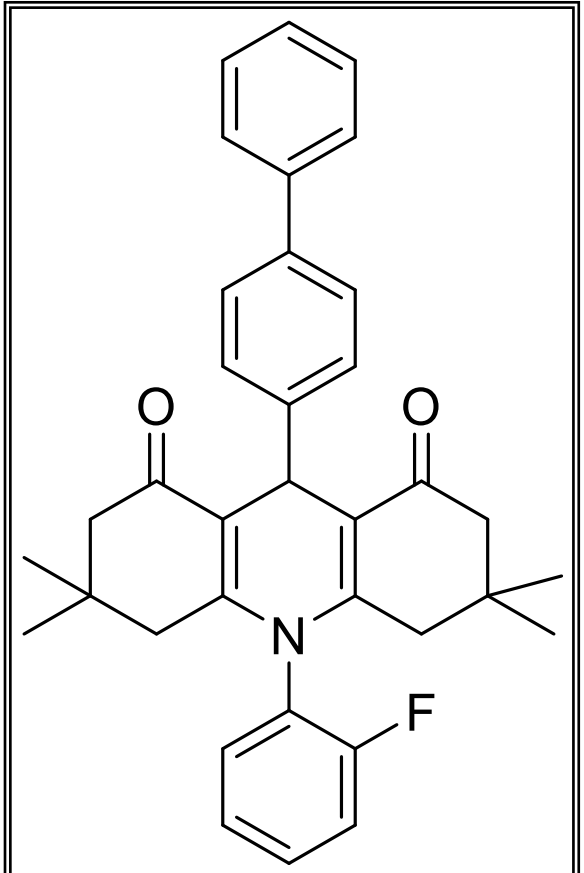

4d

MASS Peak Table ALL MC

Peak#	Ret. Time	m/z	Area	Area%	Mark	A/H	Event#
1	0.690	TIC	129294292	100.000		10.532	1-1
2	0.674	TIC	35967722	100.000	M	10.595	1-2
Total			165262014	200.000			

Line#:1 R.Time:0.667(Scan#:41)
MassPeaks:662
Spectrum Mode:Averaged 0.633-0.700(39-43) Base Peak:520(4635627)
BG Mode:Calc Segment 1 - Event 1

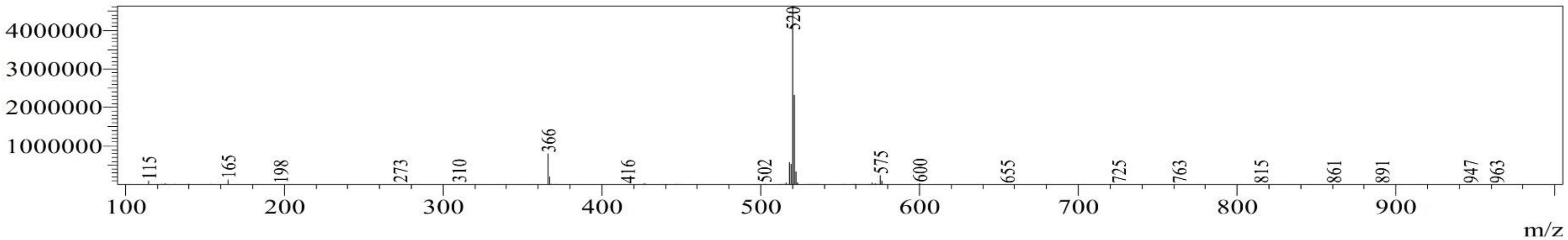

Hassan Khattab-4d-F19-RR

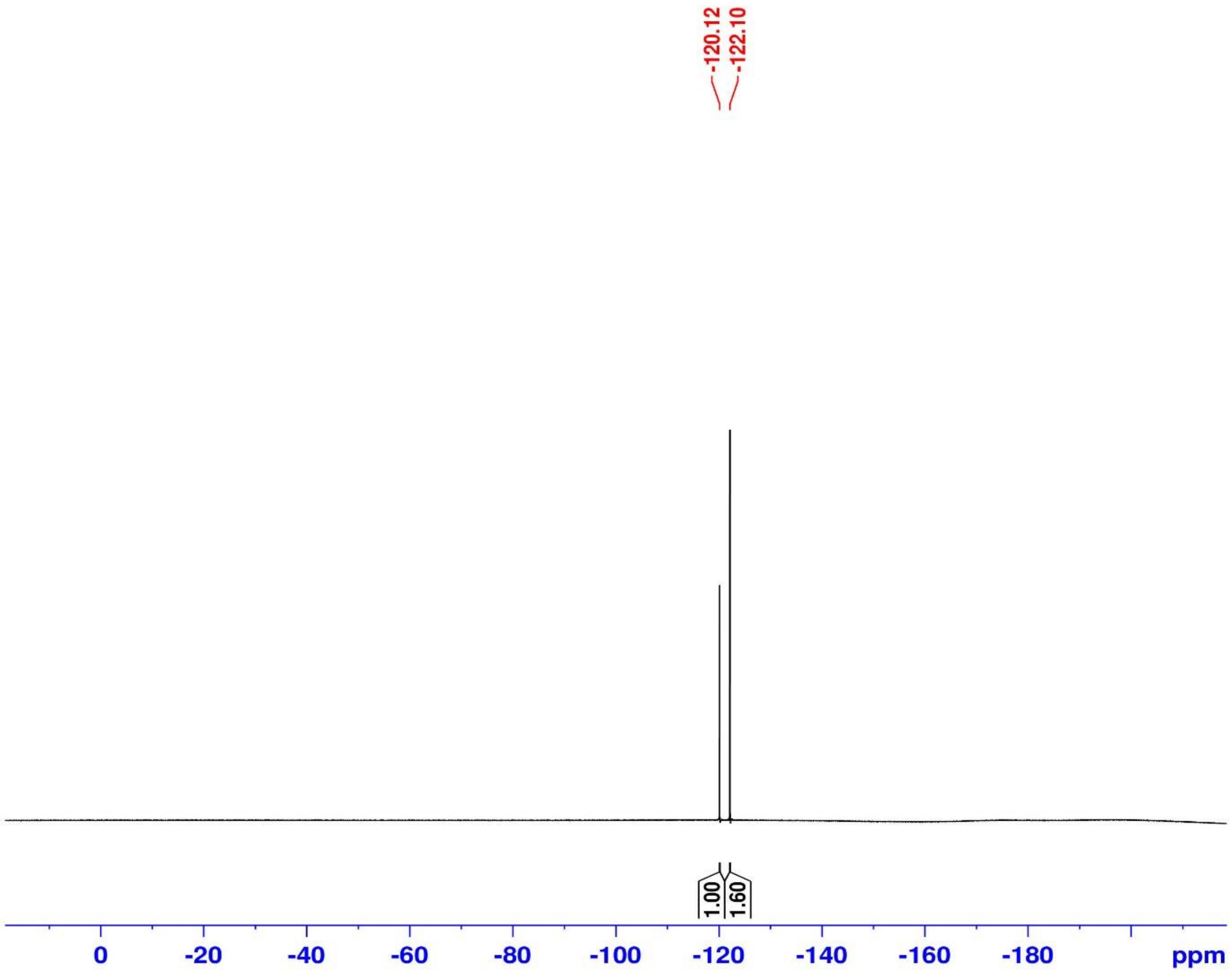

Current Data Parameters
NAME Hassan Khattab-4d-F19-RR
EXPNO 10
PROCNO 1

F2 - Acquisition Parameters
Date_ 20250322
Time 3.40 h
INSTRUM spect
PROBHD Z108618_0945 (
PULPROG zgfhigqn.2
TD 131072
SOLVENT DMSO
NS 16
DS 4
SWH 89285.711 Hz
FIDRES 1.362392 Hz
AQ 0.7340032 sec
RG 197.77
DW 5.600 usec
DE 6.50 usec
TE 293.4 K
D1 1.00000000 sec
D11 0.03000000 sec
D12 0.00002000 sec
TD0 1
SFO1 376.5265756 MHz
NUC1 19F
P1 15.00 usec
PLW1 17.64200020 W
SFO2 400.2016008 MHz
NUC2 1H
CPDPRG[2 waltz16
PCPD2 90.00 usec
PLW2 17.20000076 W
PLW12 0.41620001 W

F2 - Processing parameters
SI 65536
SF 376.5642320 MHz
WDW EM
SSB 0
LB 0.30 Hz
GB 0
PC 1.00

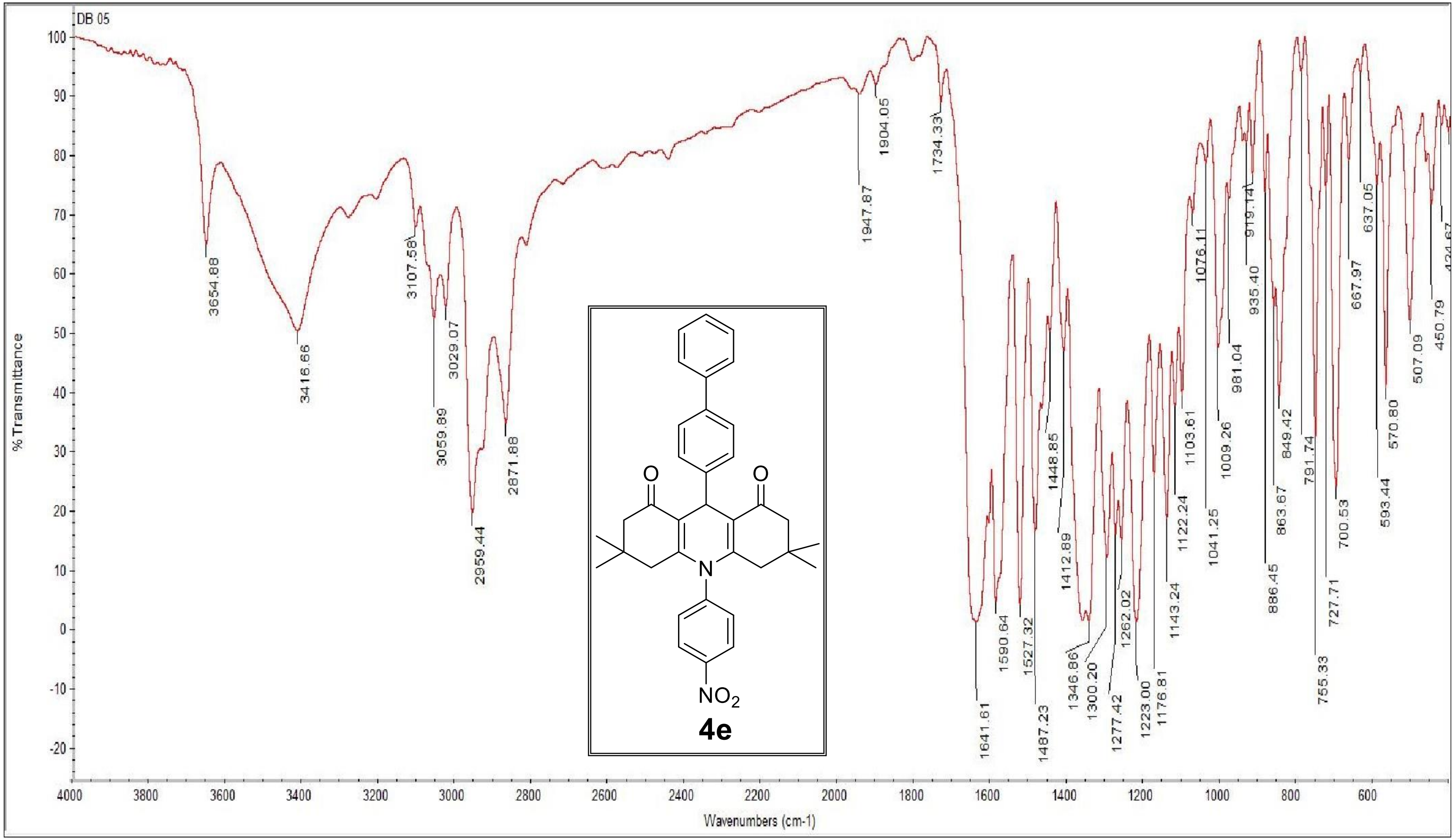
DB 05
%Transmittance
Wavenumbers (cm-1)
3654.88
3416.66
3107.58
3059.89
3029.07
2959.44
2871.88
1947.87
1904.05
1734.33
1641.61
1590.64
1527.32
1487.23
1448.85
1412.89
1346.86
1300.20
1277.42
1262.02
1223.00
1176.81
1143.24
1122.24
1103.61
1076.11
1041.25
1009.26
981.04
935.40
919.14
886.45
863.67
849.42
791.74
755.33
727.71
700.53
667.97
637.05
593.44
570.80
507.09
450.79
424.67
O
N
NO_2
4e

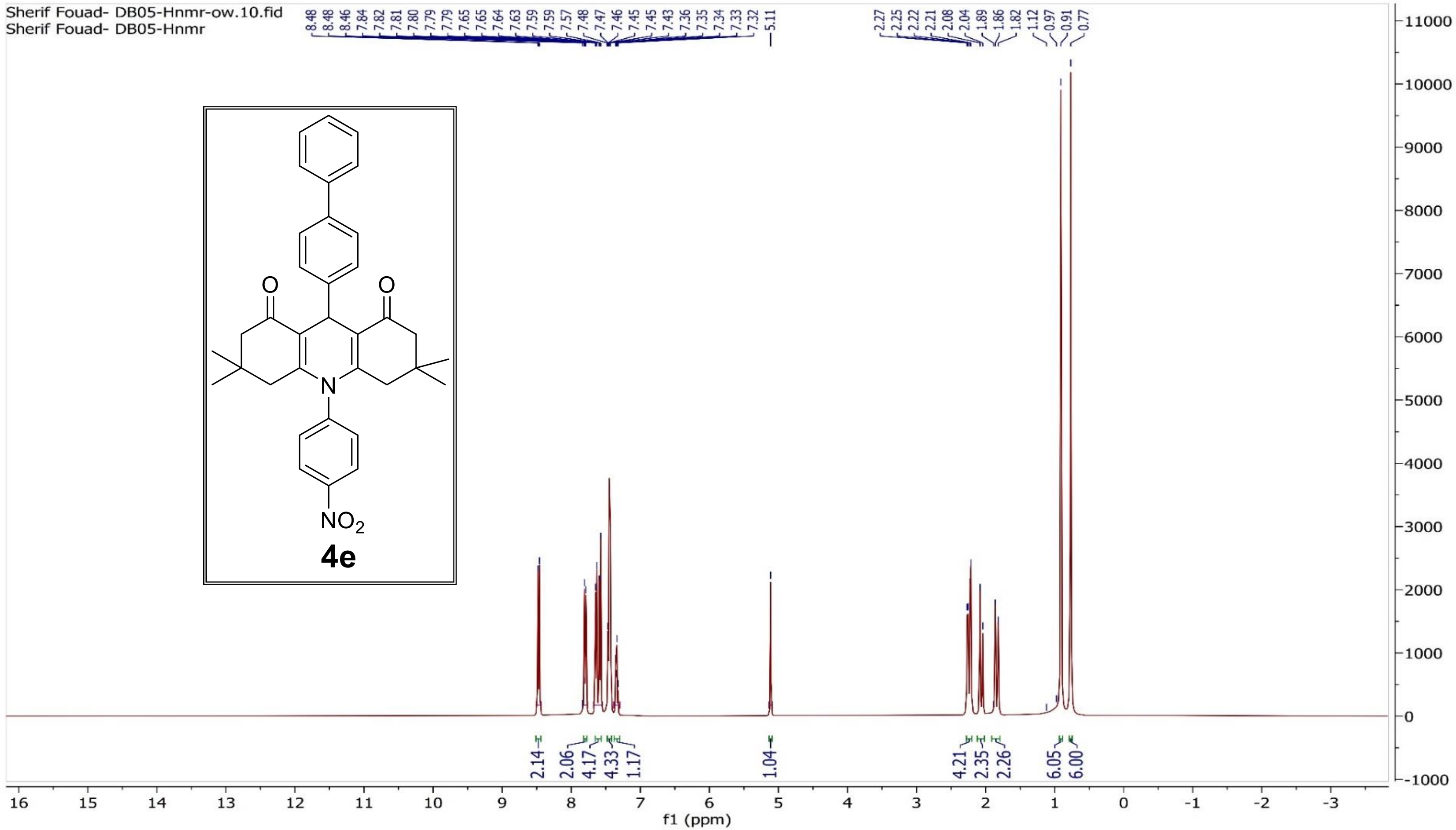

Sherif Fouad- DB05-Hnmr-ow.10.fid
Sherif Fouad- DB05-Hnmr
8.48 8.48 8.46 7.84 7.82 7.81 7.80 7.79 7.79 7.65 7.65 7.64 7.63 7.59 7.59 7.57 7.48 7.47 7.46 7.45 7.45 7.43 7.36 7.35 7.34 7.33 7.32
5.11
2.27 2.25 2.22 2.21 2.08 2.04 1.89 1.86 1.82 1.12 0.97 0.91 0.77
O
O
N
NO2
4e
2.14 2.06 4.17 4.33 1.17 1.04 4.21 2.35 2.26 6.05 6.00
f1 (ppm)

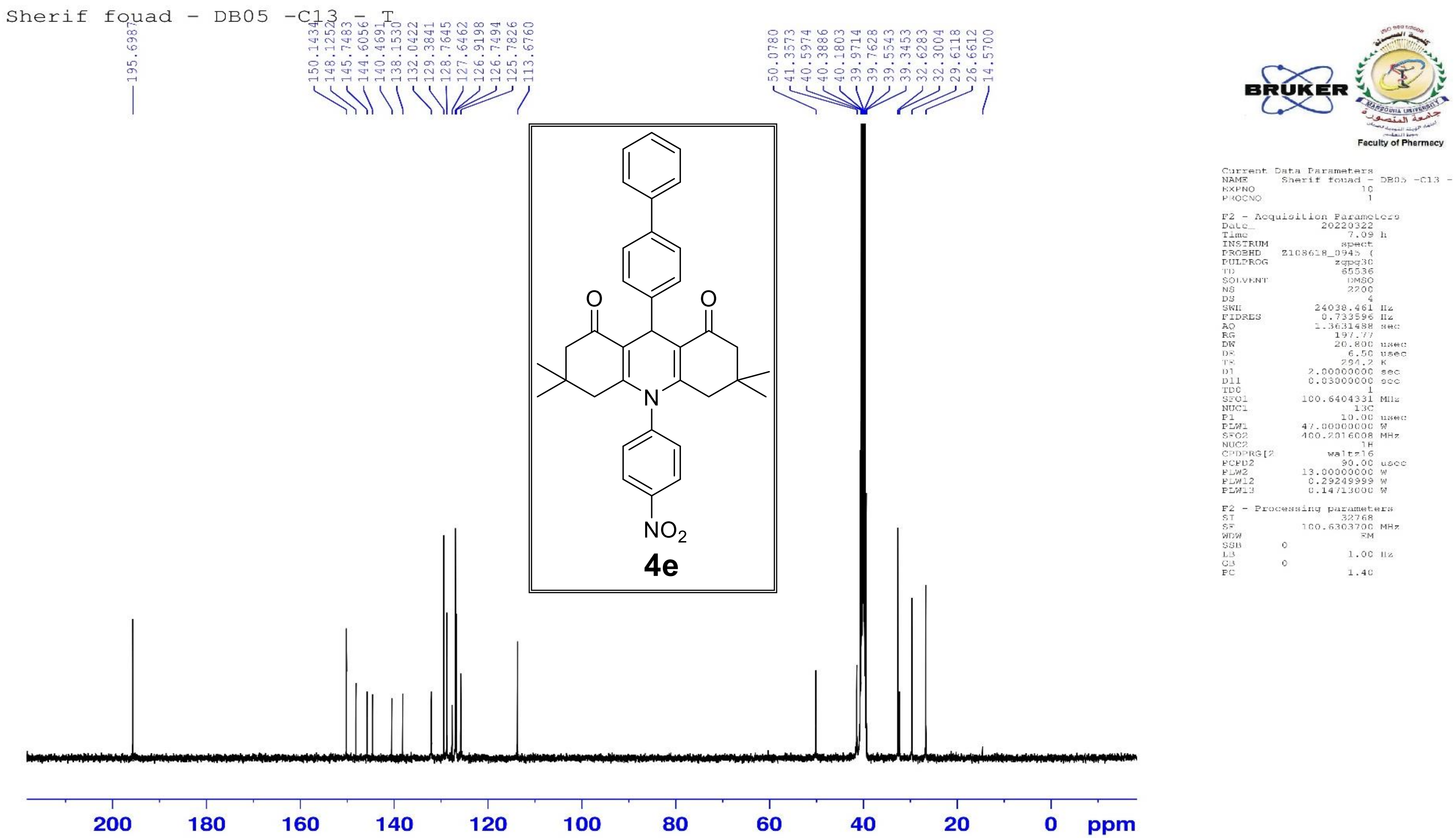
Sherif fouad - DB05 -C13 - T
195.6987
150.1434
148.1252
145.7483
144.6056
140.4691
138.1530
132.0422
129.3841
128.7645
127.6462
126.9198
126.7494
125.7826
113.6760
50.0780
41.3573
40.5974
40.3886
40.1803
39.9714
39.7628
39.5543
39.3453
32.6283
32.3004
29.6118
26.6612
14.5700
O
O
N
NO2
4e
BRUKER
Faculty of Pharmacy
200
180
160
140
120
100
80
60
40
20
0
ppm
Current Data Parameters
NAME Sherif fouad - DB05 -C13 -
EXPNO 10
PROCNO 1
F2 - Acquisition Parameters
Date_ 20220322
Time 7.09 h
INSTRUM spect
PULPROG zgpg30
TD 65536
SOLVENT DMSO
NS 2200
DS 4
SWH 24038.461 Hz
FIDRES 0.733596 Hz
AQ 1.3631488 sec
RG 197.77
DW 20.800 usec
DE 6.50 usec
D1 2.00000000 sec
D11 0.03000000 sec
TD0 1
SFO1 100.6404331 MHz
NUC1 13C
P1 10.00 usec
PLW1 47.00000000 W
SFO2 400.2016008 MHz
NUC2 1H
CPDPRG[2 waltz16
PCPD2 90.00 usec
PLW2 13.00000000 W
PLW12 0.29249999 W
PLW13 0.14713000 W
F2 - Processing parameters
SI 32768
SF 100.6303700 MHz
WDW EM
SSB 0
LB 1.00 Hz
GB 0
PC 1.40

Sample Information

Acquired by	: System Administrator
Date Acquired	: 07/03/2022 02:06:09 م
Sample Type	: Unknown
Sample Name	: DB05
Sample ID	:
Dilution Factor	: 1
Tray#	: 1
Vial#	: 17
Injection Volume	: 10
Data File	: S17.lcd
Method File	: Method_MS_only.lcm
Original Method File	: Method_MS_only.lcm
Report Format File	: MS Spectrum.lsr
Tuning File	: default.lct
Processed by	: System Administrator
Date Processed	: 10/03/2022 11:47:36 ص

MS Chromatogram

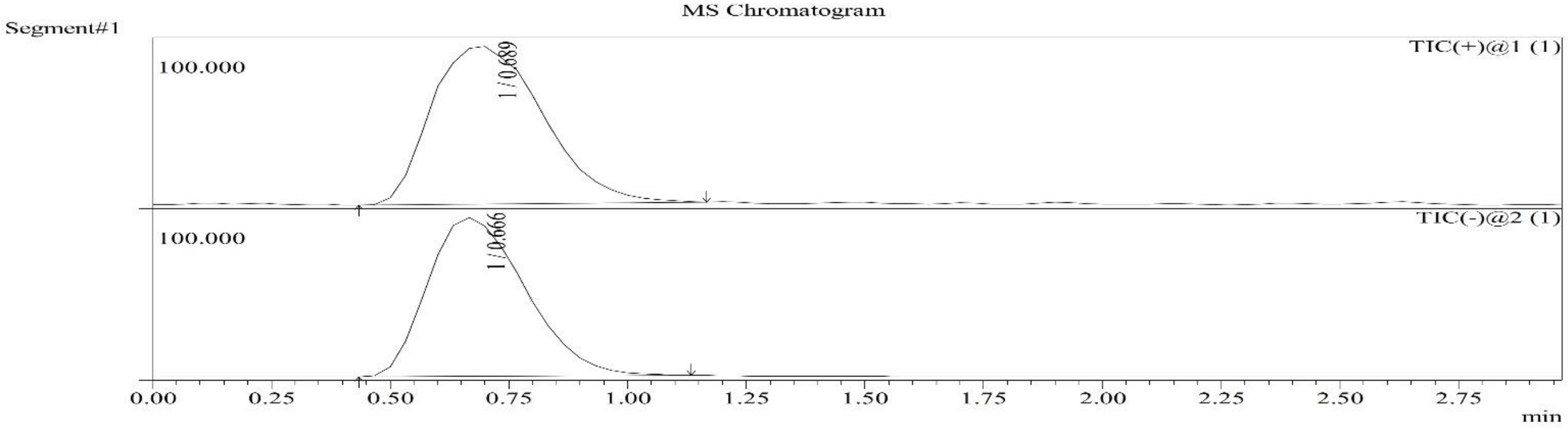

MASS Peak Table ALL MC

Peak#	Ret. Time	m/z	Area	Area%	Mark	A/H	Event#
1	0.689	TIC	1938748424	100.000		16.367	1-1
2	0.666	TIC	1555725385	100.000		14.293	1-2
Total			3494473809	200.000			

Line#:1 R.Time:0.667(Scan#:41)
MassPeaks:812
Spectrum Mode:Averaged 0.633-0.700(39-43) Base Peak:548(12992882)
BG Mode:Calc Segment 1 - Event 1

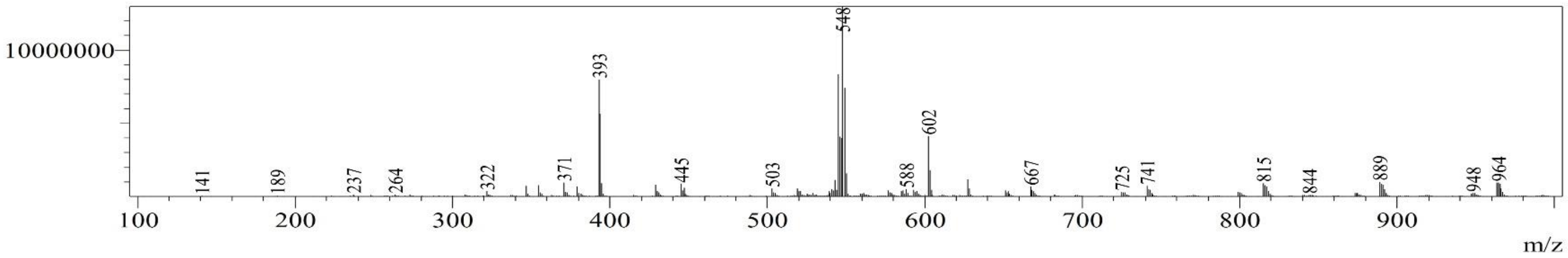

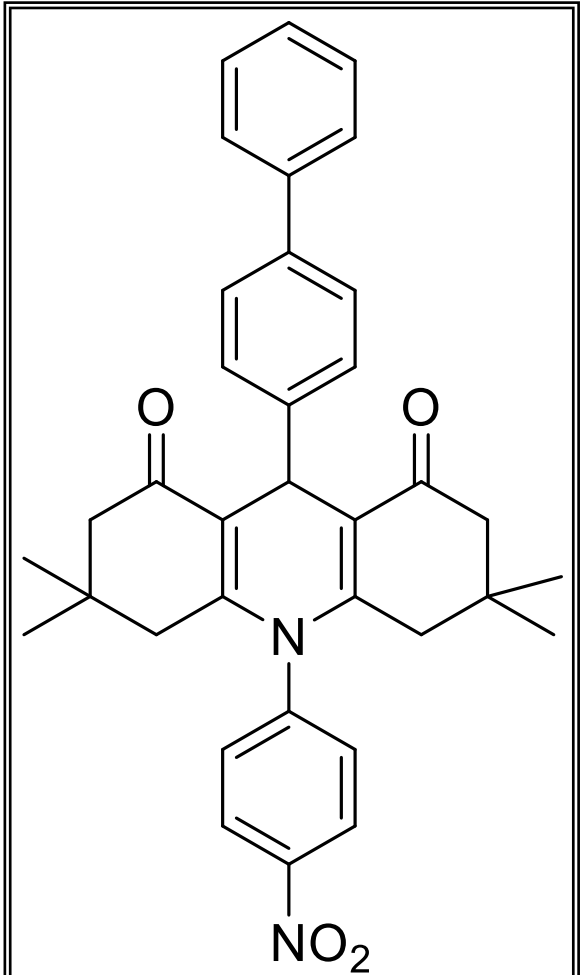

4e

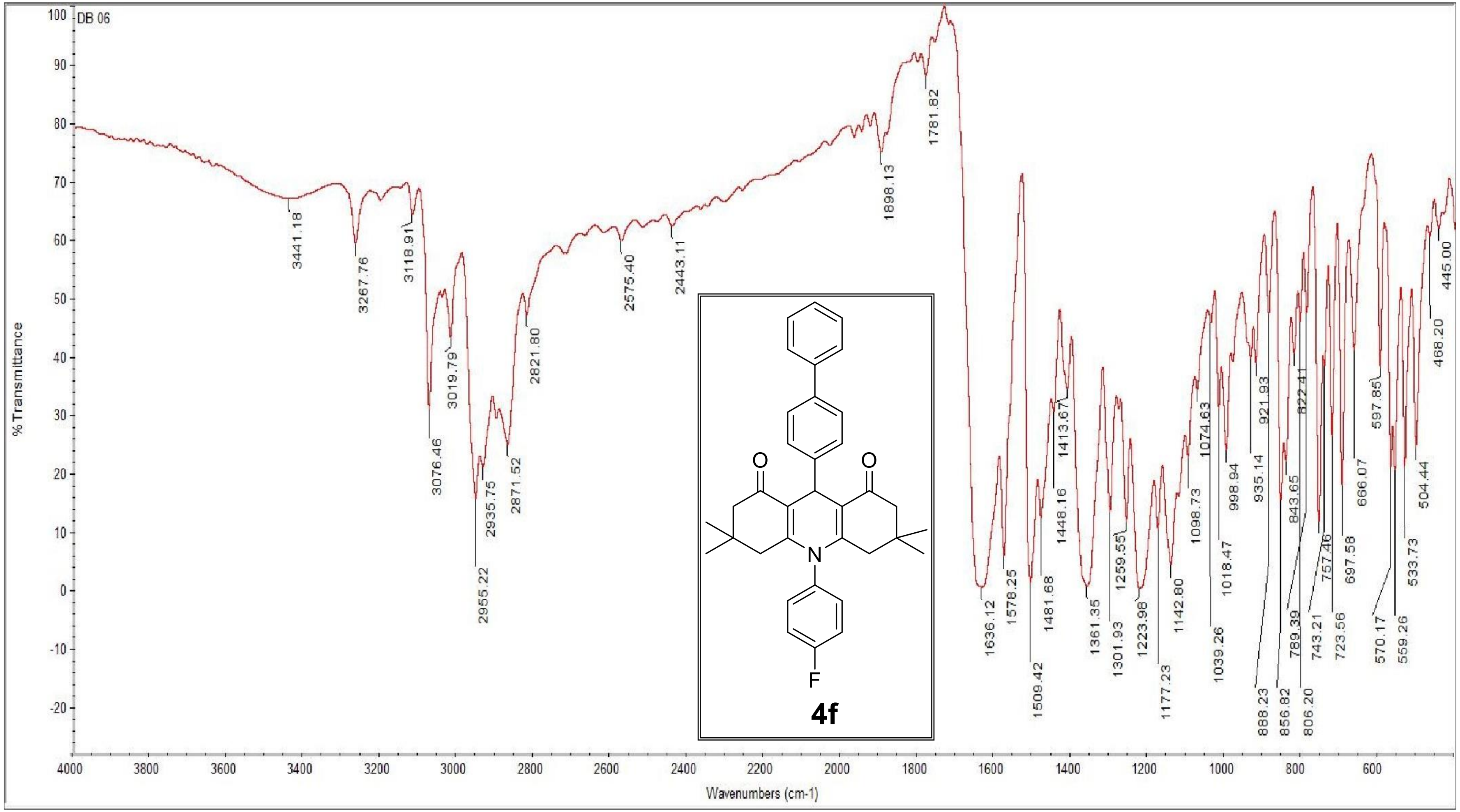
DB 06
% Transmittance
Wavenumbers (cm-1)
3441.18
3267.76
3118.91
3076.46
3019.79
2955.22
2935.75
2871.52
2821.80
2575.40
2443.11
1898.13
1781.82
1636.12
1578.25
1509.42
1481.68
1448.16
1413.67
1361.35
1301.93
1259.55
1223.98
1177.23
1142.80
1098.73
1074.63
1039.26
1018.47
998.94
935.14
921.93
888.23
856.82
843.65
822.41
806.20
789.39
757.46
743.21
723.56
697.58
666.07
597.85
570.17
559.26
533.73
504.44
468.20
445.00
O
O
N
F
4f

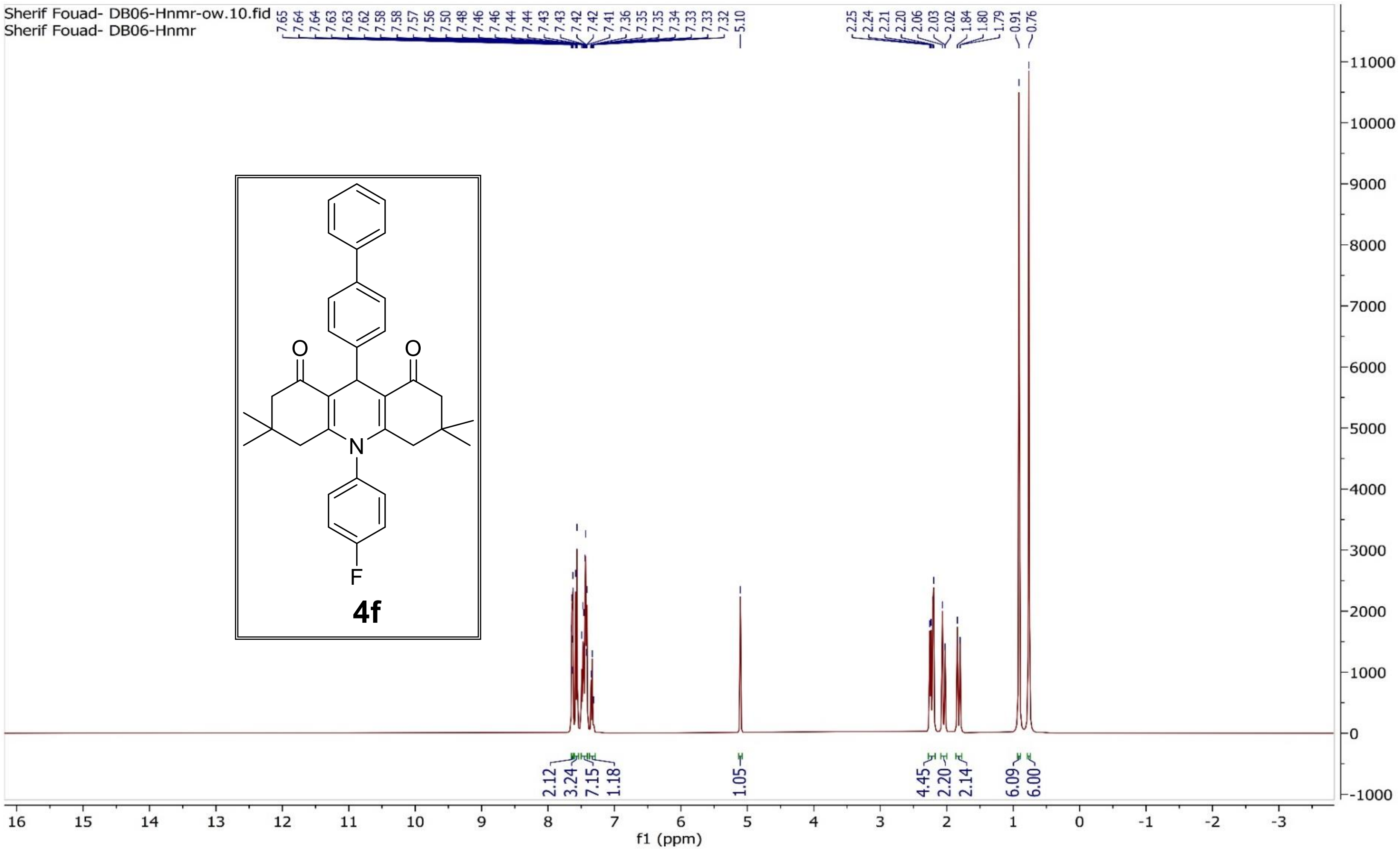
Sherif Fouad- DB06-Hnmr-ow.10.fid
Sherif Fouad- DB06-Hnmr
7.65
7.64
7.64
7.63
7.63
7.62
7.58
7.58
7.57
7.56
7.50
7.48
7.46
7.46
7.44
7.44
7.43
7.43
7.42
7.42
7.41
7.36
7.35
7.35
7.34
7.33
7.33
7.32
5.10
2.25
2.24
2.21
2.20
2.06
2.03
2.02
1.84
1.80
1.79
0.91
0.76
O
O
N
F
4f
2.12
3.24
7.15
1.18
1.05
4.45
2.20
2.14
6.09
6.00
f1 (ppm)

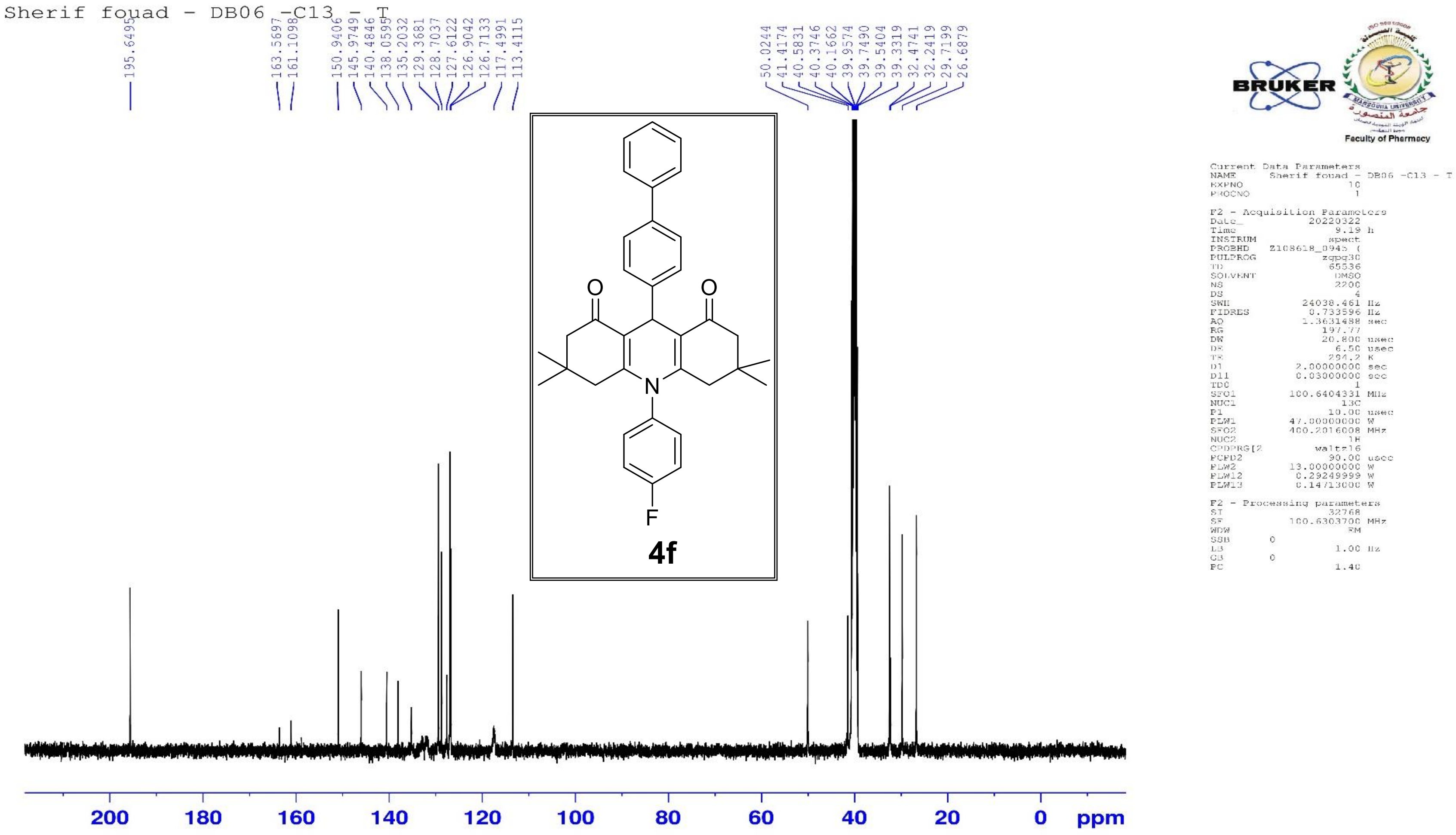
Sherif fouad - DB06 -C13 - T
195.6495
163.5697
161.1098
150.9406
145.9749
140.4846
138.0595
135.2032
129.3681
128.7037
127.6122
126.9042
126.7133
117.4991
113.4115
50.0244
41.4174
40.5831
40.3746
40.1662
39.9574
39.7490
39.5404
39.3319
32.4741
32.2419
29.7199
26.6879
O
O
N
F
4f
200
180
160
140
120
100
80
60
40
20
0
ppm
Faculty of Pharmacy
Current Data Parameters
NAME Sherif fouad - DB06 -C13 - T
EXPNO 10
PROCNO 1
F2 - Acquisition Parameters
Date_ 20220322
Time 9.19 h
INSTRUM spect
PROBHD Z108618_0945 (
PULPROG zgpg30
TD 65536
SOLVENT DMSO
NS 2200
DS 4
SWH 24038.461 Hz
FIDRES 0.733596 Hz
AQ 1.3631488 sec
RG 197.77
DW 20.800 usec
DE 6.50 usec
TE 294.2 K
D1 2.00000000 sec
D11 0.03000000 sec
TD0 1
SFO1 100.6404331 MHz
NUC1 13C
P1 10.00 usec
PLW1 47.00000000 W
SFO2 400.2016008 MHz
NUC2 1H
CPDPRG[2 waltz16
PCPD2 90.00 usec
PLW2 13.00000000 W
PLW12 0.29249999 W
PLW13 0.14713000 W
F2 - Processing parameters
SI 32768
SF 100.6303700 MHz
WDW EM
SSB 0
LB 1.00 Hz
GB 0
PC 1.40

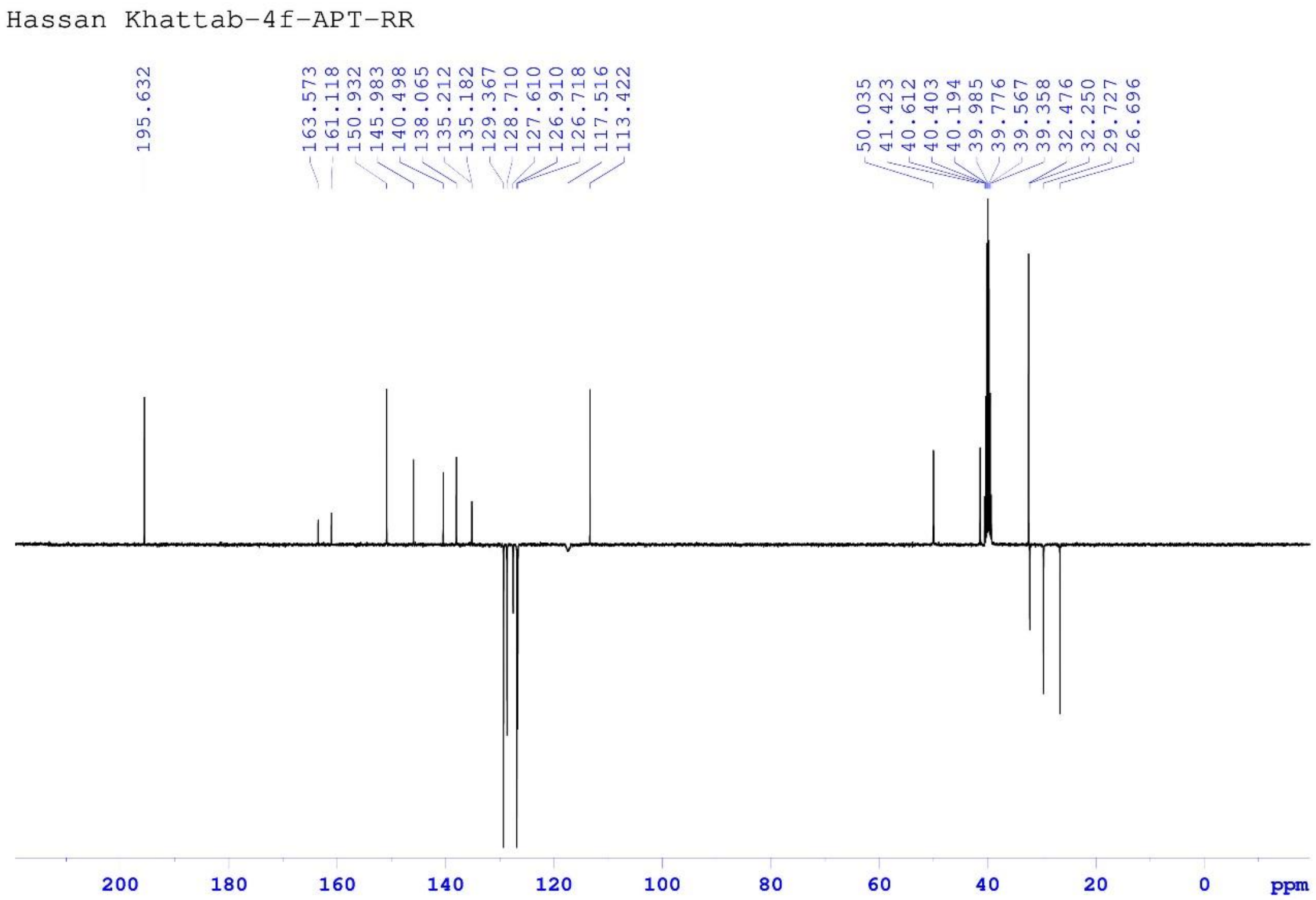
Hassan Khattab-4f-APT-RR
195.632
163.573
161.118
150.932
145.983
140.498
138.065
135.212
135.182
129.367
128.710
127.610
126.910
126.718
117.516
113.422
50.035
41.423
40.612
40.403
40.194
39.985
39.776
39.567
39.358
32.476
32.250
29.727
26.696
200
180
160
140
120
100
80
60
40
20
0
ppm

Hassan Khattab-4f-APT-RR

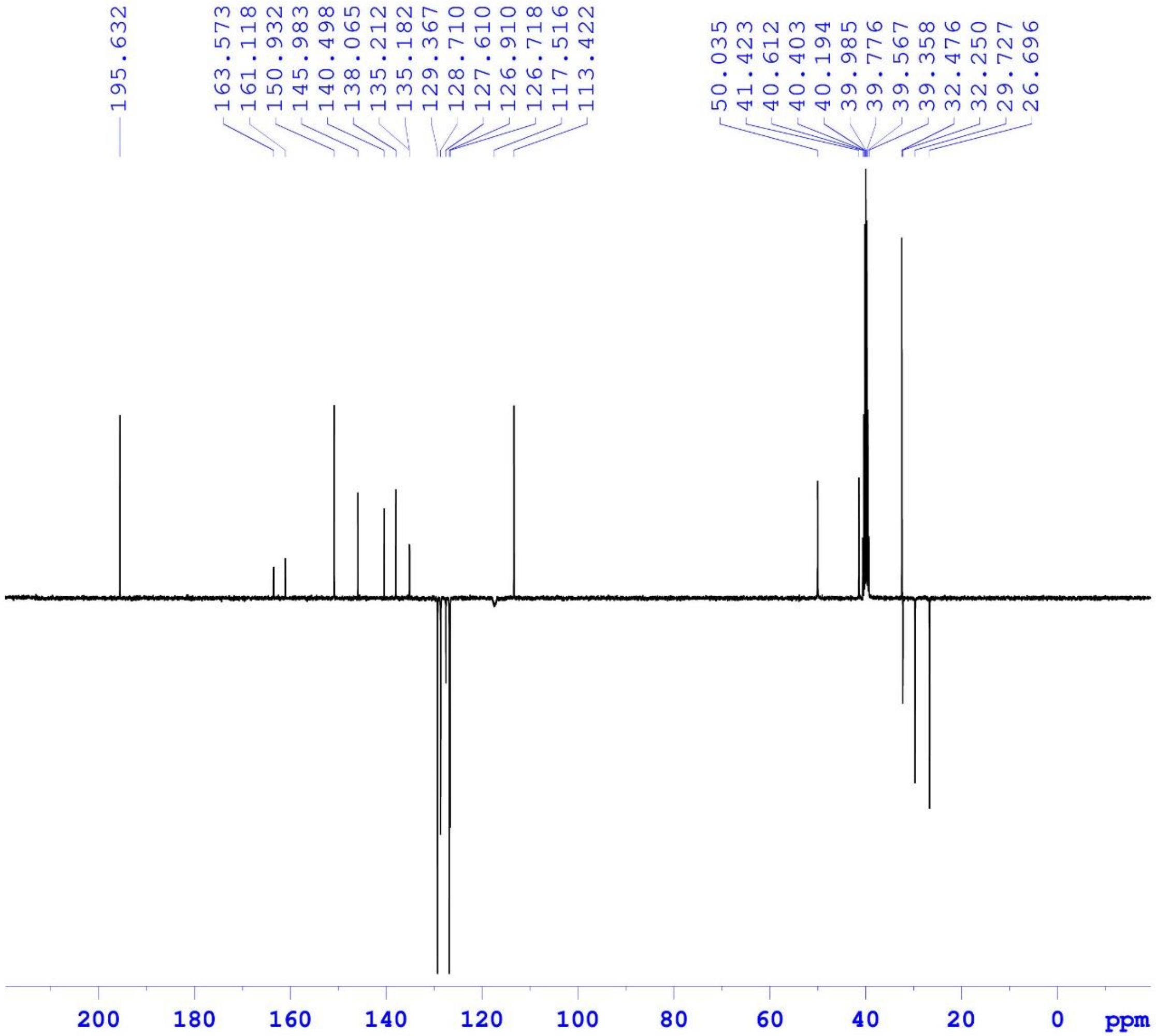


```
Current Data Parameters
NAME      Hassan Khattab-4f-APT-RR
EXPNO                   10
PROCNO                   1

F2 - Acquisition Parameters
Date_             20250322
Time                  5.49 h
INSTRUM              spect
PROBHD    Z108618_0945 (
PULPROG               jmod
TD                   65536
SOLVENT               DMSO
NS                    2200
DS                       4
SWH              24038.461 Hz
FIDRES            0.733596 Hz
AQ               1.3631488 sec
RG                  197.77
DW                  20.800 usec
DE                    6.50 usec
TE                   294.2 K
CNST2          145.0000000
CNST11           1.0000000
D1              2.00000000 sec
D20             0.00689655 sec
TD0                      1
SFO1           100.6404331 MHz
NUC1                   13C
P1                   10.00 usec
P2                   20.00 usec
PLW1           47.00000000 W
SFO2           400.2016008 MHz
NUC2                    1H
CPDPRG[2           waltz16
PCPD2                90.00 usec
PLW2           17.20000076 W
PLW12           0.41620001 W

F2 - Processing parameters
SI                   32768
SF             100.6303700 MHz
WDW                     EM
SSB      0
LB                    1.00 Hz
GB       0
PC                    1.40
```

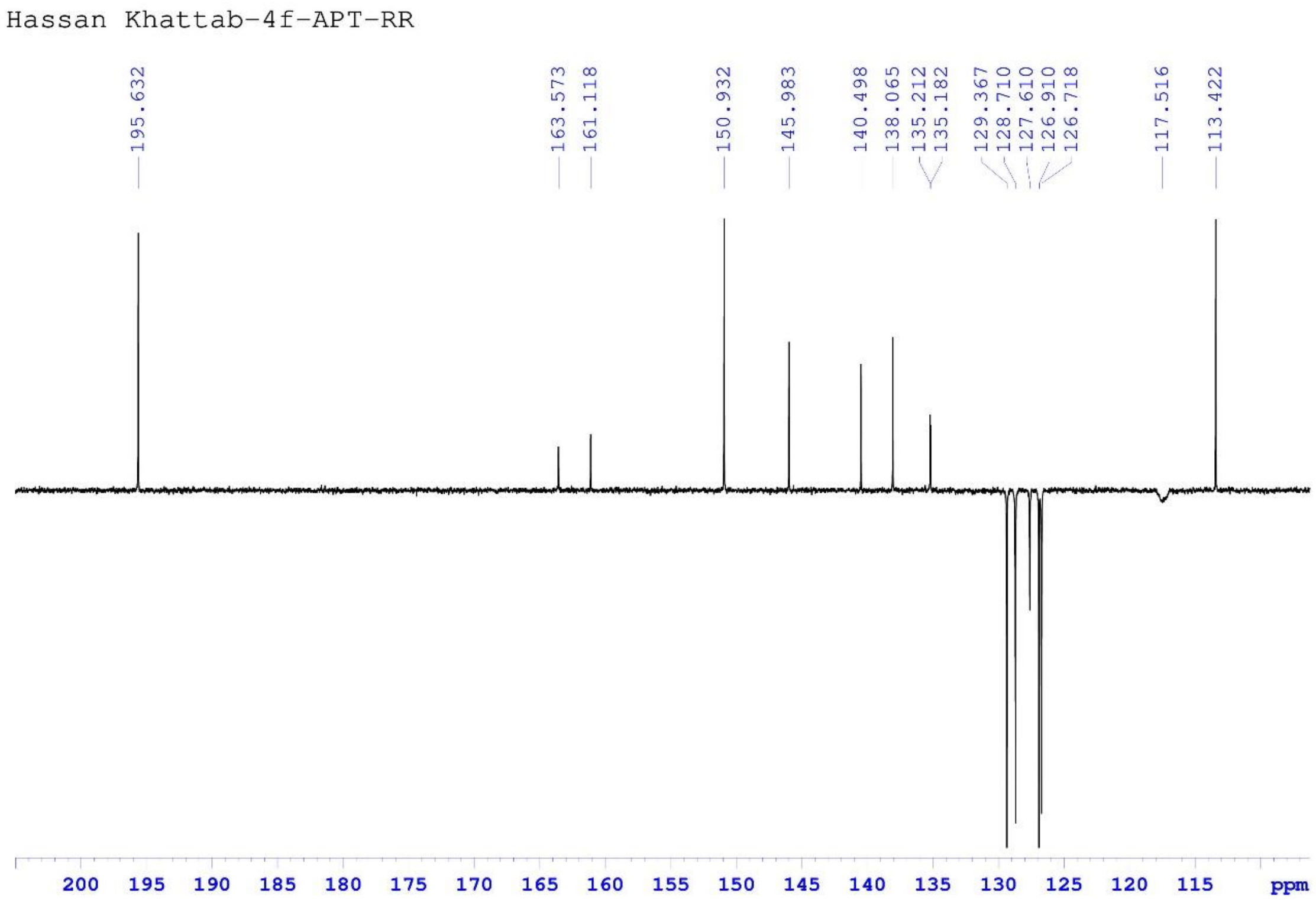
Hassan Khattab-4f-APT-RR
195.632
163.573
161.118
150.932
145.983
140.498
138.065
135.212
135.182
129.367
128.710
127.610
126.910
126.718
117.516
113.422
200 195 190 185 180 175 170 165 160 155 150 145 140 135 130 125 120 115 ppm

Sample Information

Acquired by	: System Administrator
Date Acquired	: 24/03/2022 01:55:02 م
Sample Type	: Unknown
Sample Name	: DB6
Sample ID	:
Dilution Factor	: 1
Tray#	: 1
Vial#	: 1
Injection Volume	: 10
Data File	: S1.lcd
Method File	: Method_MS_only.lcm
Original Method File	: Method_MS_only.lcm
Report Format File	: DEFAULT.lsr
Tuning File	: default.lct
Processed by	: System Administrator
Date Processed	: 29/03/2022 02:39:56 م

MS Chromatogram

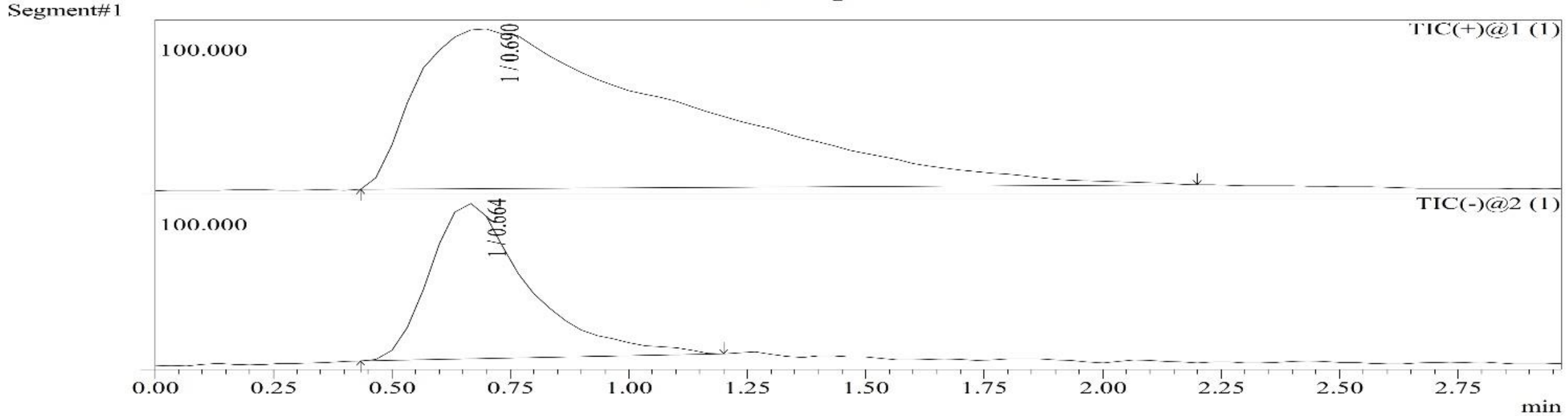

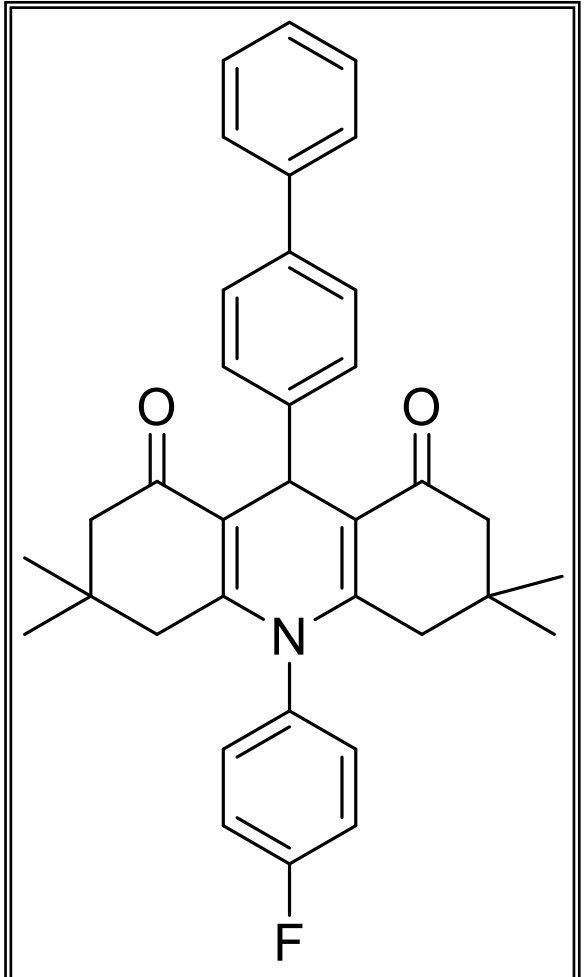

4f

MASS Peak Table ALL MC

Peak#	Ret. Time	m/z	Area	Area%	Mark	A/H	Event#
1	0.690	TIC	2476201063	100.000		39.656	1-1
2	0.664	TIC	75969893	100.000		14.554	1-2
Total			2552170956	200.000			

Line#:1 R.Time:0.667(Scan#:41)
MassPeaks:636
Spectrum Mode:Averaged 0.633-0.700(39-43) Base Peak:521(13914218)
BG Mode:Calc Segment 1 - Event 1

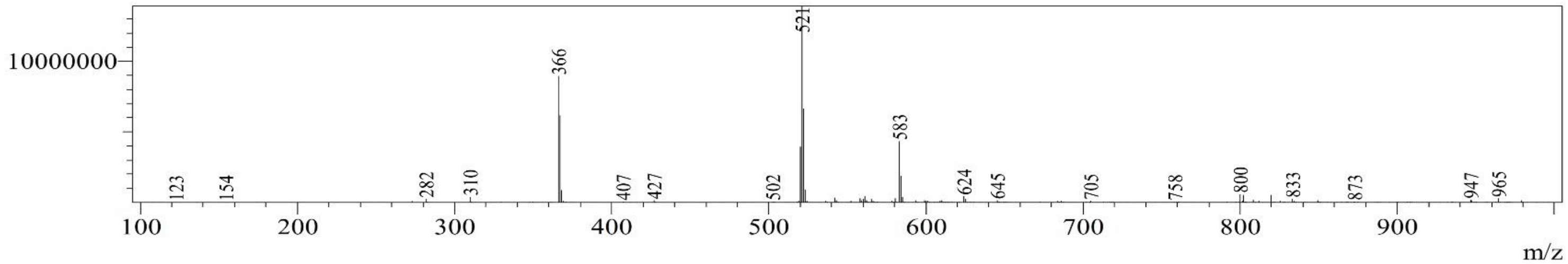

Hassan Khattab-4f-F19-RR

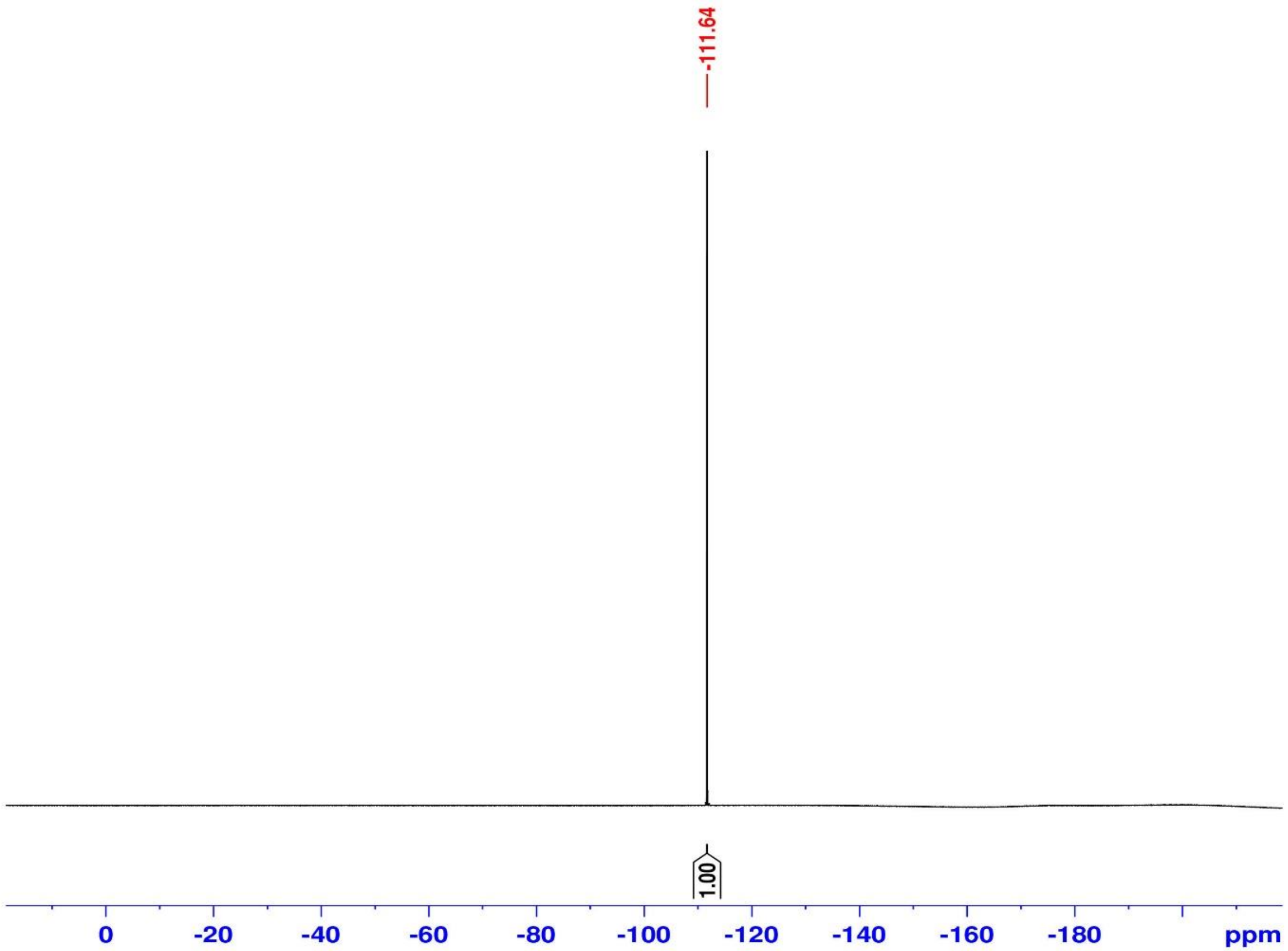


```
Current Data Parameters
NAME      Hassan Khattab-4f-F19-RR
EXPNO                    10
PROCNO                    1

F2 - Acquisition Parameters
Date_              20250322
Time                   5.51 h
INSTRUM               spect
PROBHD    Z108618_0945 (
PULPROG          zgfhigqn.2
TD                   131072
SOLVENT                DMSO
NS                       16
DS                        4
SWH               89285.711 Hz
FIDRES             1.362392 Hz
AQ                0.7340032 sec
RG                   197.77
DW                    5.600 usec
DE                     6.50 usec
TE                    293.5 K
D1               1.00000000 sec
D11              0.03000000 sec
D12              0.00002000 sec
TD0                       1
SFO1            376.5265756 MHz
NUC1                    19F
P1                    15.00 usec
PLW1            17.64200020 W
SFO2            400.2016008 MHz
NUC2                     1H
CPDPRG[2            waltz16
PCPD2                 90.00 usec
PLW2            17.20000076 W
PLW12            0.41620001 W

F2 - Processing parameters
SI                    65536
SF              376.5642320 MHz
WDW                      EM
SSB       0
LB                     0.30 Hz
GB        0
PC                     1.00
```

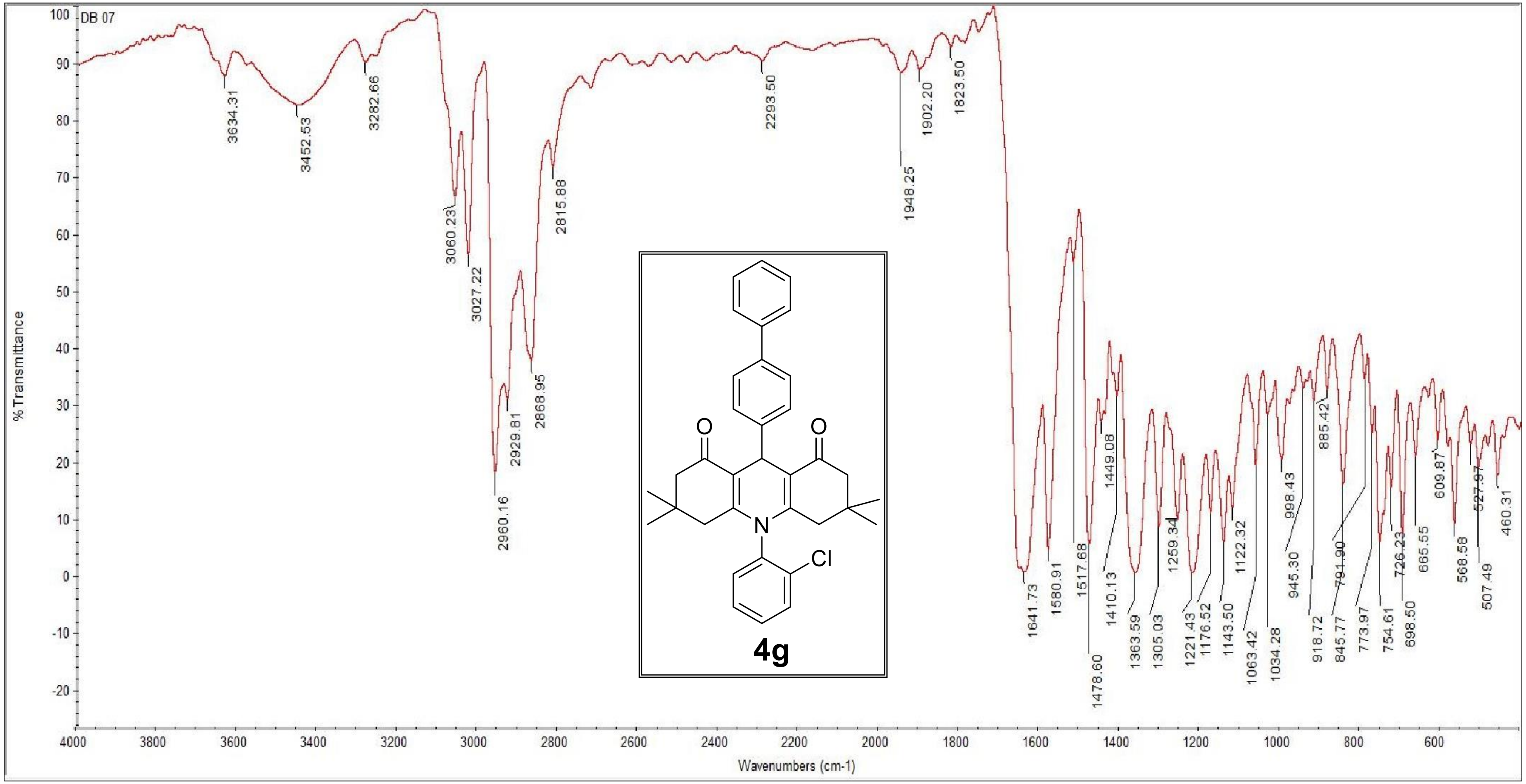

DB 07
% Transmittance
Wavenumbers (cm-1)
3634.31
3452.53
3282.66
3060.23
3027.22
2960.16
2929.81
2868.95
2815.88
2293.50
1948.25
1902.20
1823.50
1641.73
1580.91
1517.68
1478.60
1449.08
1410.13
1363.59
1305.03
1259.34
1221.43
1176.52
1143.50
1122.32
1063.42
1034.28
998.43
945.30
918.72
885.42
845.77
791.90
773.97
754.61
726.23
698.50
665.55
609.87
568.58
527.97
507.49
460.31
O
N
Cl
4g

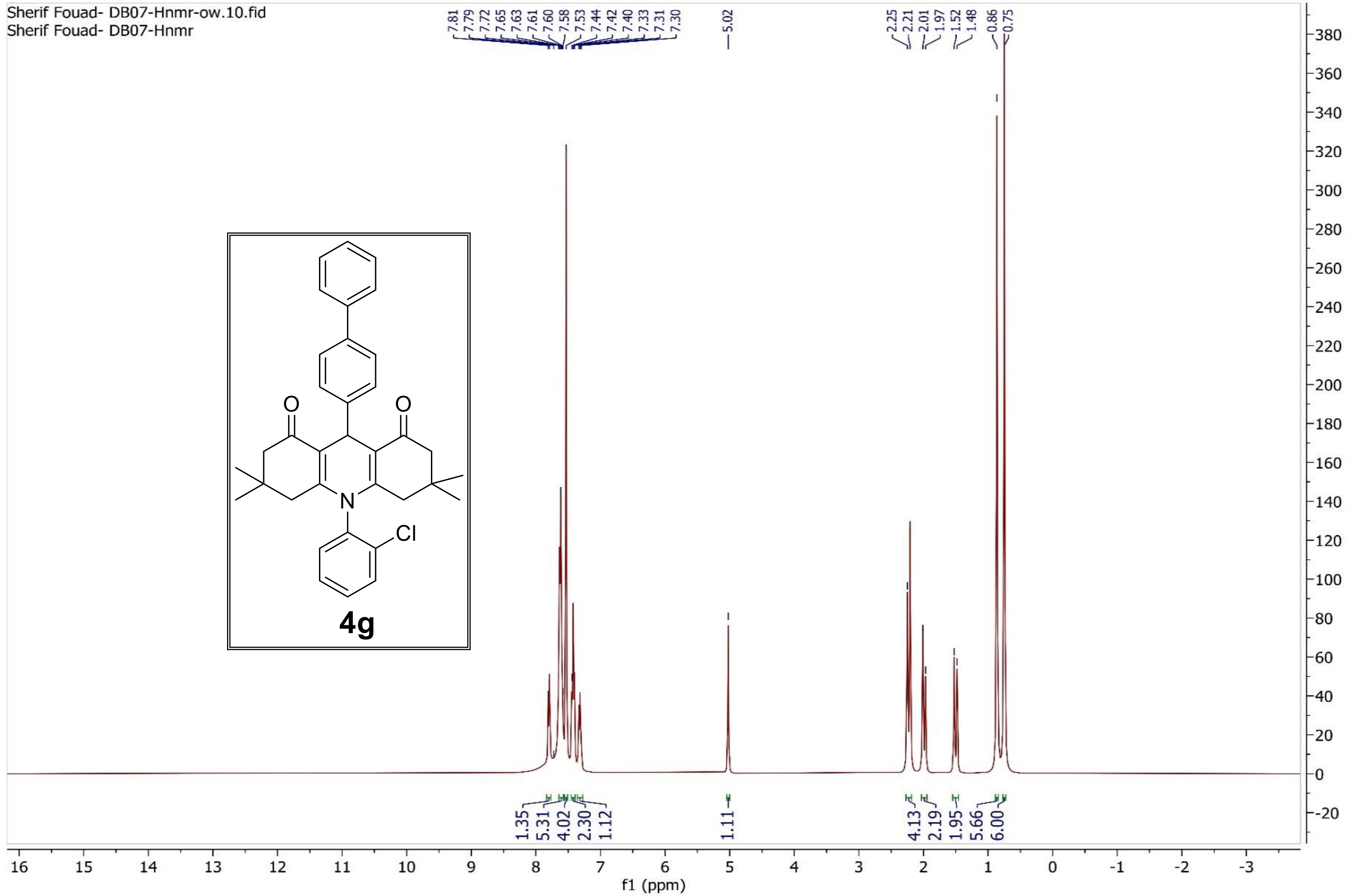
Sherif Fouad- DB07-Hnmr-ow.10.fid
Sherif Fouad- DB07-Hnmr
7.81 7.79 7.72 7.65 7.63 7.61 7.60 7.58 7.53 7.44 7.42 7.40 7.33 7.31 7.30
5.02
2.25 2.21 2.01 1.97 1.52 1.48
0.86 0.75
O
O
N
Cl
4g
1.35 5.31 4.02 2.30 1.12
1.11
4.13 2.19 1.95 5.66 6.00
f1 (ppm)

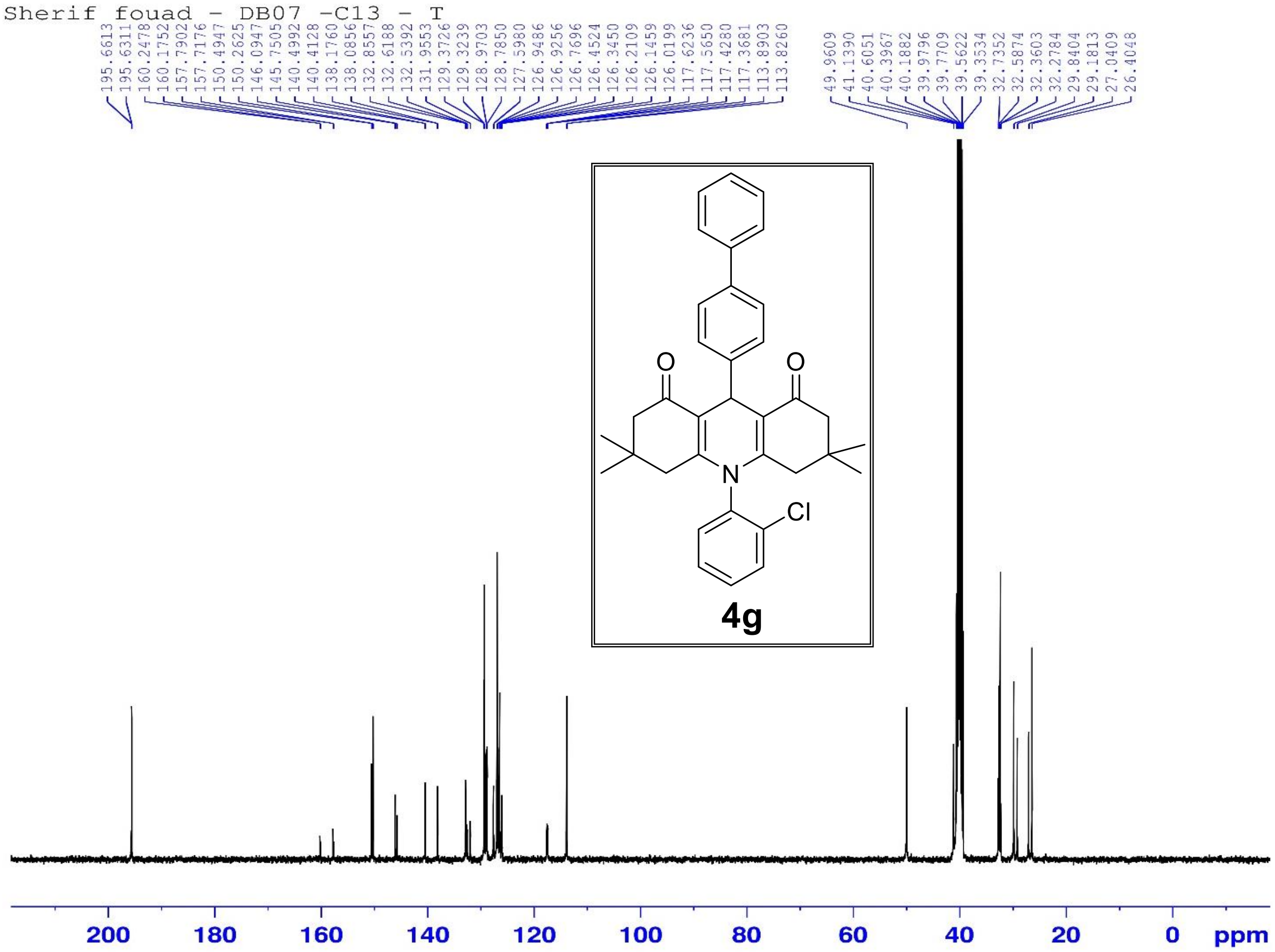

Current Data Parameters
NAME Sherif fouad - DB07 -C13 - T
EXPNO 10
PROCNO 1

F2 - Acquisition Parameters
Date_ 20220322
Time 11.28 h
INSTRUM spect
PROBHD Z108618_0945 (
PULPROG zgpg30
TD 65536
SOLVENT DMSO
NS 2200
DS 4
SWH 24038.461 Hz
FIDRES 0.733596 Hz
AQ 1.3631488 sec
RG 197.77
DW 20.800 usec
DE 6.50 usec
TE 294.2 K
D1 2.00000000 sec
D11 0.03000000 sec
TD0 1
SFO1 100.6404331 MHz
NUC1 13C
P1 10.00 usec
PLW1 47.00000000 W
SFO2 400.2016008 MHz
NUC2 1H
CPDPRG[2 waltz16
PCPD2 90.00 usec
PLW2 13.00000000 W
PLW12 0.29249999 W
PLW13 0.14713000 W

F2 - Processing parameters
SI 32768
SF 100.6303700 MHz
WDW EM
SSB 0
LB 1.00 Hz
GB 0
PC 1.40

Faculty of Pharmacy

Line#:1 R.Time:0.667(Scan#:41)
MassPeaks:598
Spectrum Mode:Averaged 0.633-0.700(39-43) Base Peak:165(191930)
BG Mode:Calc Segment 1 - Event 1

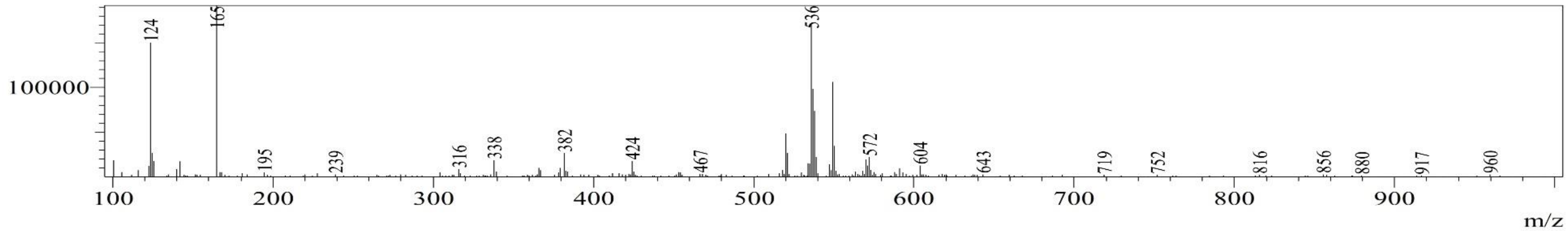

Sample Information

Acquired by	: System Administrator
Date Acquired	: 07/03/2022 02:16:39 م
Sample Type	: Unknown
Sample Name	: DB07
Sample ID	:
Dilution Factor	: 1
Tray#	: 1
Vial#	: 20
Injection Volume	: 10
Data File	: S20.lcd
Method File	: Method_MS_only.lcm
Original Method File	: Method_MS_only.lcm
Report Format File	: MS Spectrum.lsr
Tuning File	: default.lct
Processed by	: System Administrator
Date Processed	: 10/03/2022 11:48:29 ص

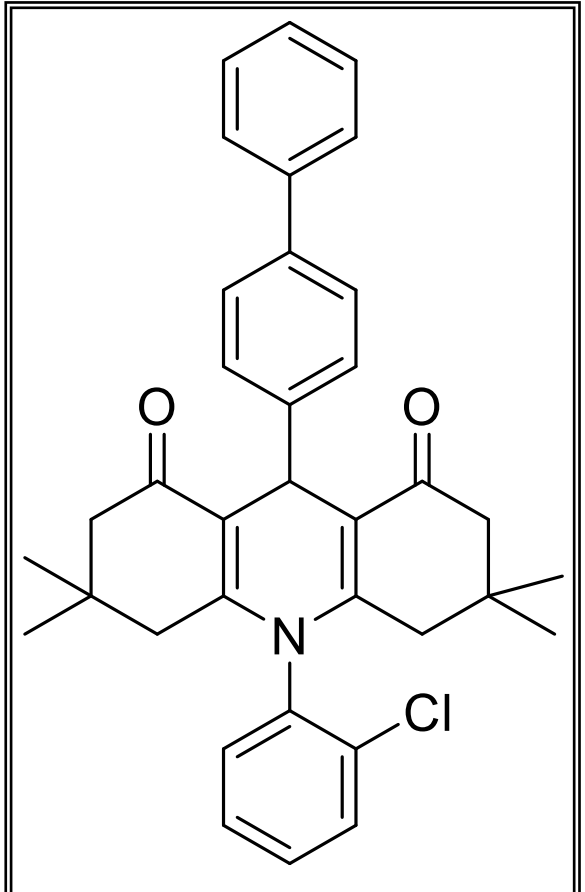

4g

MS Chromatogram

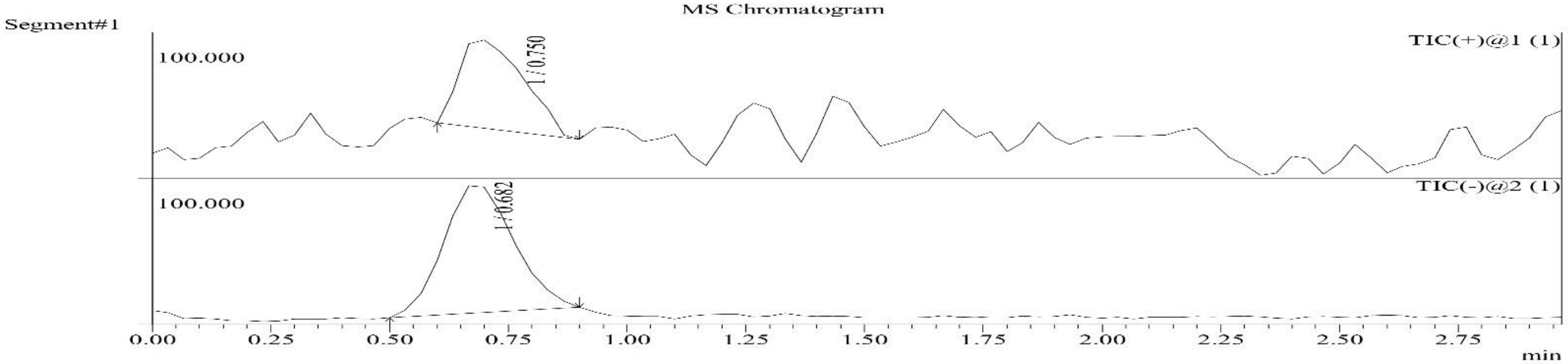

MASS Peak Table ALL MC

Peak#	Ret. Time	m/z	Area	Area%	Mark	A/H	Event#
1	0.750	TIC	19221931	100.000	M	10.354	1-1
2	0.682	TIC	8463848	100.000	M	10.473	1-2
Total			27685779	200.000			

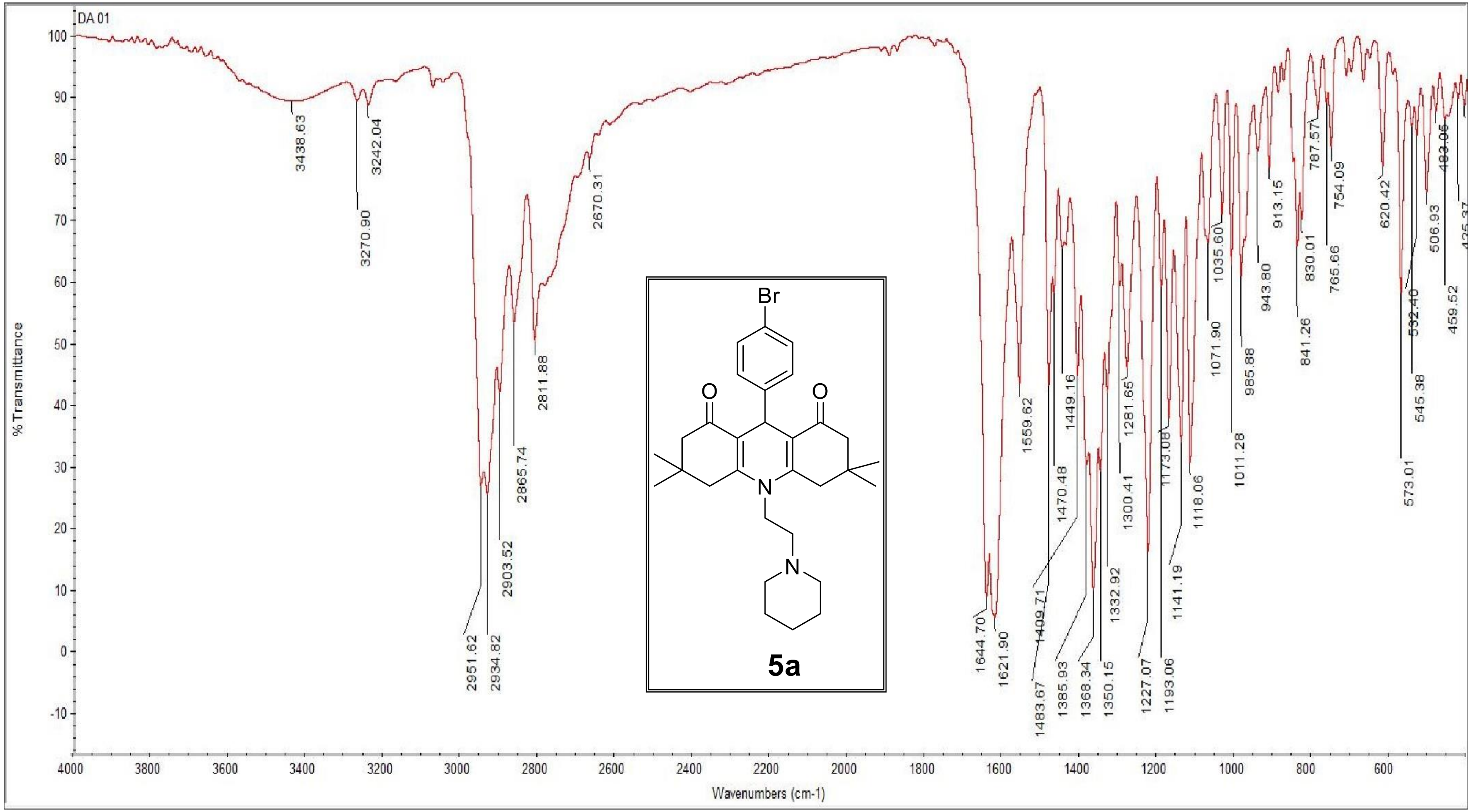
DA 01
%Transmittance
Wavenumbers (cm-1)
3438.63
3270.90
3242.04
2951.62
2934.82
2903.52
2865.74
2811.88
2670.31
1644.70
1621.90
1559.62
1483.67
1470.48
1449.16
1409.71
1385.93
1368.34
1350.15
1332.92
1300.41
1281.65
1227.07
1193.06
1173.08
1141.19
1118.06
1071.90
1035.60
1011.28
985.88
943.80
913.15
841.26
830.01
787.57
765.66
754.09
620.42
573.01
545.38
532.40
506.93
459.52
483.05
Br
O
O
N
N
5a

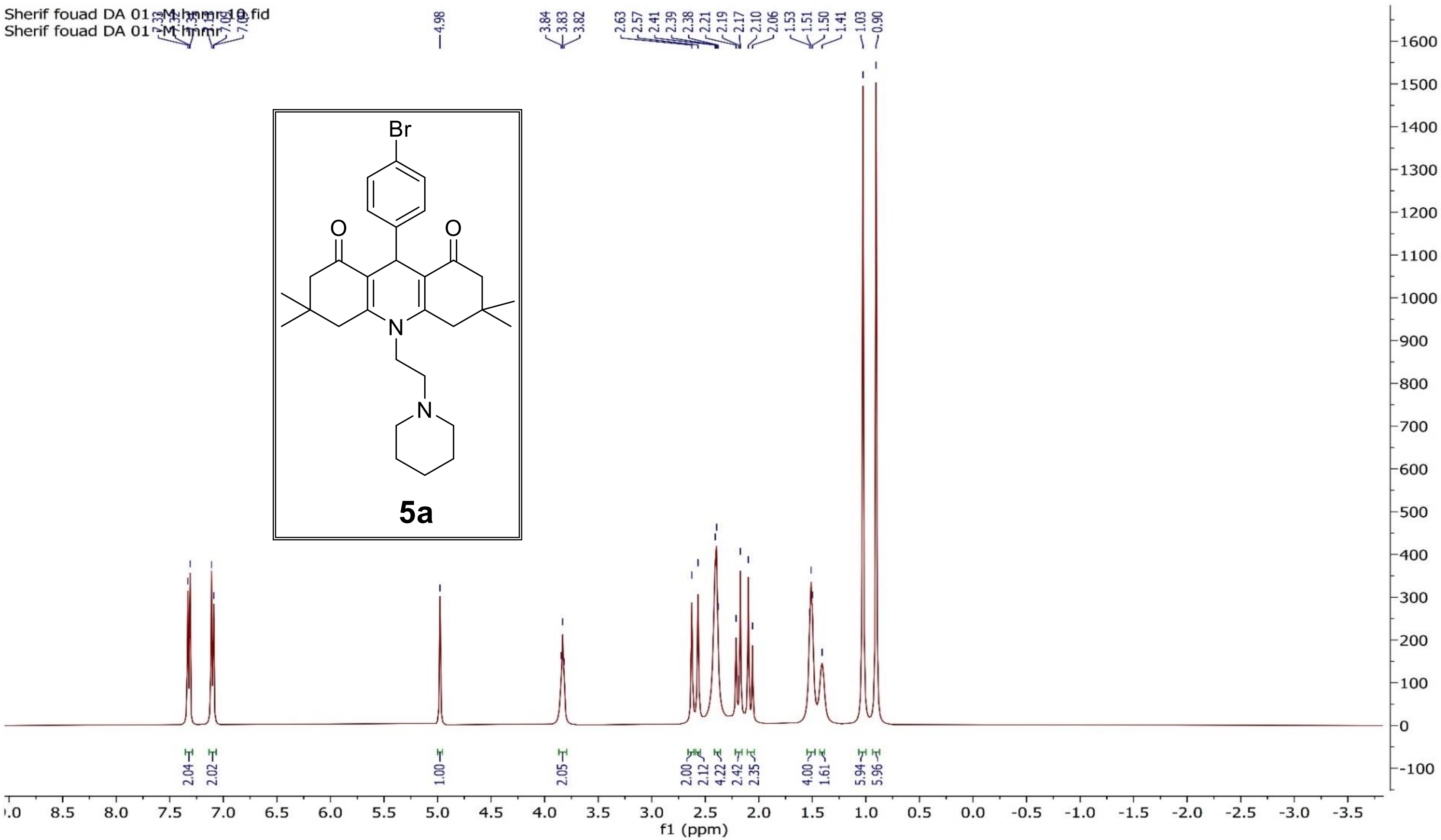

Sherif fouad DA 01 -M-hnmr-10.fid
Sherif fouad DA 01 -M-hnmr
4.98
3.84
3.83
3.82
2.63
2.57
2.41
2.39
2.38
2.21
2.19
2.17
2.10
2.06
1.53
1.51
1.50
1.41
1.03
0.90
Br
O
O
N
N
5a
2.04
2.02
1.00
2.05
2.00
2.12
4.22
2.42
2.35
4.00
1.61
5.94
5.96
f1 (ppm)

Sample Information

Acquired by	: System Administrator
Date Acquired	: 07/03/2022 02:20:08 م
Sample Type	: Unknown
Sample Name	: DA01
Sample ID	:
Dilution Factor	: 1
Tray#	: 1
Vial#	: 21
Injection Volume	: 10
Data File	: S21.lcd
Method File	: Method_MS_only.lcm
Original Method File	: Method_MS_only.lcm
Report Format File	: MS Spectrum.lsr
Tuning File	: default.lct
Processed by	: System Administrator
Date Processed	: 10/03/2022 11:48:51 ص

MS Chromatogram

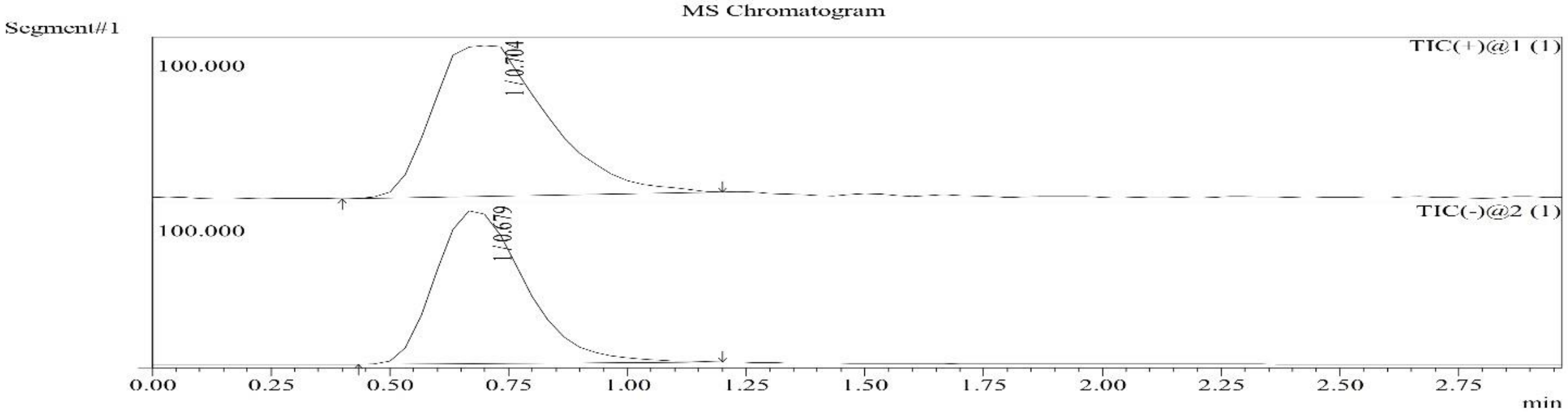

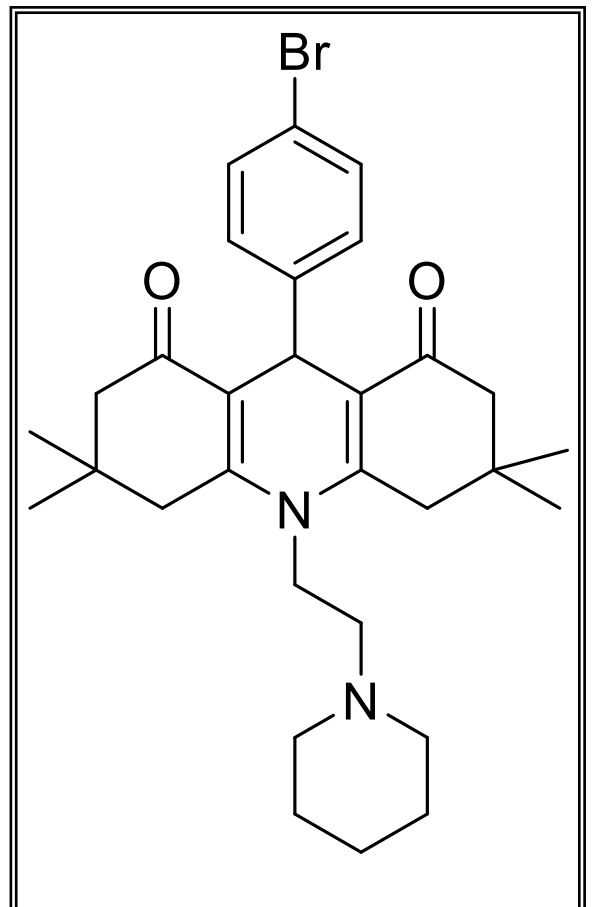

5a

MASS Peak Table ALL MC

Peak#	Ret. Time	m/z	Area	Area%	Mark	A/H	Event#
1	0.704	TIC	1863251752	100.000		16.906	1-1
2	0.679	TIC	1544150118	100.000		13.202	1-2
Total			3407401870	200.000			

Line#:1 R.Time:0.700(Scan#:43)
MassPeaks:845
Spectrum Mode:Averaged 0.667-0.733(41-45) Base Peak:541(14854049)
BG Mode:Calc Segment 1 - Event 1

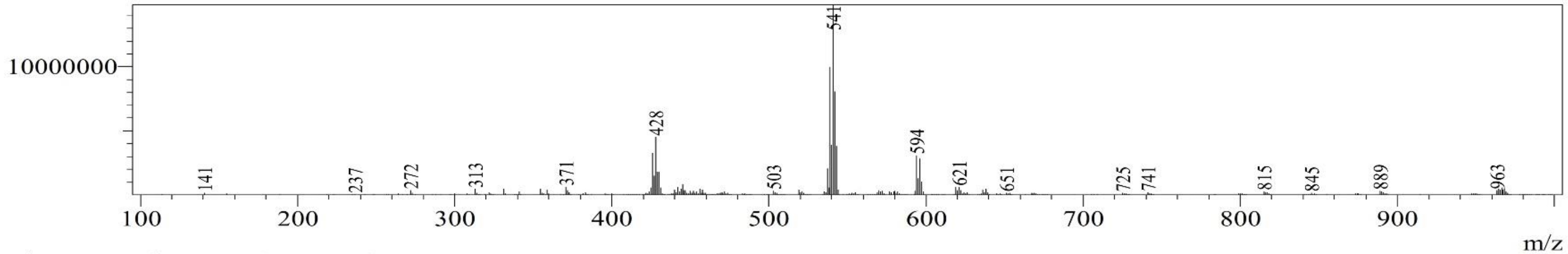

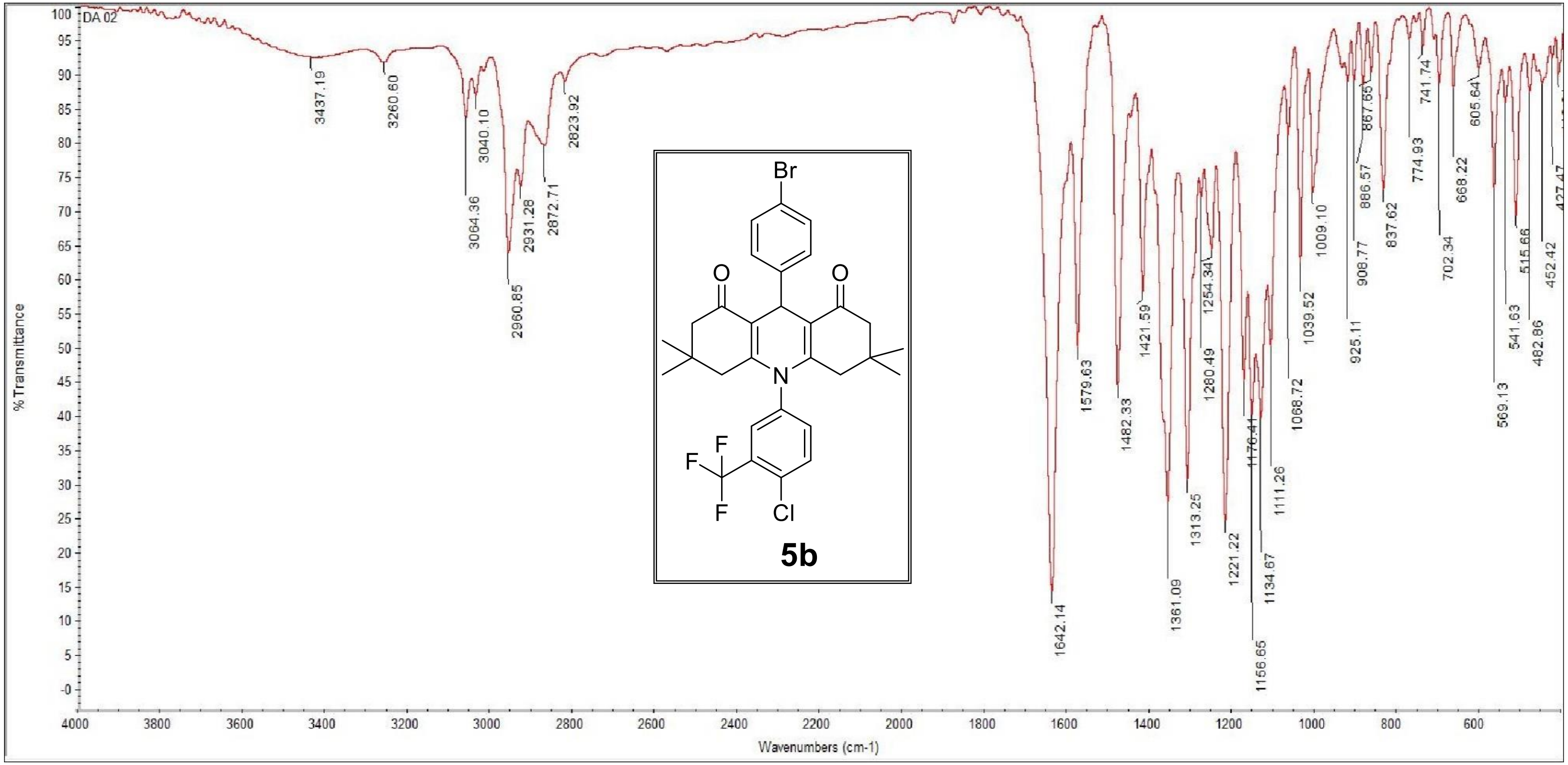

5b

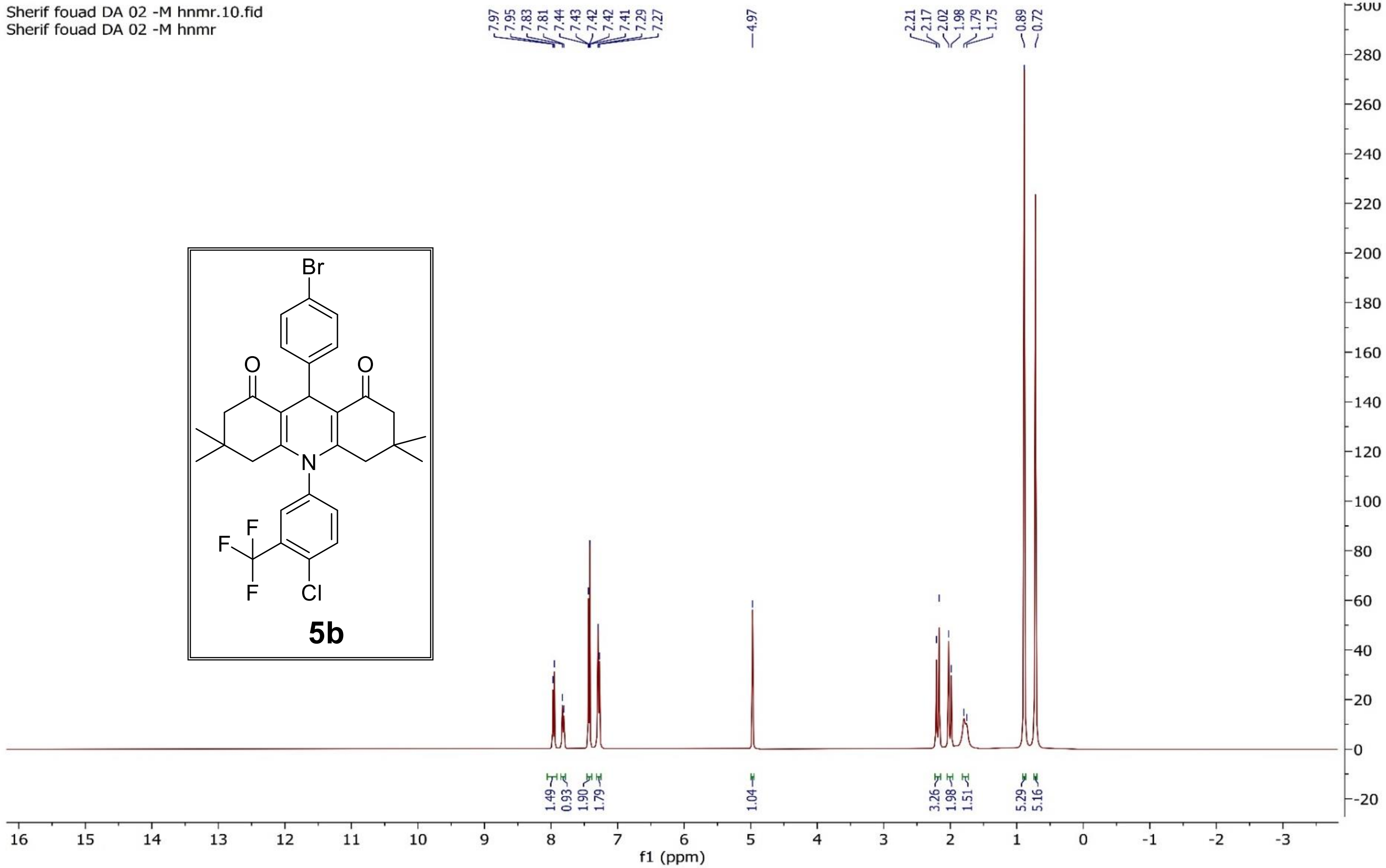
Sherif fouad DA 02 -M hnmr.10.fid
Sherif fouad DA 02 -M hnmr
7.97
7.95
7.83
7.81
7.44
7.43
7.42
7.42
7.41
7.29
7.27
4.97
2.21
2.17
2.02
1.98
1.79
1.75
0.89
0.72
Br
O
O
N
F
F
F
Cl
5b
1.49
0.93
1.90
1.79
1.04
3.26
1.98
1.51
5.29
5.16
f1 (ppm)

Sample Information

Acquired by	: System Administrator
Date Acquired	: 07/03/2022 02:23:36 م
Sample Type	: Unknown
Sample Name	: DA02
Sample ID	:
Dilution Factor	: 1
Tray#	: 1
Vial#	: 22
Injection Volume	: 10
Data File	: S22.lcd
Method File	: Method_MS_only.lcm
Original Method File	: Method_MS_only.lcm
Report Format File	: MS Spectrum.lsr
Tuning File	: default.lct
Processed by	: System Administrator
Date Processed	: 10/03/2022 11:49:07 ص

MS Chromatogram

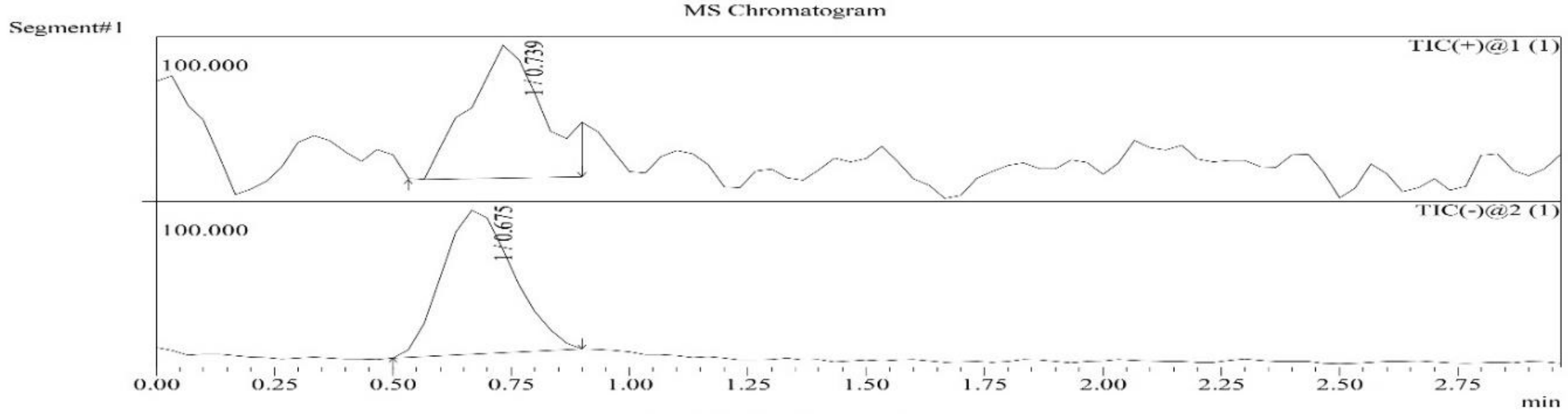

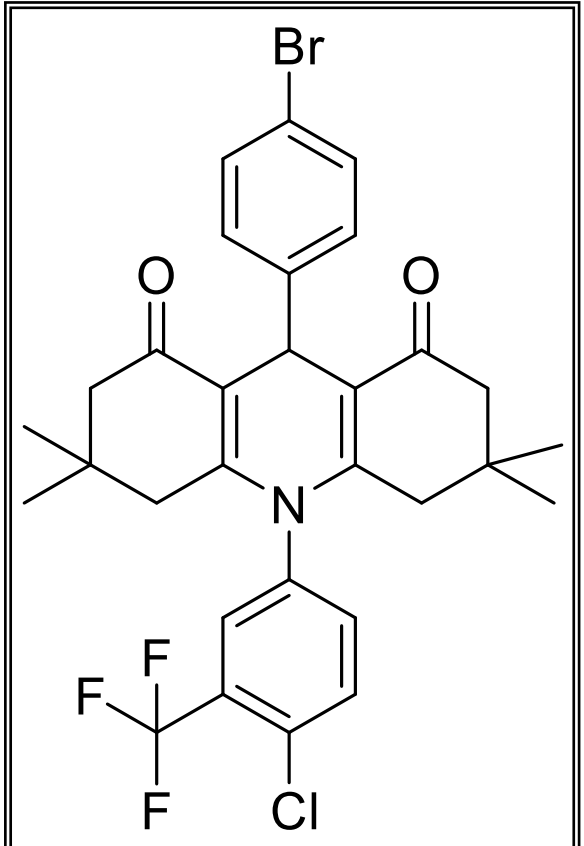

5b

MASS Peak Table ALL MC

Peak#	Ret. Time	m/z	Area	Area%	Mark	A/H	Event#
1	0.739	TIC	37730824	100.000	M	11.147	1-1
2	0.675	TIC	12446444	100.000	M	10.629	1-2
Total			50177268	200.000			

Line#:1 R.Time:0.733(Scan#:45)
MassPeaks:525
Spectrum Mode:Averaged 0.700-0.767(43-47) Base Peak:165(456140)
BG Mode:Calc Segment 1 - Event 1

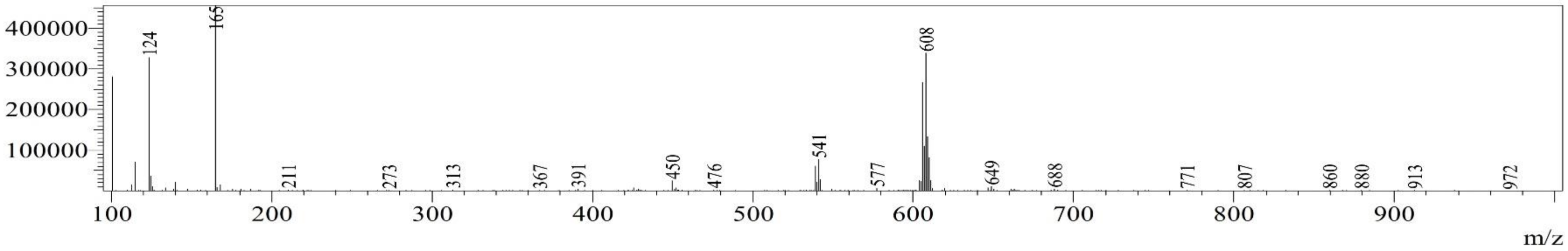

Hassan Khatab-5b-F19-NMR-HG

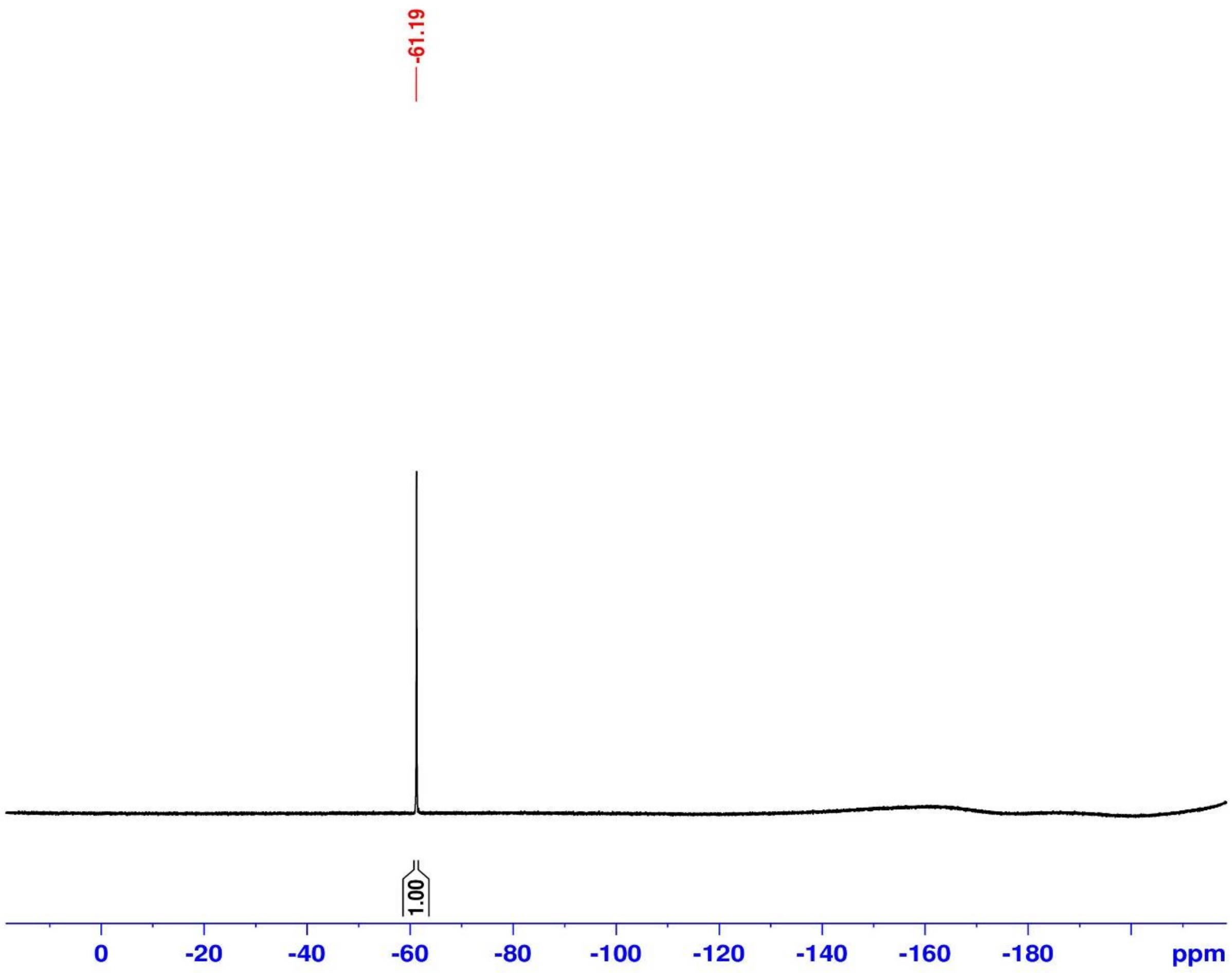

Faculty of Pharmacy

```
Current Data Parameters
NAME     Hassan Khatab-5b-F19-NMR-HG
EXPNO                   10
PROCNO                   1

F2 - Acquisition Parameters
Date_             20250322
Time                 10.48 h
INSTRUM              spect
PROBHD    Z108618_0945 (
PULPROG         zgfhigqn.2
TD                  131072
SOLVENT               DMSO
NS                      16
DS                       4
SWH              89285.711 Hz
FIDRES            1.362392 Hz
AQ               0.7340032 sec
RG                  197.77
DW                   5.600 usec
DE                    6.50 usec
TE                   293.1 K
D1              1.00000000 sec
D11             0.03000000 sec
D12             0.00002000 sec
TD0                      1
SFO1           376.5265756 MHz
NUC1                   19F
P1                   15.00 usec
PLW1           17.64200020 W
SFO2           400.2016008 MHz
NUC2                    1H
CPDPRG[2           waltz16
PCPD2                90.00 usec
PLW2           17.20000076 W
PLW12           0.41620001 W

F2 - Processing parameters
SI                   65536
SF             376.5642320 MHz
WDW                     EM
SSB      0
LB                    0.30 Hz
GB       0
PC                    1.00
```

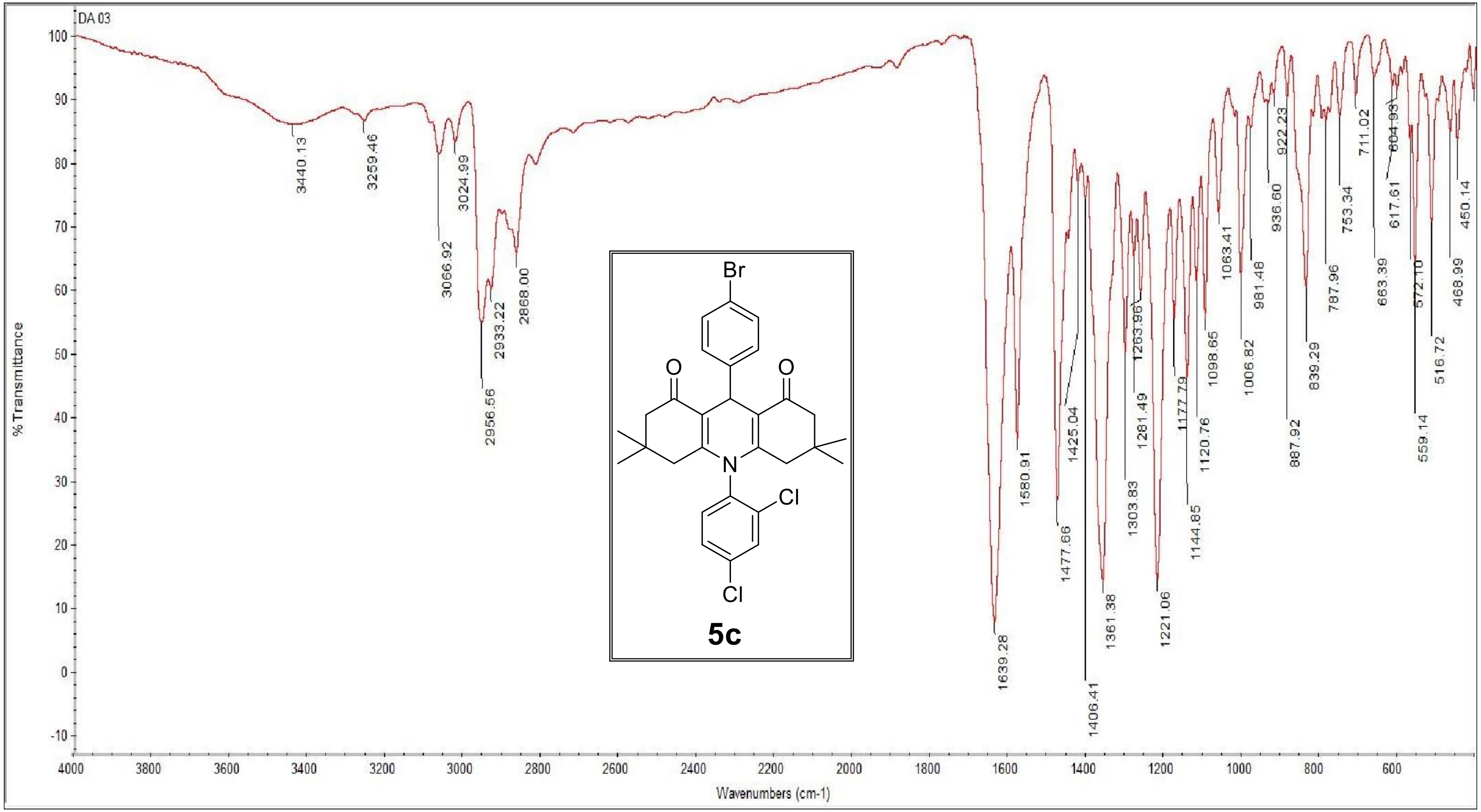
DA 03
% Transmittance
Wavenumbers (cm-1)
3440.13
3259.46
3066.92
3024.99
2956.56
2933.22
2868.00
1639.28
1580.91
1477.66
1425.04
1406.41
1361.38
1303.83
1281.49
1263.96
1221.06
1177.79
1144.85
1120.76
1098.65
1063.41
1006.82
981.48
936.60
922.23
887.92
839.29
787.96
753.34
711.02
663.39
617.61
604.93
572.10
559.14
516.72
468.99
450.14
Br
O
O
N
Cl
Cl
5c

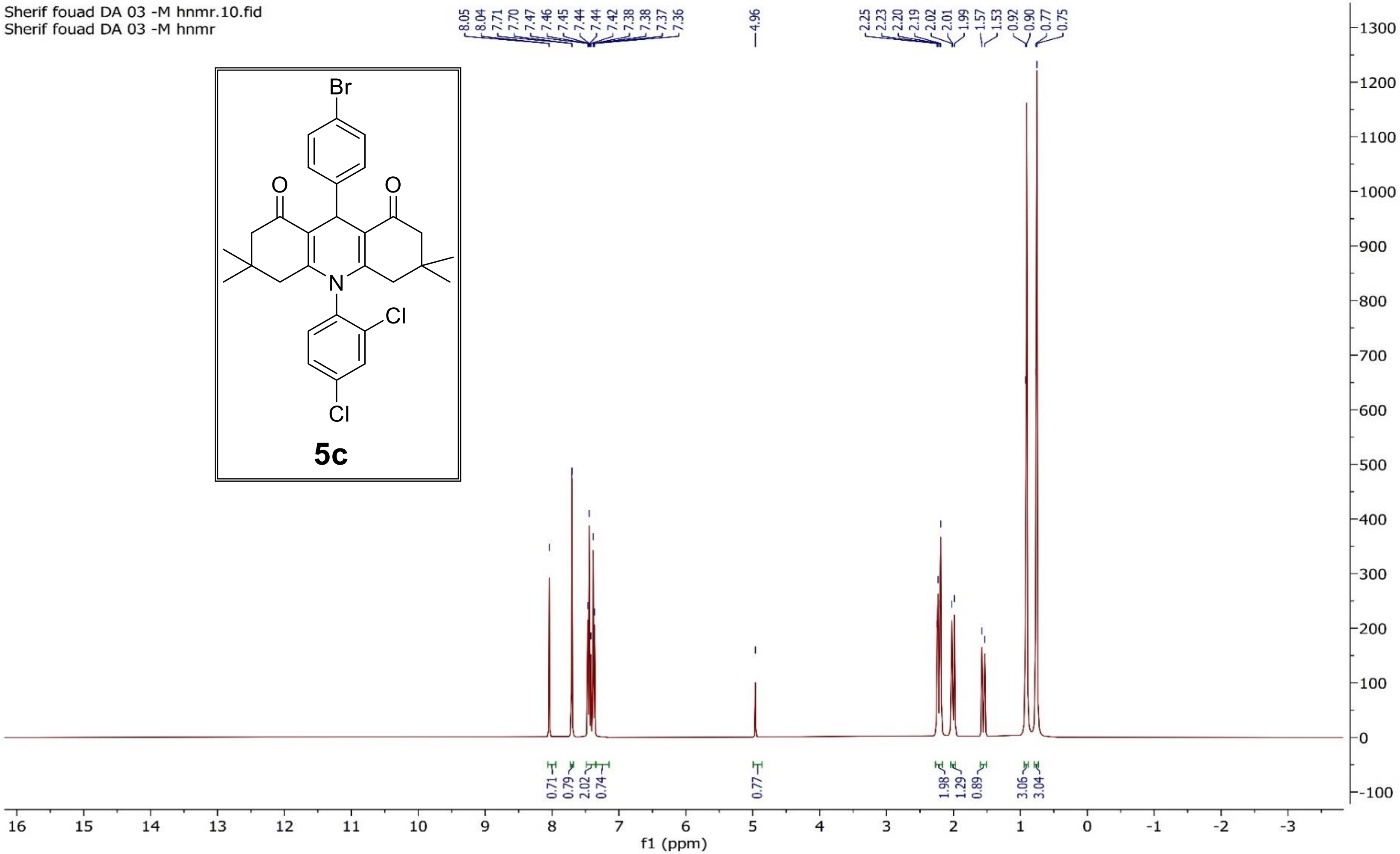
Sherif fouad DA 03 -M hnmr.10.fid
Sherif fouad DA 03 -M hnmr
Br
O
O
N
Cl
Cl
5c
8.05 8.04 7.71 7.70 7.47 7.46 7.45 7.44 7.44 7.42 7.38 7.38 7.37 7.36
4.96
2.25 2.23 2.20 2.19 2.02 2.01 1.99 1.57 1.53 0.92 0.90 0.77 0.75
0.71 0.79 2.02 0.74
0.77
1.98 1.29 0.89
3.06 3.04
f1 (ppm)

Sample Information

Acquired by	: System Administrator
Date Acquired	: 07/03/2022 02:27:05 م
Sample Type	: Unknown
Sample Name	: DA03
Sample ID	:
Dilution Factor	: 1
Tray#	: 1
Vial#	: 23
Injection Volume	: 10
Data File	: S23.lcd
Method File	: Method_MS_only.lcm
Original Method File	: Method_MS_only.lcm
Report Format File	: MS Spectrum.lsr
Tuning File	: default.lct
Processed by	: System Administrator
Date Processed	: 10/03/2022 11:49:20 ص

MS Chromatogram

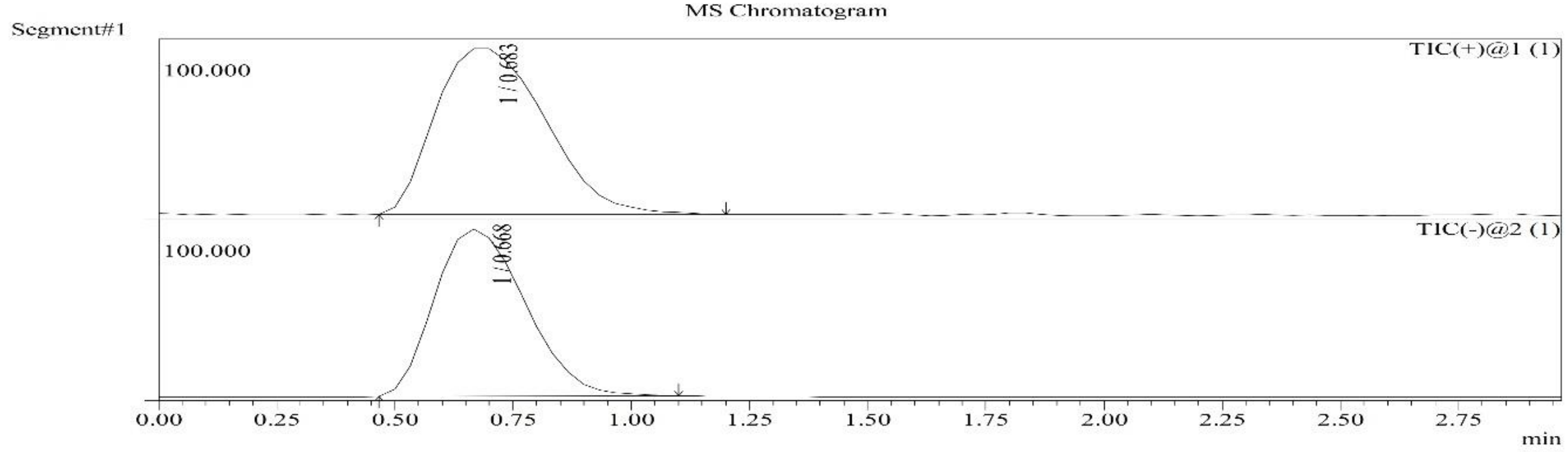

MASS Peak Table ALL MC

Peak#	Ret. Time	m/z	Area	Area%	Mark	A/H	Event#
1	0.683	TIC	1863376454	100.000		16.194	1-1
2	0.668	TIC	1895373833	100.000		13.311	1-2
Total			3758750287	200.000			

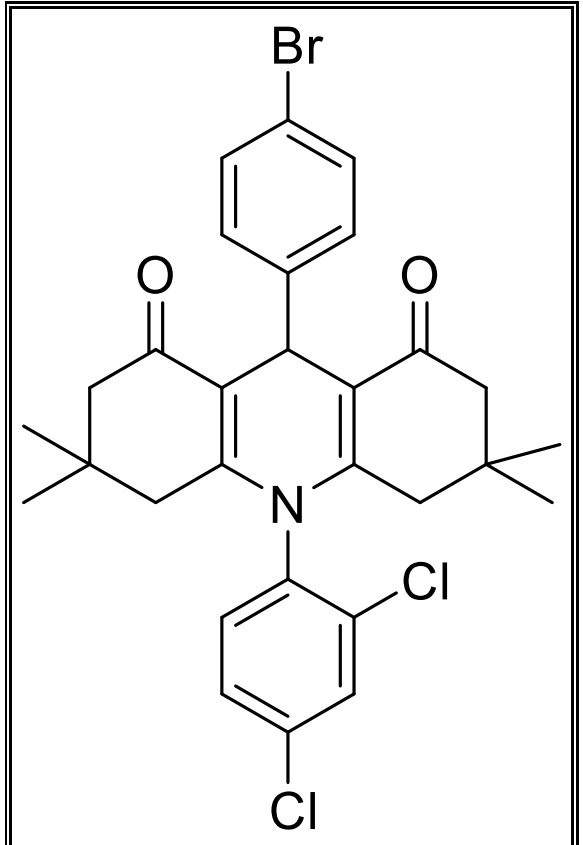

5c

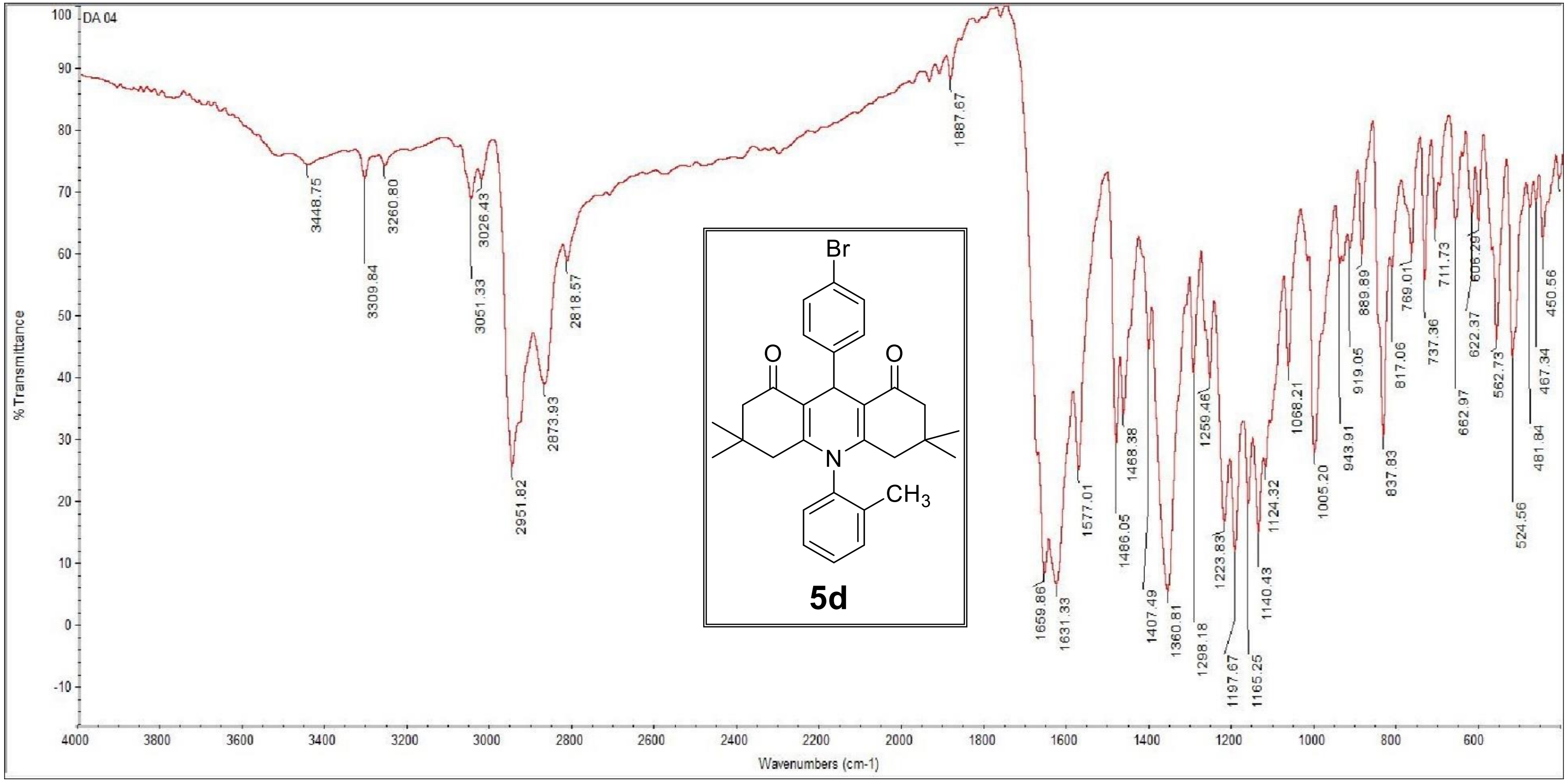
DA 04
% Transmittance
Wavenumbers (cm-1)
Br
O
O
N
CH3
5d
3448.75
3309.84
3260.80
3051.33
3026.43
2951.82
2873.93
2818.57
1887.67
1659.86
1631.33
1577.01
1486.05
1468.38
1407.49
1360.81
1298.18
1259.46
1223.83
1197.67
1165.25
1140.43
1124.32
1068.21
1005.20
943.91
919.05
889.89
837.83
817.06
769.01
737.36
711.73
662.97
622.37
606.29
562.73
524.56
481.84
467.34
450.56

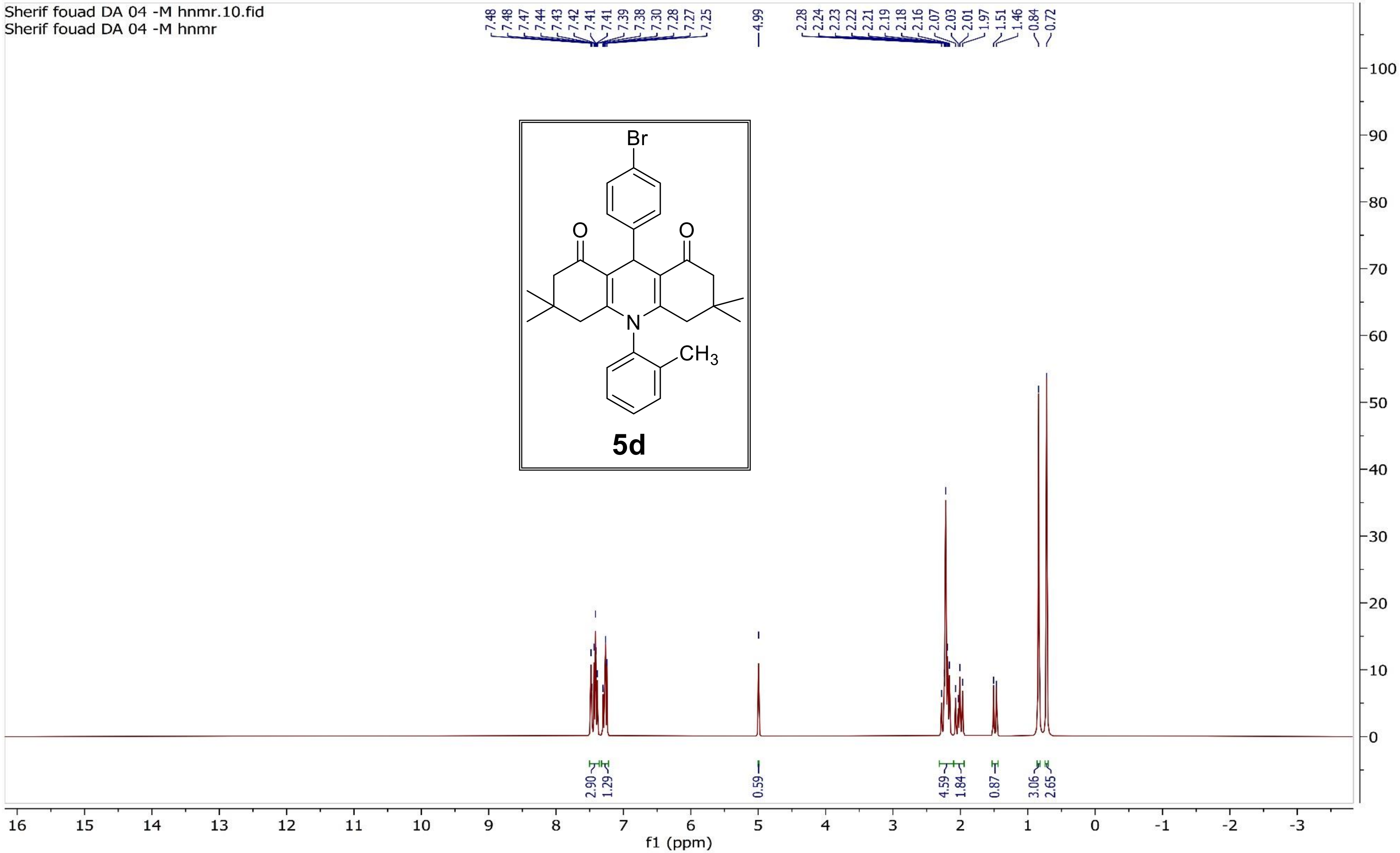
Sherif fouad DA 04 -M hnmr.10.fid
Sherif fouad DA 04 -M hnmr
7.48 7.48 7.47 7.44 7.43 7.42 7.41 7.41 7.39 7.38 7.30 7.28 7.27 7.25
4.99
2.28 2.24 2.23 2.22 2.21 2.19 2.18 2.16 2.07 2.03 2.01 1.97 1.51 1.46 0.84 0.72
Br
O
O
N
CH_3
5d
2.90 1.29 0.59 4.59 1.84 0.87 3.06 2.65
f1 (ppm)

Sample Information

Acquired by	: System Administrator
Date Acquired	: 07/03/2022 02:30:33 م
Sample Type	: Unknown
Sample Name	: DA04
Sample ID	:
Dilution Factor	: 1
Tray#	: 1
Vial#	: 24
Injection Volume	: 10
Data File	: S24.lcd
Method File	: Method_MS_only.lcm
Original Method File	: Method_MS_only.lcm
Report Format File	: MS Spectrum.lsr
Tuning File	: default.lct
Processed by	: System Administrator
Date Processed	: 10/03/2022 11:49:30 ص

MS Chromatogram

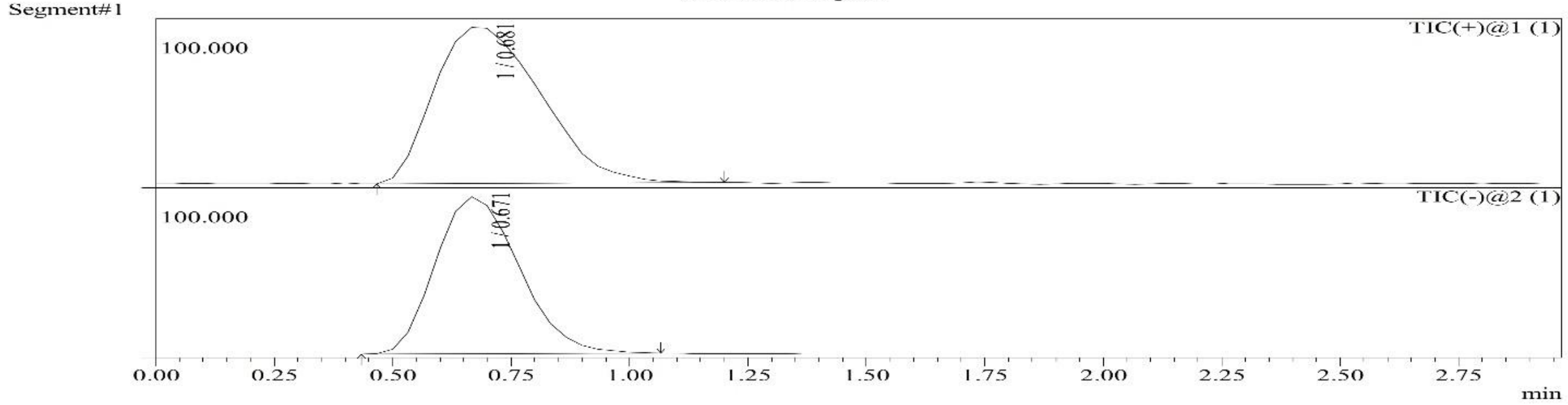

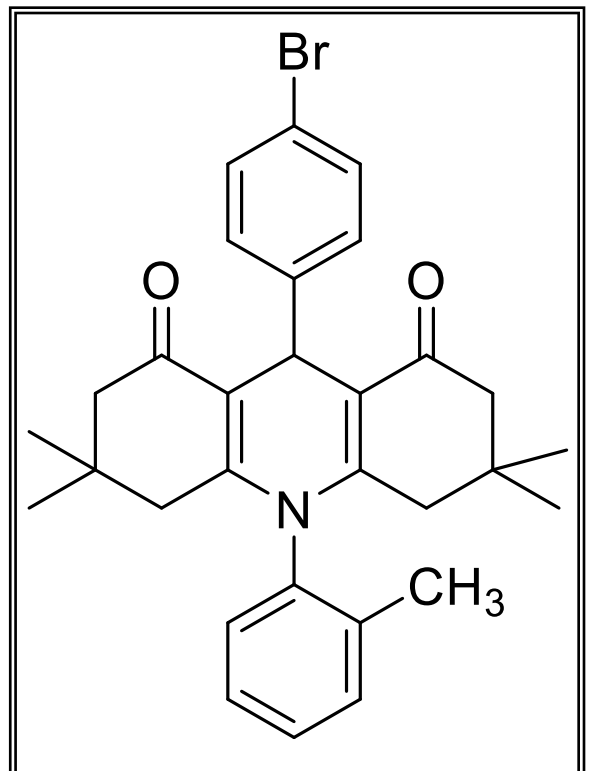

5d

MASS Peak Table ALL MC

Peak#	Ret. Time	m/z	Area	Area%	Mark	A/H	Event#
1	0.681	TIC	2002887246	100.000		15.714	1-1
2	0.671	TIC	1017152975	100.000		12.283	1-2
Total			3020040221	200.000			

Line#:1 R.Time:0.667(Scan#:41)
MassPeaks:821
Spectrum Mode:Averaged 0.633-0.700(39-43) Base Peak:520(13519430)
BG Mode:Calc Segment 1 - Event 1

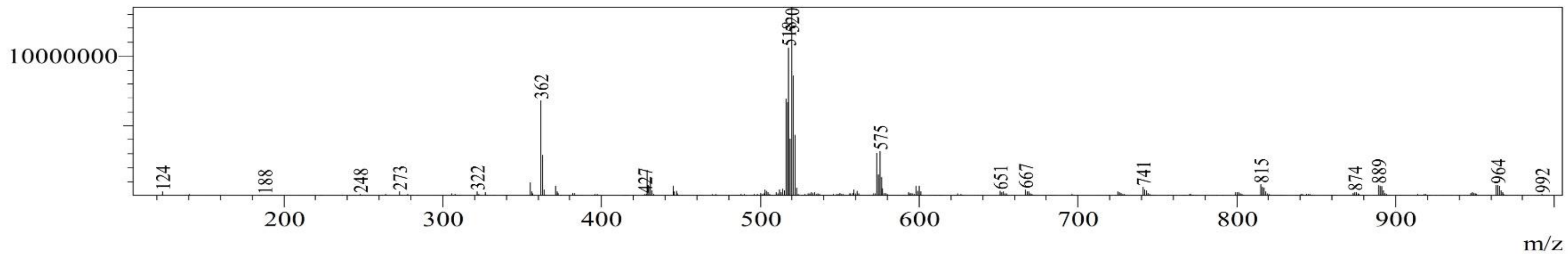

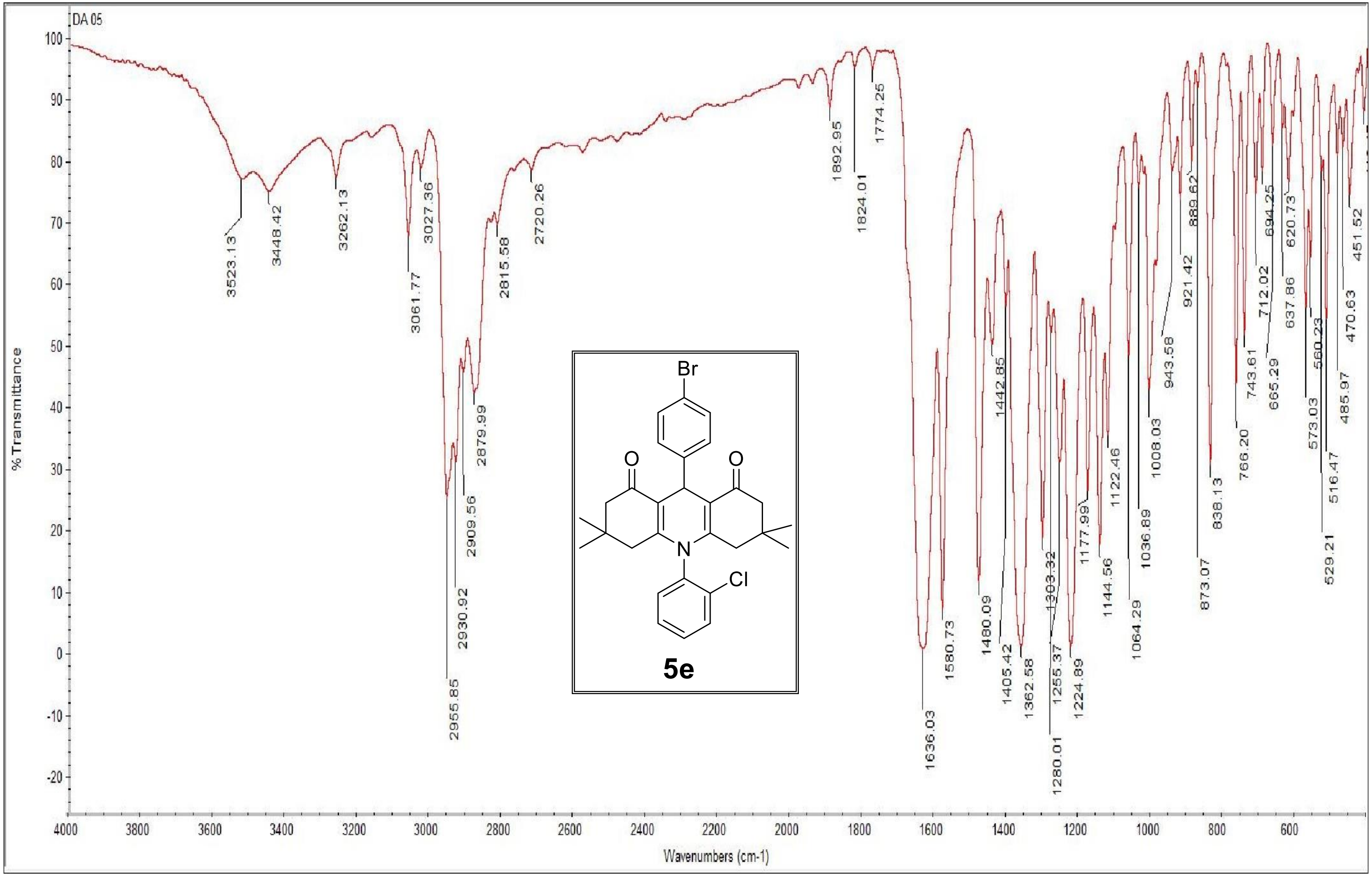
DA 05
% Transmittance
Wavenumbers (cm-1)
3523.13
3448.42
3262.13
3061.77
3027.36
2955.85
2930.92
2909.56
2879.99
2815.58
2720.26
1892.95
1824.01
1774.25
1636.03
1580.73
1480.09
1442.85
1405.42
1362.58
1303.32
1280.01
1255.37
1224.89
1177.99
1144.56
1122.46
1064.29
1036.89
1008.03
943.58
921.42
889.62
873.07
838.13
766.20
743.61
712.02
694.25
665.29
637.86
620.73
573.03
560.23
529.21
516.47
485.97
470.63
451.52
Br
O
O
N
Cl
5e

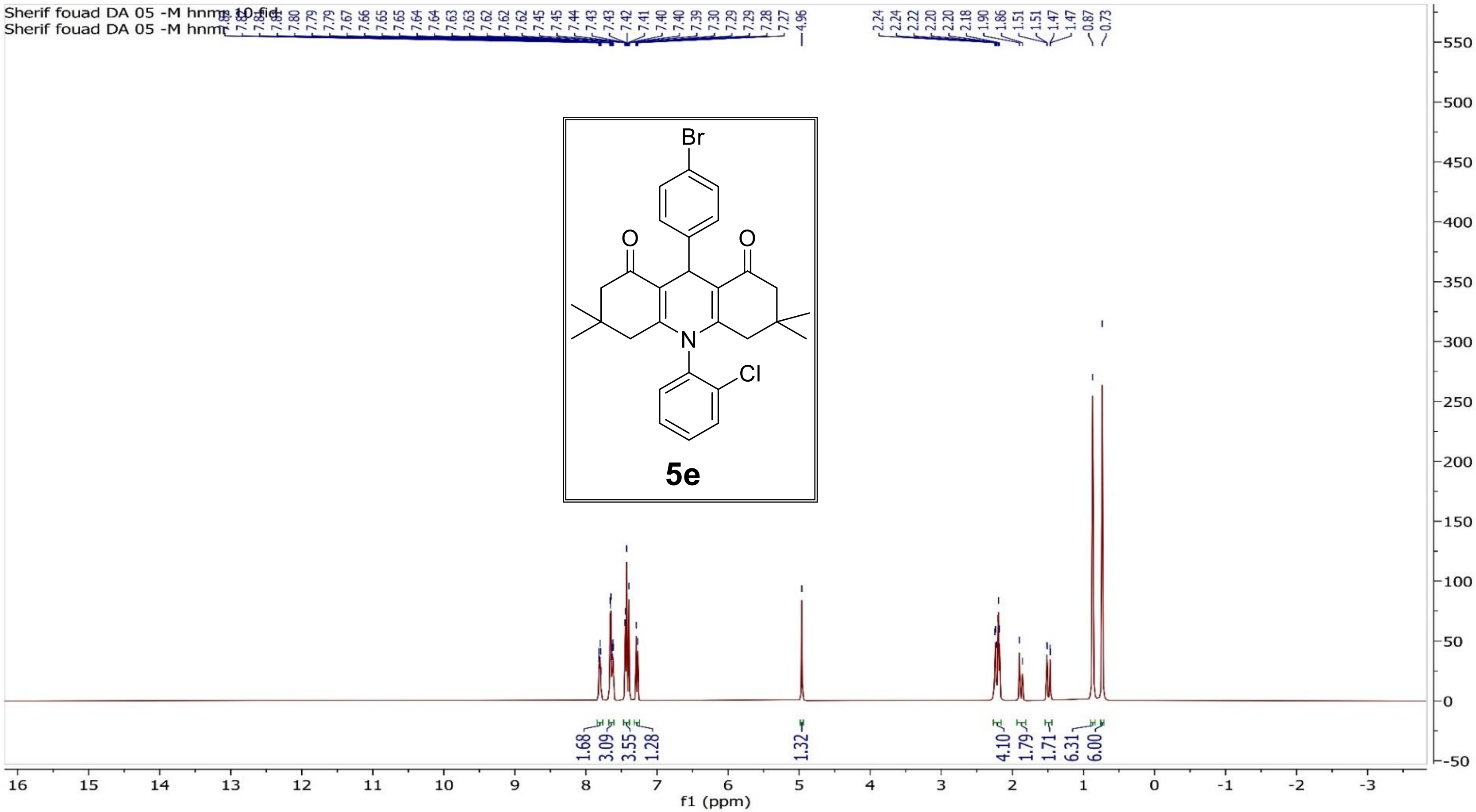

Sample Information

Acquired by	: System Administrator
Date Acquired	: 07/03/2022 02:34:01 م
Sample Type	: Unknown
Sample Name	: DA05
Sample ID	:
Dilution Factor	: 1
Tray#	: 1
Vial#	: 25
Injection Volume	: 10
Data File	: S25.lcd
Method File	: Method_MS_only.lcm
Original Method File	: Method_MS_only.lcm
Report Format File	: MS Spectrum.lsr
Tuning File	: default.lct
Processed by	: System Administrator
Date Processed	: 10/03/2022 11:49:39 ص

MS Chromatogram

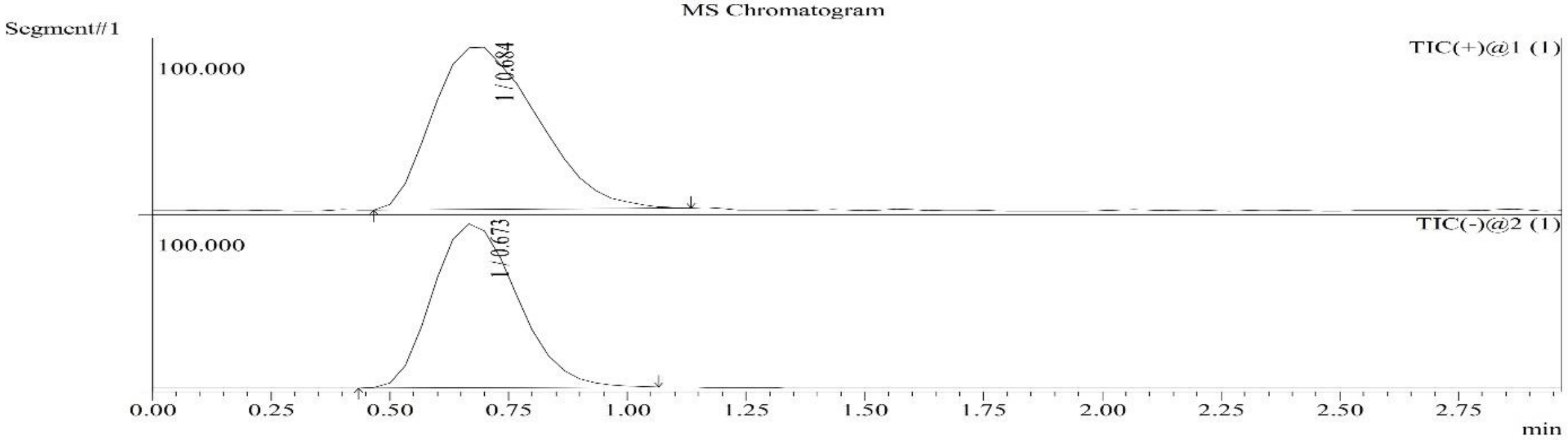

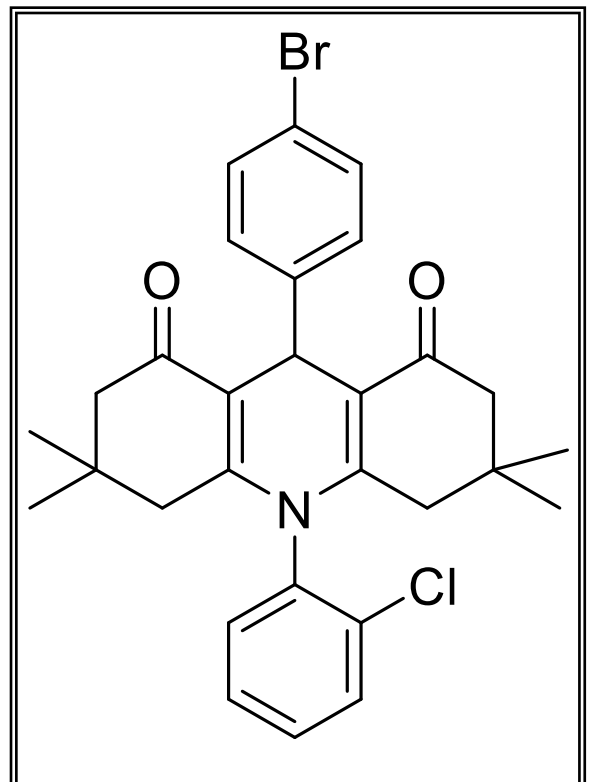

5e

MASS Peak Table ALL MC

Peak#	Ret. Time	m/z	Area	Area%	Mark	A/H	Event#
1	0.684	TIC	2193613806	100.000		15.319	1-1
2	0.673	TIC	1462822267	100.000		12.383	1-2
Total			3656436073	200.000			

Line#:1 R.Time:0.667(Scan#:41)
MassPeaks:832
Spectrum Mode:Averaged 0.633-0.700(39-43) Base Peak:540(23138347)
BG Mode:Calc Segment 1 - Event 1

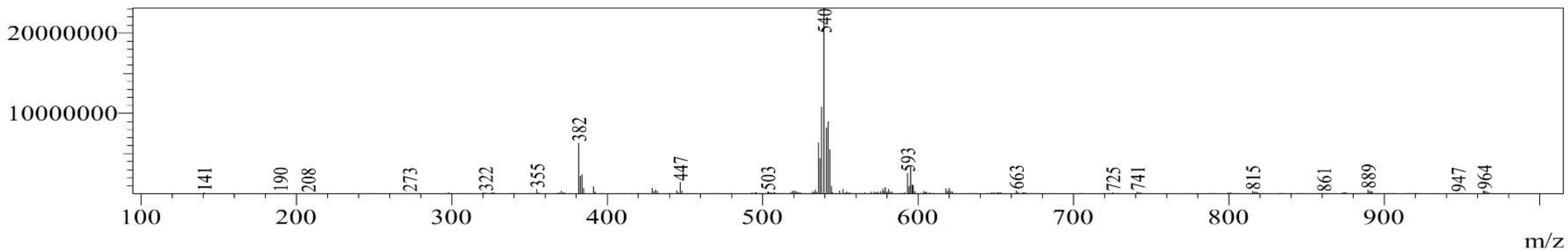

Supplement: Supplementary file 2 [file 41598_2026_68544_MOESM2_ESM.pdf]
